# Steric Control of Luminescence in Phenyl-Substituted Trityl Radicals

**DOI:** 10.1021/jacs.4c00292

**Published:** 2024-05-02

**Authors:** Petri Murto, Biwen Li, Yao Fu, Lucy E. Walker, Laura Brown, Andrew D. Bond, Weixuan Zeng, Rituparno Chowdhury, Hwan-Hee Cho, Craig P. Yu, Clare P. Grey, Richard H. Friend, Hugo Bronstein

**Affiliations:** † Yusuf Hamied Department of Chemistry, University of Cambridge, Cambridge CB2 1EW, U.K.; ‡ Cavendish Laboratory, University of Cambridge, Cambridge CB3 0HE, U.K.

## Abstract

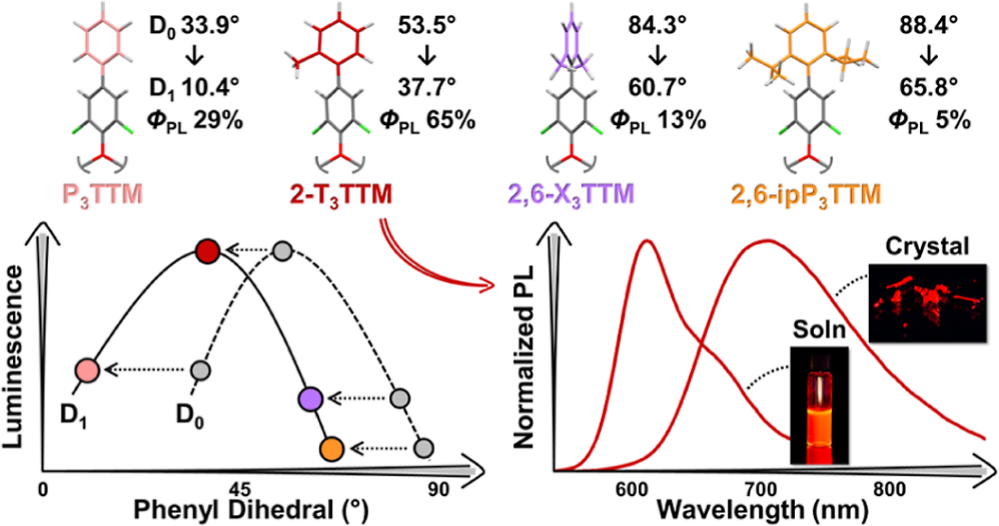

Triphenylmethyl (trityl) radicals have shown potential for use in organic optoelectronic applications, but the design of practical trityl structures has been limited to donor/radical charge-transfer systems due to the poor luminescence of alternant symmetry hydrocarbons. Here, we circumvent the symmetry-forbidden transition of alternant hydrocarbons via excited-state symmetry breaking in a series of phenyl-substituted tris(2,4,6-trichlorophenyl)methyl (TTM) radicals. We show that 3-fold phenyl substitution enhances the emission of the TTM radical and that steric control modulates the optical properties in these systems. Simple *ortho*-methylphenyl substitution boosts the photoluminescence quantum efficiency from 1% (for TTM) to 65% at a peak wavelength of 612 nm (for 2-T_3_TTM) in solution. In the crystalline solid state, the neat 2-T_3_TTM radical shows a remarkably high photoluminescence quantum efficiency of 25% for emission peaking at 706 nm. This has implications in the design of aryl-substituted radical structures where the electronic coupling of the substituents influences variables such as emission, charge transfer, and spin interaction.

## Introduction

**Figure 1:**
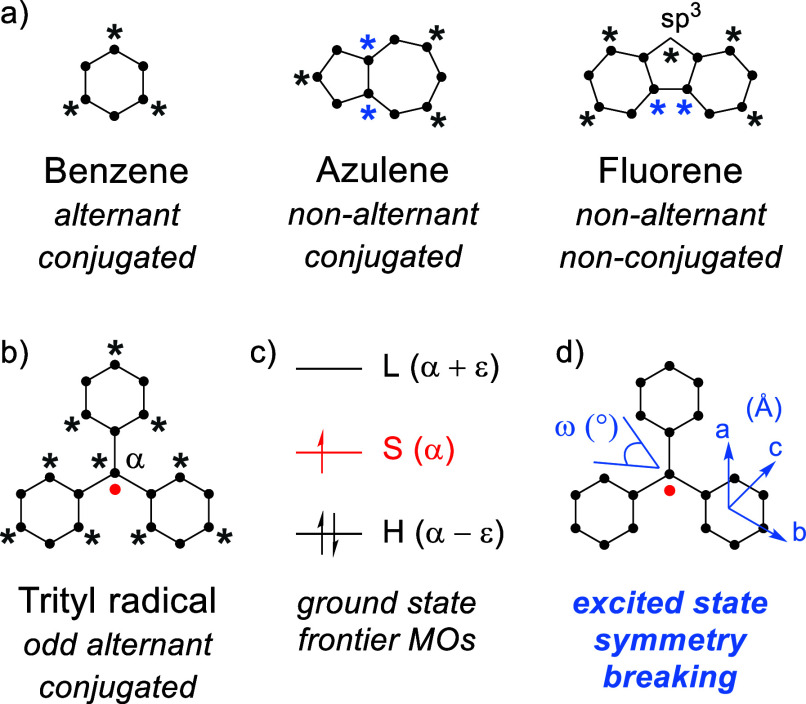
(a) Examples of alternant and nonalternant hydrocarbons where sp^2^-hybridized carbon atoms are marked with black dots. In an alternant system, conjugated atoms can be divided into two sets, starred and unstarred, such that no two atoms in the same set are directly linked to one another. (b) In the trityl radical, alternant symmetry is achieved by an odd number of conjugated atoms due to the half-filled molecular orbital at the α-carbon. Red dot represents a radical electron. (c) Schematic ground-state frontier molecular orbital diagram of the trityl radical where the occupied and unoccupied molecular orbitals are separated from the half-filled molecular orbital by an equal but opposite energy, – ε and + ε, respectively (S, SOMO; H, HOMO; L, LUMO). Electron occupancy is shown by half-headed arrows. (d) Illustration of changes in dihedrals and bond lengths that can lead to reduced, broken symmetry in the excited state of the trityl radical.

Neutral π-radicals with an emissive doublet excited state (D_1_) are of interest for applications in photonics and light-emitting devices^1−4^ and magnetically and optically addressable quantum systems^5−8^ because of the absence of energetically low-lying nonemissive states in these materials. Chlorinated triphenylmethyl (trityl) radicals like tris(2,4,6-trichlorophenyl)methyl (TTM) are among the most studied spin doublet systems largely because of their remarkable stability up to years in ambient air.^9,10^ Previously, trityl radicals had been classified as dark with photoluminescence quantum efficiencies (PLQEs) not higher than few percent.^11−15^ This is because their alternant symmetry structure gives rise to an energetically symmetric splitting of occupied and unoccupied molecular orbitals with a distinction that only their sign is opposite relative to the nonbonding, singly occupied molecular orbital (SOMO), as illustrated in Figure 1. This, in turn, translates to degenerate highest occupied molecular orbital to singly occupied molecular orbital (HOMO–SOMO) and singly occupied molecular orbital to lowest unoccupied molecular orbital (SOMO–LUMO) transitions and equal but opposite dipole moments, resulting in a vanishing oscillator strength for the lowest-energy (D_0_–D_1_) excitation in these structures.^16−18^ To brighten up radicals, the key is to lift the degeneracy of these transitions. Much enhanced emission has been obtained from charge-transfer (CT) systems where the radical has been covalently coupled to nonalternant hydrocarbon structures like carbazole with a nitrogen heteroatom introducing a nonbonding, lone electron pair to the π-system.^19−22^

Contrary to the previous knowledge, we have recently introduced an alternative approach where symmetric radicals can be made emissive if their symmetry is broken in the excited state.^23^ Similar observations have been made in some substantially bulky aryl-substituted trityl radicals, suggesting that excited-state symmetry breaking may be sterically controlled.^24−27^ However, the extent of symmetry breaking in radical emitters is not commonly understood nor thoroughly studied, and for this, one may take advantage of asymmetric changes in dihedrals and bond lengths, that is, perturbation of the lowest-energy electronic transitions in the π-systems (Figure 1d). Significant progress in this regard would greatly expand the design of luminescent radical materials, both those of alternant symmetry structures and those of CT-type nonalternant hydrocarbon structures.

Herein, we report a systematic series of phenyl-substituted TTM radicals with varied degrees of steric bulk. Introduction of methyl groups to the phenyl rings mixes their molecular orbital energy structure as illustrated in Figure S1, whereas SOMO associated with the radical center remains energetically unchanged (Figure S2). Coupling these phenyl substituents from different positions to TTM allows building of a family of radicals whose luminescence is controlled both sterically and electronically. Similar modification of phenyl ligands by methyl groups has been demonstrated to affect the ground-state spin structure of organometallic (nonradical) spin–optical interfaces.^28,29^ We show that subtle changes in sterics can significantly influence the luminescence in spin radicals and that their PLQEs do not strictly follow the energy gap law.^30^

## Results and Discussion

**Scheme 1:**
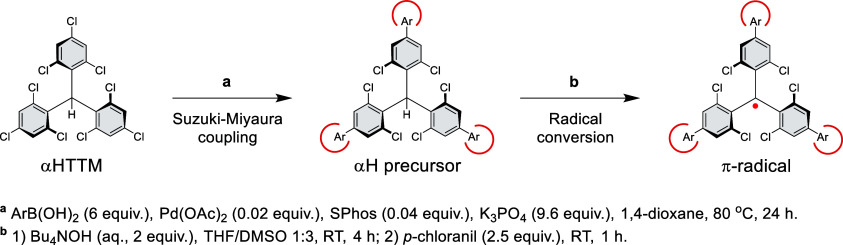
Synthesis of Phenyl-Substituted Radical Derivatives

The phenyl-substituted radicals were obtained via Suzuki–Miyaura (S–M) coupling of αHTTM and the respective arylboronic acid (ArB(OH)_2_) in mild anhydrous conditions,^31^ followed by radical conversion through deprotonation and one-electron oxidation, as outlined in Scheme 1. Detailed synthetic procedures and structural characterization are described in the Supporting Information. We find that anhydrous S–M reaction conditions in a polar environment (1,4-dioxane) with the SPhos ligand give αH precursor molecules without *ortho*-dehalogenation^23^ and that solubility of these products is affected by their polarity. The yields of the 3-fold S–M couplings reported in Table 1 are those of isolated products, and they reflect both the steric bulk of the aryl substituents and solubility of the αH precursor derivatives rather than the reactivity of the *para*-chlorines in these reactions. Radical conversion benefits from a highly polar environment that stabilizes the highly soluble anionic equilibrium intermediate, so that the reaction reaches completion after the oxidation step giving high yields throughout the series.

**Table 1:**
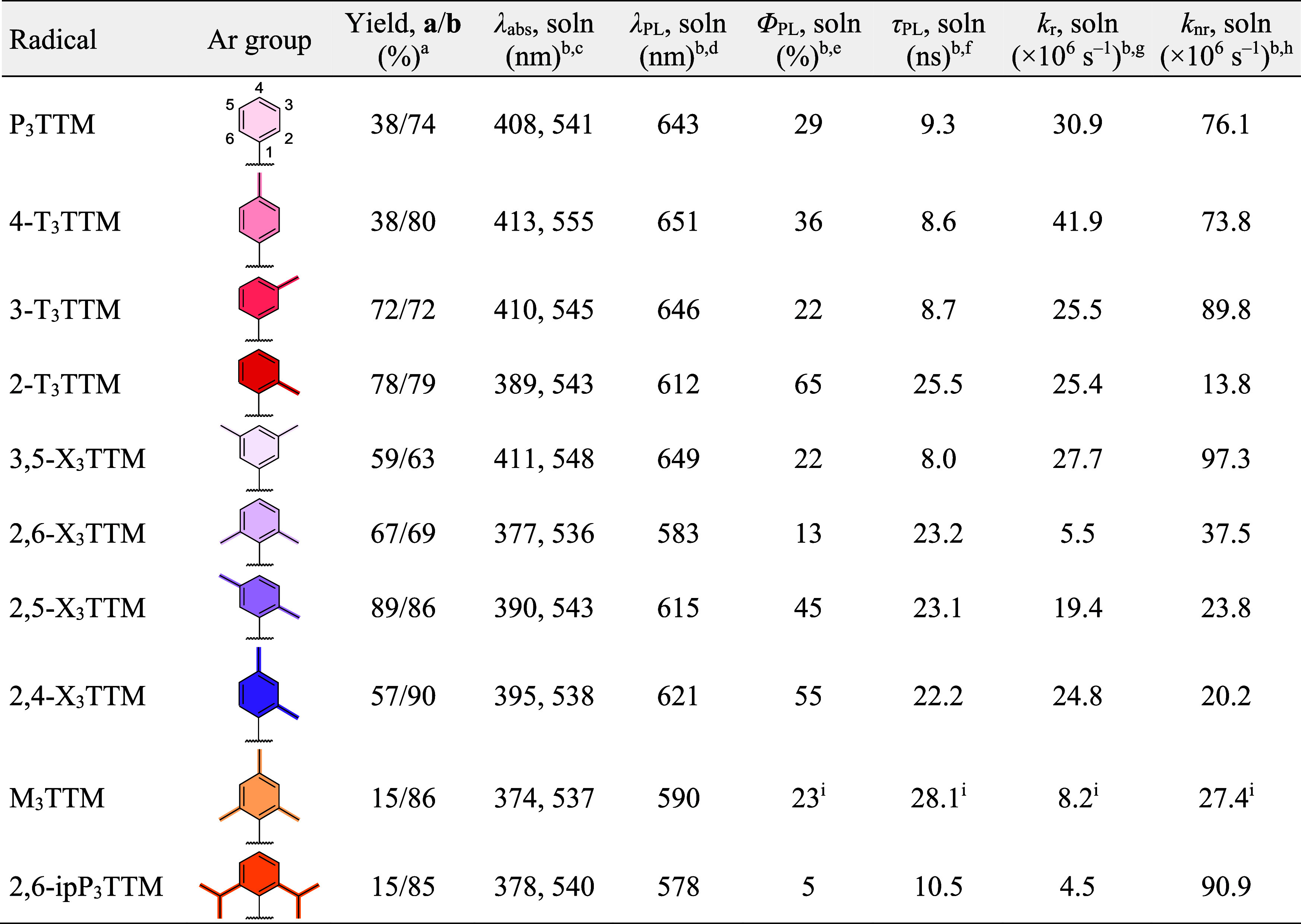
Summary of the Synthesized Radicals and Their Photophysical Parameters

a Isolated yields for synthetic steps **a** and **b** shown in Scheme 1.

b Sample in a 0.1 mM toluene solution.

c Highest- and lowest-energy peaks of UV–vis absorption.

d Photoluminescence peak wavelength.

e Photoluminescence quantum efficiency.

f Photoluminescence lifetime.

g Radiative decay rate.

h Nonradiative decay rate.

i Obtained from ref (23).

Starting from the phenyl-substituted P_3_TTM radical, we introduced methyl groups to the *ortho*-, *meta*-, and *para*-positions of methylphenyl (tolyl)-substituted 2-T_3_TTM, 3-T_3_TTM, and 4-T_3_TTM radicals, respectively. The position of the methyl groups was varied on the merit of controlling (1) π-conjugation between the phenyl rings and the TTM radical and (2) the inductive effect of the electron-donating methyl groups. Both factors are anticipated to influence the electron-donating strength of the phenyl rings and thus the CT contribution of electronic transitions in these radicals. Varied steric bulk between the phenyl rings and the TTM chlorophenyl rings would affect the molecular symmetry of the radicals in the ground and optically excited states. We extended the series through 2,4-, 2,5-, 2,6-, and 3,5-dimethylphenyl (xylyl)-substituted radicals 2,4-X_3_TTM, 2,5-X_3_TTM, 2,6-X_3_TTM, and 3,5-X_3_TTM, respectively, to 2,4,6-trimethylphenyl (mesityl)-substituted M_3_TTM. Finally, the steric bulk was increased with two isopropyl groups in the 2,6-ipP_3_TTM radical while still obtaining a reasonable yield in the 3-fold S–M coupling. Significantly lower yields were obtained with bulkier groups in the phenyl ring. In this way, a comprehensive, a new series of phenyl-substituted radical derivatives were synthesized for photophysical studies.

**Figure 2:**
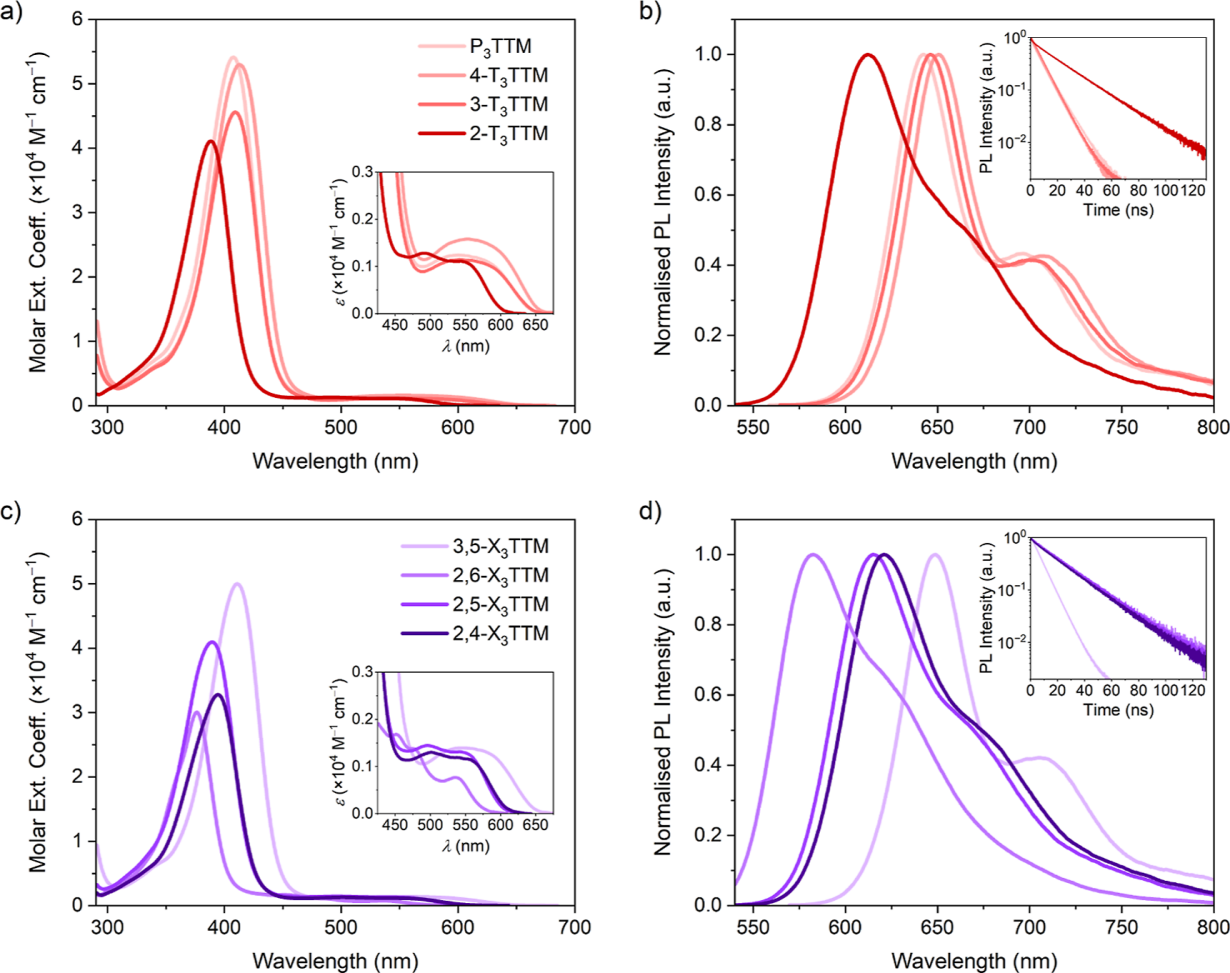
(a,c) Optical absorption and (b,d) photoluminescence spectra of radicals following 520 nm excitation in a 0.1 mM toluene solution. The insets in (a,c) show close-ups of the low-energy absorption region and the insets in (b,d) show total emission kinetics.

Figure 2 shows optical absorption and photoluminescence spectra of the synthesized radicals, and the photophysical parameters are summarized in Table 1. Excitation spectra and the corresponding spectra for M_3_TTM and 2,6-ipP_3_TTM are included in Figure S3. We observe that the absorption spectra are generally red-shifted with decreasing steric bulk due to effective conjugation between the phenyl rings and the radical center, whereas *para*-methyls serve to somewhat strengthen the lowest-energy D_0_–D_1_ transition suggesting stronger CT contribution in both tolyl- and xylyl-substituted systems. Emission of tolyl-substituted radicals (Figure 2b) is systematically red-shifted following the effects of conjugation and CT character in line with their absorption profiles. It is remarkable that phenyl substitution switches the dark TTM radical to bright with an increase of PLQE from 1% (for TTM) to 29% (for P_3_TTM) while red-shifting the emission from 568 to 643 nm in toluene solution, respectively.^23^ This is unexpected on the basis of molecular symmetry and lack of steric bulk, and reasons for the emission enhancement are discussed later in this article. We find a trend in increasing PLQE in the order 3-T_3_TTM < P_3_TTM < 4-T_3_TTM < 2-T_3_TTM, which stem from the nonradiative decay rates being suppressed in the same order and further from decreasing changes between ground- and excited-state conformations following our computational study (vide infra). Emission is enhanced with a *para*-methyl and even more so with an *ortho*-methyl coupled to the phenyl ring. 2-T_3_TTM stands out as the most emissive radical in this series and its PLQE of 65% at a peak wavelength of 612 nm is comparable to those of many state-of-the-art CT emitters that are based on nonalternant donor–acceptor-type radical design.^20,21,32,33^

Interplay between sterics and CT contribution becomes prominent in the emission of xylyl-substituted radicals (Figure 2d). Increased steric bulk in 2,6-X_3_TTM renders the radical poorly emissive, and this is further pronounced in 2,6-ipP_3_TTM. On the other hand, *meta*-methyls show minimal effect on emission wavelengths, kinetics, and PLQEs, as observed in comparison of 3-T_3_TTM and 3,5-X_3_TTM radicals. 2,4-X_3_TTM and 2,5-X_3_TTM can be described as intermediate structures where CT contribution is enhanced more by the *para*-methyls than by the *meta*-methyls, respectively, leading to stronger and red-shifted emission in the former case.

Emission in PMMA-doped films resembles that in solution, but we observe stronger vibronic features (Figure S4a,b). The peak wavelengths are red-shifted by less than 10 nm throughout the series confirming that emission in these radicals is dominated by changes in their substitution pattern, and also suggesting that symmetry can be broken in the solid state due to changes in conformation, as observed in structurally related triphenylphosphine radicals.^34^ In the crystalline solid state, emission is systematically quenched when going from most to least bulky substituents due to increasing aggregation in the same order (Figure S4c,d and Table S1). However, 2-T_3_TTM breaks this pattern and delivers the highest PLQE in this series, 25% at a peak wavelength of 706 nm, that is, for a neat radical that has not been diluted into any host matrix. Higher PLQEs have only been obtained from cocrystals of radicals doped in their corresponding αH precursors,^35,36^ whereas the red-shifted emission of neat 2-T_3_TTM can be ascribed to that of an excimer.^37,38^ These results indicate that ideal steric bulk maintains effective conjugation between the phenyl substituents and the radical center while still allowing symmetry breaking, as developed below.

**Figure 3:**
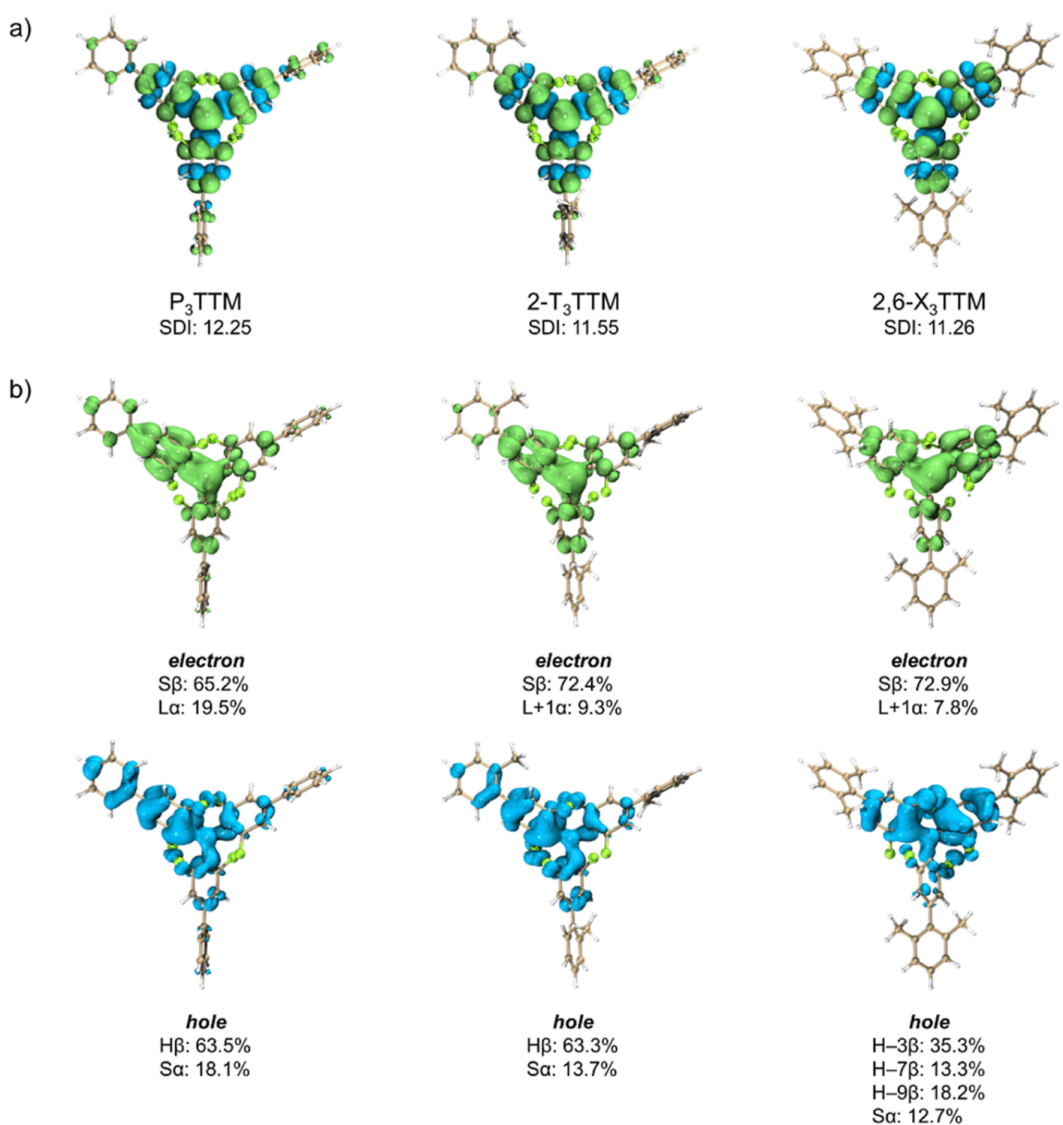
(a) Visualization of electron spin density for computationally optimized D_0_ state geometries of P_3_TTM, 2-T_3_TTM, and 2,6-X_3_TTM and (b) hole–electron analysis for vertical excited state based on their D_0_ geometries (S, SOMO; H, HOMO; L, LUMO).

**Figure 4:**
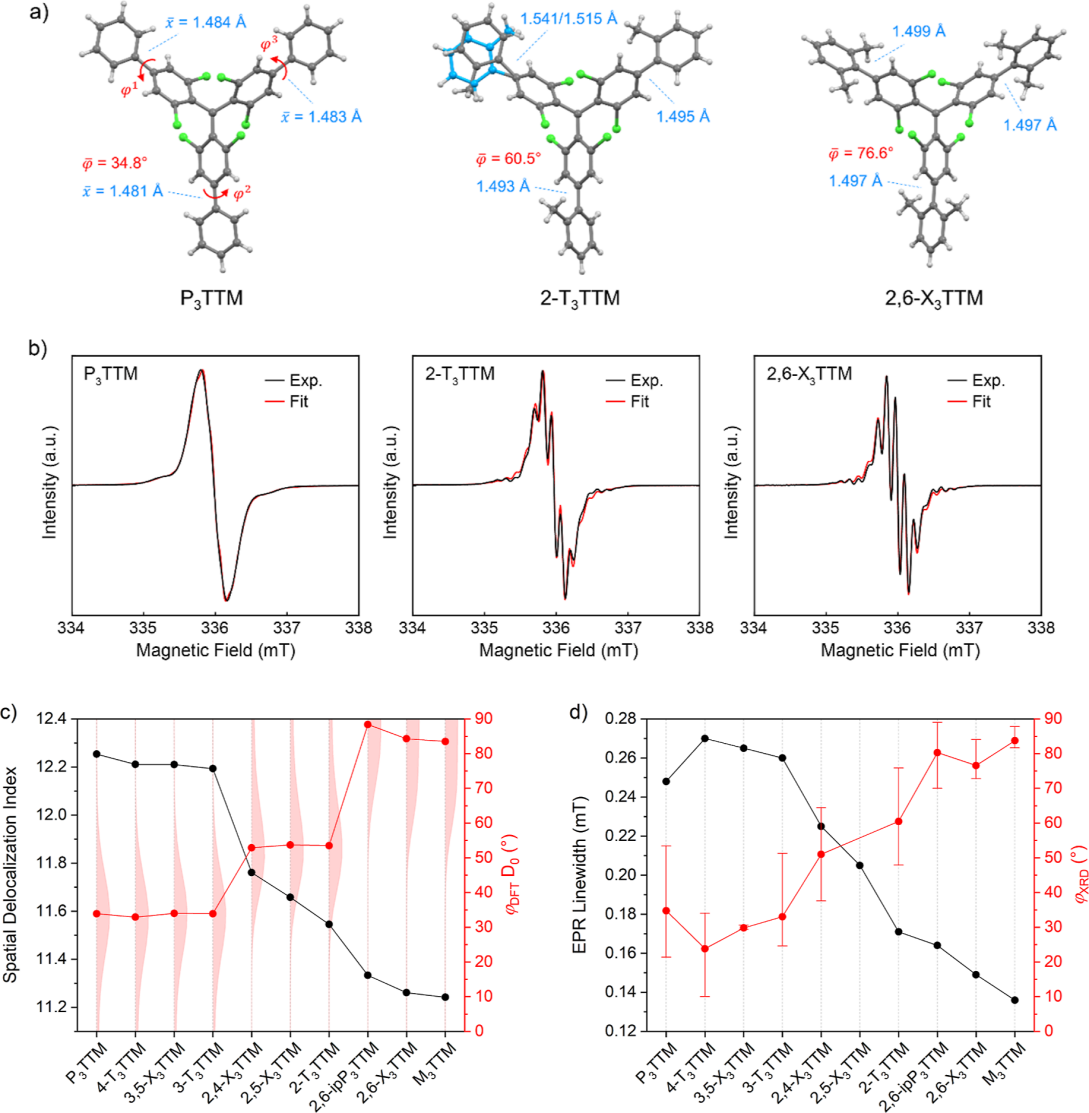
(a) Molecular structures from X-ray crystal structures of the synthesized P_3_TTM, 2-T_3_TTM, and 2,6-X_3_TTM radicals with average phenyl–phenyl dihedrals (red) and single bond lengths (blue). In the structure of 2-T_3_TTM, carbon atoms in blue highlight the 2-fold rotational disorder of the phenyl ring. (b) Continuous-wave X-band EPR spectra for the radicals measured at 200 K in a 1 mM toluene solution (black) and simulated spectra (red) with fit parameters given in Table S9. (c) Computational spatial delocalization index for electron spin density (black symbols) and dihedrals for aryl group Ar^1^ in different radical structures in their optimized D_0_ state (red symbols) with rotational freedom illustrated by Boltzmann distribution at 298.15 K (red filled curves, the thinnest part stands for minimum and the thickest part for maximum population). (d) Experimental peak-to-peak line width in the EPR spectra (black symbols) and average phenyl–phenyl dihedrals in the X-ray crystal structures (red symbols). The red intervals represent the variation between minimum and maximum torsion in these structures. A crystal structure could not be determined for 2,5-X_3_TTM (see Section S6).

Quantum-chemical modeling was employed to better understand the optical properties of the phenyl-substituted radicals. It is apparent from the geometry-optimized ground-state structures that positioning of the methyl groups leads to different in-phase and out-of-phase combinations of frontier molecular orbitals between the outer phenyl rings and the radical center (Section S4). Particularly, structures with the highest occupied molecular orbitals in-phase, e.g., P_3_TTM and 2-T_3_TTM, show higher oscillator strength for the lowest-energy D_0_–D_1_ excitation than the ones with the corresponding orbitals out-of-phase, e.g., 2,6-X_3_TTM (similar trend is observed experimentally in Figure 2a,c). In the former case, vertical excitation takes place primarily from the highest (doubly) occupied molecular orbital of the phenyl substituent to the singly occupied molecular orbital of the radical (HOMO–SOMO transition), whereas in the latter case, the transition is a result of a mixture of contributions from deeper-energy HOMOs around the radical center, as illustrated by the hole–electron analysis in Figure 3b. Moreover, isolation of the outer phenyl rings due to increasing steric bulk is observed as localization of electron spin density on the TTM core (Figure 3a), which is expressed numerically as a decreasing spatial delocalization index (SDI) in Figure 4c.

Our calculations suggest that both *ortho*- and *para*-methyls increase the oscillator strength of vertical and adiabatic D_1_ states relative to P_3_TTM due to enhanced phenyl-to-radical charge transfer (Table S4). This effect is cancelled out in 2,6-X_3_TTM as a result of steric bulk and poor orbital overlap, as discussed above. Generally, electronic transitions are localized between the radical center and one of the three outer phenyl rings. The corresponding phenyl–phenyl arm becomes coplanar and the sp^2^–sp^2^ single bond shortens in the adiabatic D_1_ state, suggesting that excited-state symmetry breaking operates in all 3-fold aryl-substituted radicals regardless of their steric bulk (Table S3). This has not been observed previously in trityl radicals. Structures without *ortho*-methyls are nearly coplanar already in the ground state, whereas those with two *ortho*-methyls remain twisted also in the D_1_ state and their phenyl–phenyl bond lengths remain the most single bond-like in this series. The experimental photoluminescence spectra are more structured in the former case (Figure 2b,d). 2-T_3_TTM, 2,4-X_3_TTM, and 2,5-X_3_TTM with one *ortho*-methyl stand in the middle, resulting in smallest change between ground- and excited-state dihedrals, 15.8, 17.3, and 17.2°, respectively, and highest oscillator strength for the adiabatic D_1_ state, which in part explain the low experimental nonradiative decay rates in these three systems. We also find that *ortho*-methyls provide significant energy barrier for planarization minimizing chances for rotation past coplanar conformation (Figure S5). Population of different conformations within these limits is illustrated by Boltzmann distribution for each structure in Figure 4c, where *ortho*-methyls control rotational freedom and thereby effective conjugation between the phenyl ring and the radical center.

Molecular structures from X-ray crystallography show further evidence of changes in conformation in the synthesized radicals (Figure 4a,d). For molecules without *ortho*-methyls, a wide range of dihedrals are observed between 10.0 and 53.4°, whereas for those with one *ortho*-methyl, the range is shifted upward between 37.6 and 75.9°. In molecules with two *ortho*-methyls, the distribution is narrowed near orthogonal between 70.0 and 89.0°. These experimental observations fit well into the population distributions of the computational models shown in Figure 4c. Crystal structures and average phenyl–phenyl dihedrals and bond lengths for P_3_TTM, 2-T_3_TTM, and 2,6-X_3_TTM are included in Figure 4a, which shows apparent disorder of the orientation of one of the phenyl rings in 2-T_3_TTM (see Section S6 for further discussion). Simulation of these structures suggests that electronic transitions in a monomolecular level resemble those of our computational models (Table S5), further indicating that the observed red-shifted emission in the crystalline solid state comes from an excimer (Figure S4 and Table S1).

Electron paramagnetic resonance (EPR) spectroscopy provides valuable insight into the environment of the unpaired electron in the synthesized molecules. Figure 4b shows experimental spectra for P_3_TTM, 2-T_3_TTM, and 2,6-X_3_TTM recorded in a 1 mM toluene solution at 200 K. The distinct spectral feature observed in these compounds is EPR peak-to-peak line width. The broad septet band in 2-T_3_TTM and the narrow septet splitting in 2,6-X_3_TTM are due to isotropic hyperfine coupling between the radical electron and the closest six aromatic hydrogens in the TTM structure (labeled δ/δ′ hydrogens in Table S9). In the case of P_3_TTM, the septet band appears considerably broader and less resolved. Upon analyzing the EPR spectra for all radicals in this study (Figures 4b and S7), we find that the *g*-factor remains consistent (*g* = 2.0156), and there are slight variations in the hyperfine coupling constants with the aromatic hydrogens. Since the similarity in these parameters does not account for the differences observed in peak line widths, we hypothesize the peak line widths are influenced by molecular motion, and the dynamic averaging resulting from conformational flexibility may lead to narrower peak widths.^39^ To quantify this, we have extracted peak-to-peak line widths through fitting,^40,41^ and the parameters are summarized in Table S9. Figure 4d illustrates a systematic decrease in line width as the steric bulk increases around the TTM core, indicating more conformational flexibility as the radical becomes less conjugated, in good agreement with the computational spin delocalization shrinking in the same order (Figure 4c). Overall, fine changes in steric bulk and electronic coupling of the phenyl rings are observed as greatly enhanced luminescence in 2-T_3_TTM, as indicated by both computational and experimental analyses.

## Conclusions

In conclusion, sterics play various roles in the emission of the TTM radical. We show that excited-state symmetry breaking is a general phenomenon that operates in 3-fold aryl-substituted radicals and that luminescence can be boosted by controlling the steric bulk in these structures. Sterics also dictate the inductive effects of the aryl substituents. Specifically, *ortho*-methylphenyl-substituted 2-T_3_TTM stands as a prime example where the phenyl ring is coupled in-phase to the radical and symmetry breaking is achieved with minimal change in excited-state conformation, resulting in suppression of nonradiative decay channels, and thus, a photoluminescence quantum efficiency of 65% is achieved at a peak wavelength of 612 nm in solution. The same design motif holds in the solid state, and neat crystals of 2-T_3_TTM maintain a photoluminescence quantum efficiency of 25% at a peak wavelength of 706 nm. This study demonstrates that steric control of electronic coupling in aryl-substituted radicals can be used as an effective method to design highly luminescent spin systems based on alternant symmetry structures and, by analogy, nonalternant hydrocarbon structures. The relevant question is from which position these substituents are coupled to the radical.

## Experimental Methods

### Characterization and Techniques

NMR spectra were recorded on a 400 MHz Bruker Avance III HD spectrometer (^1^H, 400 MHz; ^13^C, 100 MHz). Chemical shifts are reported in δ (ppm) relative to the solvent peak: chloroform-*d* (CDCl_3_: ^1^H, 7.26 ppm; ^13^C, 77.16 ppm) and dichloromethane-*d*_2_ (CD_2_Cl_2_: ^1^H, 5.32 ppm; ^13^C, 53.84 ppm). Mass spectra were obtained on a Waters Xevo G2-S benchtop QTOF mass spectrometer using electrospray ionization (ESI) or an atmospheric solid analysis probe (ASAP). C, H, and N combustion elemental analyses were obtained on an Exeter Analytical Inc. CE-440 elemental analyzer, and the results are reported as an average of two samples. Flash chromatography was carried out using Biotage Isolera Four System and Biotage SNAP/Sfär Silica flash cartridges.

### Optical Absorption and Photoluminescence Spectroscopy

UV–visible spectra were measured with a commercially available Shimadzu UV-1800 spectrophotometer. Steady-state photoluminescence and excitation spectra of samples in solution were measured with a commercially available Edinburgh Instruments FS5 Spectrofluorometer system using a xenon lamp as the light source. Photoluminescence of crystalline solid-state samples was measured with the same instrument, and PLQEs were obtained using an integrating sphere and an excitation wavelength of 405 nm. The measurements were carried out by placing vacuum-dried single crystals between two glass plates (preparation of the crystals is described in Experimental Section for X-ray crystallography). Photoluminescence of samples in PMMA films was measured in a home-built setup by providing a continuous wave excitation at 405 nm using a diode laser. Photoluminescence was collected in a reflection mode setup after passing photons through a 450 nm long-pass filter (Thor Laboratories). Transmitted photons were collected in a collimating 2-lens apparatus and directed into an optical fiber which supplied the photons into a calibrated grating spectrometer (Andor SR-303i) and finally into a Si-camera where they were recorded. Output spectra were corrected by taking into account the filter transmission and camera sensitivity. PLQE measurements of samples in solution and PMMA films were performed using an integrating sphere, and samples were excited by a continuous-wave 405 nm laser. The laser and sample emission signals were measured by a calibrated grating spectrometer (Andor SR-303i) using a Si detector. Time-resolved single photon counting using timing electronics (TimeHarp260) was carried out by irradiating the samples with an electrically pulsed 407 nm laser using a function generator at a frequency of 5–10 MHz providing a time resolution of up to 200 ns. Photons emitted from the sample were passed through a 450 nm long-pass filter (Thor Laboratories) to remove laser scatter. Subsequently transmitted photons were collected by a Si-based single-photon avalanche photodiode. All spectroscopy was carried out under ambient air.

### Cyclic Voltammetry

Cyclic voltammetry was carried out on a PalmSens EmStat4S potentiostat in a three-electrode setup using a glassy carbon (GC) electrode (3.0 mm diameter) as the working electrode (WE), platinum wire as the counter electrode (CE), and freshly activated silver wire as the Ag/Ag^+^ reference electrode (RE). The silver wire was activated by immersion in concentrated HCl solution to remove any silver oxides or other impurities and then rinsed with water and acetone and dried prior to each measurement. The RE was calibrated against ferrocene/ferrocenium (Fc/Fc^+^) redox couple at the end of each measurement (the Fc/Fc^+^ half-wave potential, *E*_1/2_, was determined at 0.20 V vs Ag/Ag^+^). The supporting electrolyte was 0.1 M solution of Bu_4_NPF_6_ in anhydrous THF, and the scan rate was 0.1 V s^–1^. The electrolyte was bubbled with Ar gas before each measurement to remove any dissolved oxygen. Sample concentration was on the order of 10^–5^ M.

### Density Functional Theory Calculations

Density functional theory (DFT) calculations were performed using the Gaussian 16 program. Ground-state geometries were optimized at an unrestricted UB3LYP/def2-SVP level (for radicals) or at a restricted B3LYP/def2-SVP level (for aryl groups). Dispersion correction was conducted by Grimme’s D3 version.^42^ Potential energy surfaces were scanned as a function of dihedral for aryl group Ar^1^ at steps of 5° by optimizing the structure to the energy minimum at each step and using the same unrestricted functional and basis set as above. Boltzmann distributions were calculated from these potential energy scans at 298.15 K. Based on the optimized ground-state geometries, vertical excitation energies were evaluated at UPBE0/def2-TZVP by time-dependent DFT (TD-DFT) treatment. The D_1_ state geometries were optimized at the UPBE0/def2-SVP level, and the adiabatic D_1_ state energies were evaluated using the same functional but basis set of def2-TZVP. For comparison, excited-state analysis was also carried out with the UM06-2X functional^43^ while using the same basis sets at each step as described for the UPBE0 functional. The numerical data in Table S4 show that both methods give similar trends that follow the experimental observations but with a difference that UPBE0 better reproduces the experimental energies and oscillator strengths, and it is therefore selected as the representative method in this study. Following these results, excited-state analysis of molecular structures from X-ray crystallography was carried out in two steps: first, all hydrogen atom positions were optimized at the UB3LYP/def2-SVP level by freezing all carbon atoms to the X-ray crystal structure geometry and, second, vertical excitation energies of these structures were calculated by TD-DFT at the UPBE0/def2-TZVP level. Spatial delocalization indexes and excited-state analyses were processed using the Multiwfn 3.8 program according to the program manual and literature method.^44^ Visualization of the optimized structures was done using the Visual Molecular Dynamics 1.9.3 (VMD) software.^45^

### EPR Spectroscopy

EPR experiments were conducted by using an X-band benchtop EPR spectrometer (E5000, Magnettech), operating at a microwave frequency of 9.47 GHz and a temperature of 200 K, regulated by a variable-temperature unit. A modulation field of 0.02 mT was applied at a modulation frequency of 100 kHz with a microwave power of 5 mW. For sample preparation, all samples were dissolved in toluene at a concentration of 1 mM and then transferred into glass capillaries with a 1 mm diameter (Bruker), which were subsequently sealed with Critoseal. EasySpin software was employed to simulate the EPR spectra.^46^

### X-ray Crystallography

Crystals were prepared by dissolving the sample in DCM in an NMR tube, and either MeOH or EtOH was added on top as an antisolvent, and the solvents were allowed to mix slowly in the dark. Single-crystal X-ray diffraction data were collected on a Bruker D8-QUEST diffractometer, equipped with an Incoatec IμS Cu microsource (λ = 1.5418 Å) and a PHOTON-III detector operating in shutterless mode. The temperature was controlled at 180(2) K using an Oxford Cryosystems open-flow N_2_ Cryostream. The control and processing software was a Bruker *APEX4*. The diffraction images were integrated using *SAINT* in *APEX4*, and a multiscan correction was applied using *SADABS*. The final unit–cell parameters were refined against all of the reflections over the full data range. Structures were solved using *SHELXT*(47) and refined using *SHELXL*.^48^ Full details of the crystallographic refinements are provided in Supporting Information. The crystal structures were visualized using the Mercury 2021.3.0 software.^49,50^

## Data Availability

Optical absorption and photoluminescence spectra, emission kinetics, computational atomic coordinates of optimized ground- and excited-state structures, and EPR spectra are openly available in the University of Cambridge Repository at 10.17863/CAM.107368. All other data underlying this study are available in the published article and its Supporting Information.

## Supporting Information Available

Supporting figures, synthetic procedures and structural characterization, additional optical spectra, computational structures and data, cyclic voltammetry, X-ray crystallographic structures, refinements and data, EPR spectra and data, and NMR spectra (PDF)
