# Supplementary material for: Steric Control of Luminescence in Phenyl-Substituted Trityl Radicals
Source: J Am Chem Soc. 2024 May 2;146(19):13133–41. doi: 10.1021/jacs.4c00292 (PMC11099960; doi:10.1021/jacs.4c00292)
Supplement: Supplementary file 1 — ja4c00292_si_001.pdf [file ja4c00292_si_001.pdf]

SUPPORTING INFORMATION

Steric Control of Luminescence in Phenyl Substituted Trityl Radicals

Petri Murto,[a,b] Biwen Li,[b] Yao Fu,[a] Lucy E. Walker,[a,b] Laura Brown,[a] Andrew D. Bond,[a] Weixuan Zeng,[a] Rituparno Chowdhury,[b] Hwan-Hee Cho,[b] Craig P. Yu,[a,b] Clare P. Grey,[a] Richard H. Friend,[b,*] and Hugo Bronstein[a,b,*]

[a] *Yusuf Hamied Department of Chemistry, University of Cambridge, Cambridge, CB2 1EW, United Kingdom*

[b] *Cavendish Laboratory, University of Cambridge, Cambridge, CB3 0HE, United Kingdom*

*Email: hab60@cam.ac.uk; rhf10@cam.ac.uk

Table of Contents

S1. Supporting Figures S3

S2. Materials and Synthesis S5

S3. Optical Spectroscopy S14

S4. DFT Calculations S18

S5. Cyclic Voltammetry S52

S6. X-Ray Crystallography S54

S7. EPR Spectroscopy S62

S8. NMR Spectra S64

Supporting References S85

S1. Supporting Figures

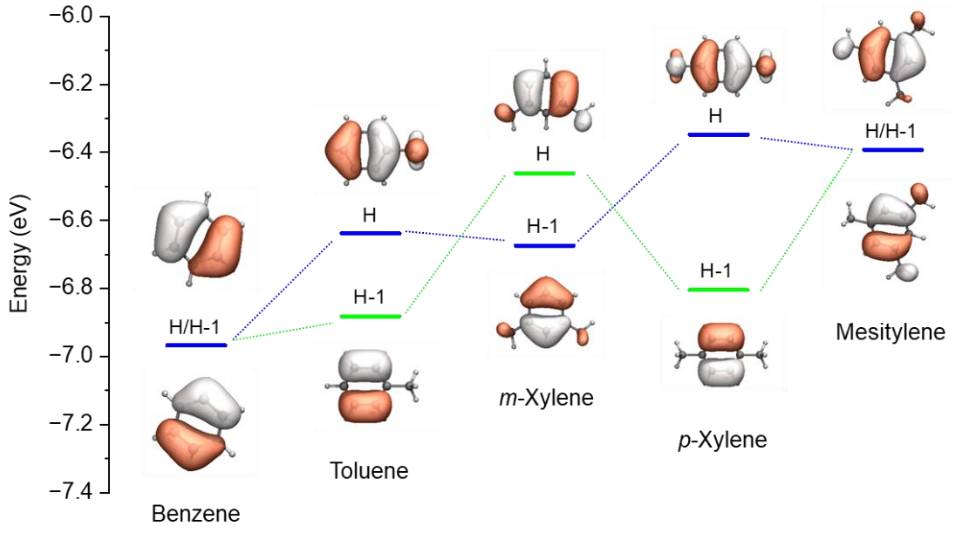

Figure S1. Calculated frontier molecular orbital energy levels of aryl groups used in this study. Green and blue colours stand for orbitals with and without nodes on aromatic carbon atoms, respectively.

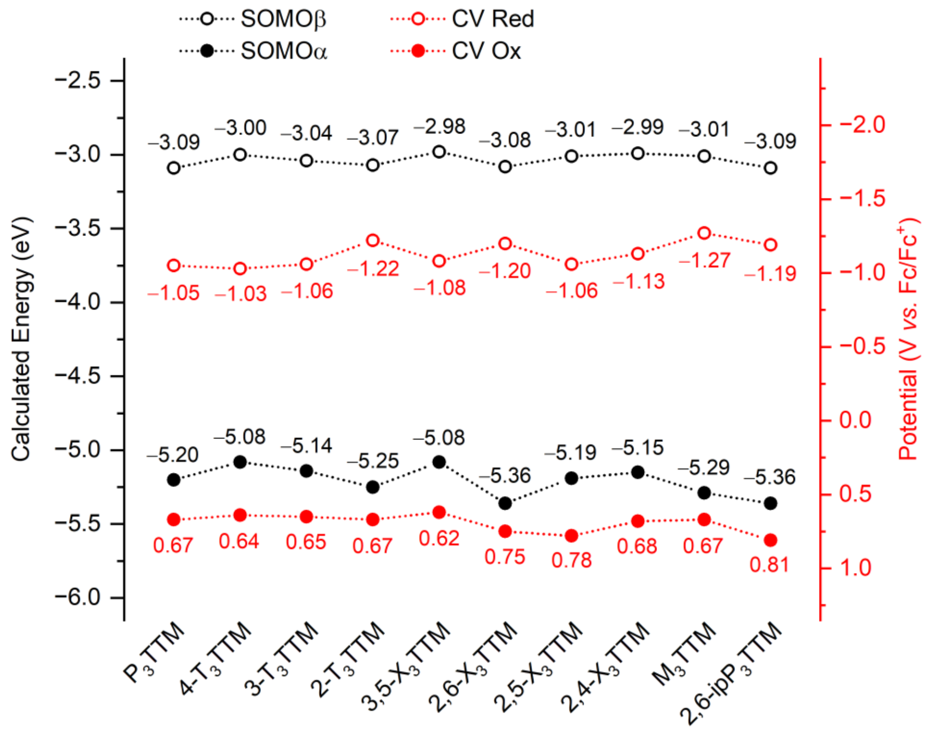

Figure S2. Computational SOMO energy levels (black) and electrochemical redox potentials (red) for the phenyl substituted trityl radicals.

Scheme S1. Chemical structures of the synthesized radicals

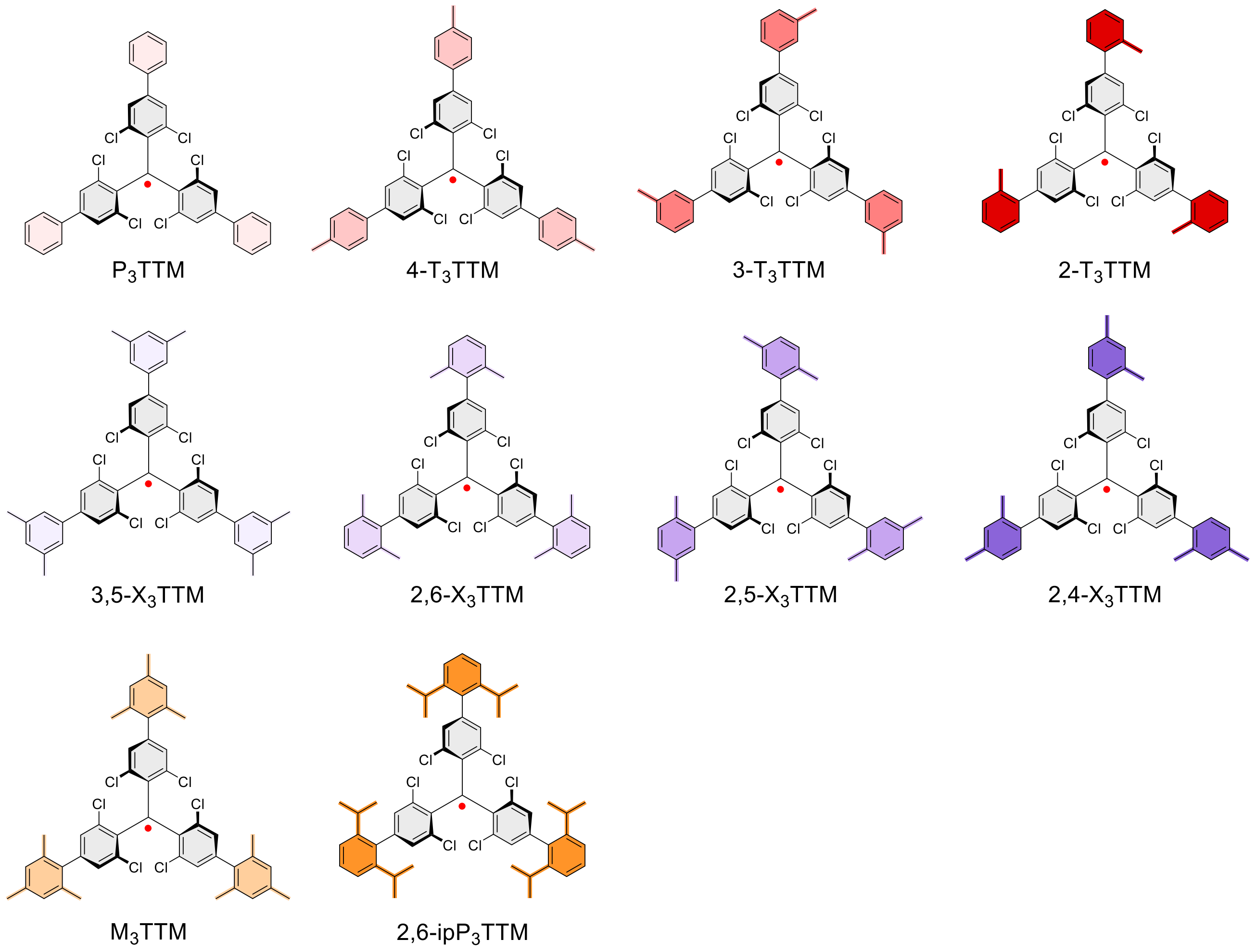

S2. Materials and Synthesis

Tris(2,4,6-trichlorophenyl)methane (αHTTM) was synthesized following previous procedures from us[1] and others,[2,3] here at a scale of 21.6 g (70%). Other reagents, catalysts and (anhydrous) solvents were purchased from Merck, Fluorochem, Alfa Aesar and Acros Organics and used as received.

General procedure for the synthesis of αH precursors. αHTTM (1 equiv.), $ArB(OH)_2$ (6 equiv.), $Pd(OAc)_2$ (0.02 equiv.), SPhos (0.04 equiv.) and K_3PO_4 (9.6 equiv.) were added into a microwave vial and the vial was subjected to three vacuum/Ar gas refill cycles. Anhydrous 1,4-dioxane was added (10 mL/mmol of αHTTM). The mixture was bubbled with Ar gas for 15 min and then heated at 80 °C in an oil bath for 24 h. After cooling to room temperature, the mixture was diluted with hexane and extracted three times with water. The organic phase was dried over anhydrous $MgSO_4$. Solvent was removed and the crude product was purified with column chromatography over silica gel by gradually increasing the eluent polarity from hexane to 5–10% (v/v) DCM in hexane. The product was recrystallized from DCM/EtOH. Finally, the solvent was removed and the solids were dried *in vacuo*. Details of individual reactions and deviations from this procedure are provided below.

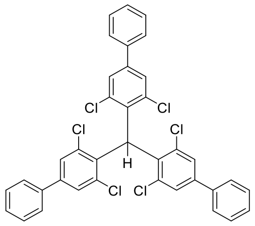

αHP3TTM

Tris(3,5-dichloro-[1,1'-biphenyl]-4-yl)methane (αHP3TTM). αHTTM (1.127 g, 2.03 mmol, 1 equiv.), phenylboronic acid (1.983 g, 16.26 mmol, 8 equiv.), $Pd(OAc)_2$ (0.0091 g, 0.041 mmol, 0.02 equiv.), SPhos (0.0334 g, 0.081 mmol, 0.04 equiv.) and K_3PO_4 (4.143 g, 19.52 mmol, 9.6 equiv.). The product was collected as white solid (0.498 g, 36%). A larger scale reaction yielded 0.941 g (38%). ^1H NMR (400 MHz, $CDCl_3$) δ 7.65–7.57 (m, 9H), 7.51–7.42 (m, 9H), 7.42–7.35 (m, 3H), 6.96 (s, 1H).

^{13}C NMR (100 MHz, $CDCl_3$) δ 141.74, 138.04, 138.00, 137.31, 134.63, 129.14, 128.59, 128.53, 127.01, 126.86, 50.62. TOF-MS-ASAP$^+$ Calcd. for $[C_{37}H_{22}Cl_6]^+$: 675.9853. Found: *m/z* = 675.9825.

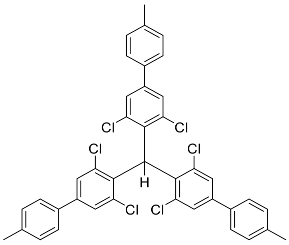

αH4-T3TTM

Tris(3,5-dichloro-4'-methyl-[1,1'-biphenyl]-4-yl)methane (αH4-T$_3$TTM). αHTTM (0.100 g, 0.18 mmol, 1 equiv.), *p*-tolylboronic acid (0.147 g, 1.08 mmol, 6 equiv.), $Pd(OAc)_2$ (0.0008 g, 0.004 mmol, 0.02 equiv.), SPhos (0.0030 g, 0.007 mmol, 0.04 equiv.) and K_3PO_4 (0.368 g, 1.73 mmol, 9.6 equiv.). The product was collected as white solid (0.050 g, 38%). A larger scale reaction yielded 0.162 g (25%). ^1H NMR (400 MHz, CD_2Cl_2) δ 7.63 (d, J = 2.0 Hz, 3H), 7.53–7.49 (m, 9H), 7.30–7.26 (m, 6H), 6.94 (s, 1H), 2.40 (s, 9H). ^{13}C NMR (100 MHz, CD_2Cl_2) δ 142.03, 139.03, 138.20, 137.45, 135.15, 134.56, 130.13, 128.56, 126.97, 126.82, 50.87, 21.28. TOF-MS-ASAP$^+$ Calcd. for $[C_{40}H_{28}Cl_6]^{H+}$: 719.0400. Found: *m/z* = 719.0374.

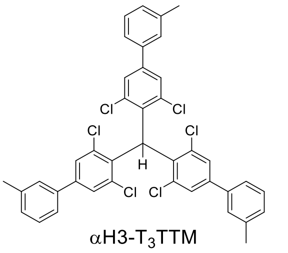

αH3-T3TTM

Tris(3,5-dichloro-3'-methyl-[1,1'-biphenyl]-4-yl)methane (αH3-T$_3$TTM). αHTTM (0.500 g, 0.90 mmol, 1 equiv.), *m*-tolylboronic acid (0.736 g, 5.41 mmol, 6 equiv.), $Pd(OAc)_2$ (0.0041 g, 0.018 mmol, 0.02 equiv.), SPhos (0.0148 g, 0.036 mmol, 0.04 equiv.) and K_3PO_4 (1.838 g, 8.66 mmol, 9.6 equiv.). The product was collected as white solid (0.466 g, 72%). ^1H NMR (400 MHz, $CDCl_3$) δ 7.60 (d, J = 2.0 Hz, 3H), 7.46 (d, J = 2.0 Hz, 3H), 7.43–7.37 (m, 6H), 7.34 (t, J = 7.5 Hz, 3H), 7.20 (d, J = 7.4 Hz, 3H), 6.94 (s, 1H), 2.42 (s, 9H). ^{13}C NMR (100 MHz, $CDCl_3$) δ 141.86, 138.80, 137.99, 137.96, 137.24,

134.53, 129.25, 129.03, 128.57, 127.78, 126.85, 124.10, 50.61, 21.64. TOF-MS-ASAP$^+$ Calcd. for $[C_{40}H_{28}Cl_6]^{H+}$: 719.0400. Found: *m/z* = 719.0426.

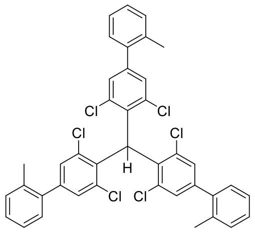

αH2-T$_3$TTM

Tris(3,5-dichloro-2'-methyl-[1,1'-biphenyl]-4-yl)methane (αH2-T$_3$TTM). αHTTM (0.500 g, 0.90 mmol, 1 equiv.), *o*-tolylboronic acid (0.736 g, 5.41 mmol, 6 equiv.), $Pd(OAc)_2$ (0.0041 g, 0.018 mmol, 0.02 equiv.), SPhos (0.0148 g, 0.036 mmol, 0.04 equiv.) and K_3PO_4 (1.838 g, 8.66 mmol, 9.6 equiv.). The product was collected as white solid (0.477 g, 73%). A smaller scale reaction yielded 0.303 g (78%). ^{1}H NMR (400 MHz, CD_2Cl_2) δ 7.37 (d, J = 1.8 Hz, 3H), 7.31–7.24 (m, 12H), 7.23 (d, J = 1.8 Hz, 3H), 7.02 (s, 1H), 2.29 (s, 9H). ^{13}C NMR (100 MHz, CD_2Cl_2) δ 143.23, 139.25, 137.54, 136.81, 135.77, 134.61, 131.27, 130.88, 129.73, 129.49, 128.54, 126.34, 50.92, 20.48. TOF-MS-ESI$^+$ Calcd. for $[C_{40}H_{28}Cl_6]^+$: 718.0322. Found: *m/z* = 718.0327.

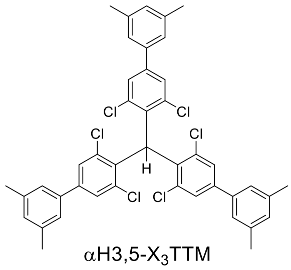

αH3,5-X$_3$TTM

Tris(3,5-dichloro-3',5'-dimethyl-[1,1'-biphenyl]-4-yl)methane (αH3,5-X$_3$TTM). αHTTM (0.300 g, 0.54 mmol, 1 equiv.), (3,5-dimethylphenyl)boronic acid (0.487 g, 3.25 mmol, 6 equiv.), $Pd(OAc)_2$ (0.0024 g, 0.011 mmol, 0.02 equiv.), SPhos (0.0089 g, 0.022 mmol, 0.04 equiv.) and K_3PO_4 (1.103 g, 5.20 mmol, 9.6 equiv.). The product was collected as white solid (0.232 g, 59%). ^{1}H NMR (400 MHz, $CDCl_3$) δ 7.59 (d, J = 1.9 Hz, 3H), 7.45 (d, J = 1.9 Hz, 3H), 7.21 (d, J = 1.6 Hz, 6H), 7.02 (s, 3H), 6.92 (s, 1H), 2.38 (s, 18H). ^{13}C NMR (100 MHz, $CDCl_3$) δ 141.95, 138.68, 138.00, 137.90, 137.17, 134.45,

130.11, 128.56, 126.84, 124.91, 50.59, 21.51. TOF-MS-ASAP$^+$ Calcd. for $[C_{43}H_{34}Cl_6]^{H+}$: 761.0870. Found: *m/z* = 761.0839.

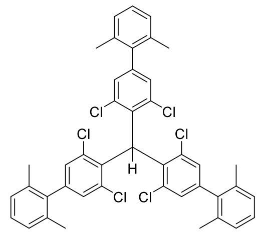

αH2,6-X$_3$TTM

Tris(3,5-dichloro-2',6'-dimethyl-[1,1'-biphenyl]-4-yl)methane (αH2,6-X$_3$TTM). αHTTM (0.500 g, 0.90 mmol, 1 equiv.), (2,6-dimethylphenyl)boronic acid (0.812 g, 5.41 mmol, 6 equiv.), $Pd(OAc)_2$ (0.0041 g, 0.018 mmol, 0.02 equiv.), SPhos (0.0148 g, 0.036 mmol, 0.04 equiv.) and K_3PO_4 (1.838 g, 8.66 mmol, 9.6 equiv.). The product was collected as white solid (0.446 g, 65%). A smaller scale reaction yielded 0.276 g (67%). ^{1}H NMR (400 MHz, $CDCl_3$) δ 7.22–7.18 (m, 5H), 7.17 (s, 1H), 7.13 (s, 4H), 7.11 (s, 2H), 7.06 (d, J = 1.8 Hz, 3H), 7.03 (s, 1H), 2.10 (d, J = 1.3 Hz, 18H). ^{13}C NMR (100 MHz, $CDCl_3$) δ 142.06, 138.97, 137.73, 137.06, 135.99, 135.94, 134.44, 130.95, 129.20, 127.91, 127.56, 127.54, 50.72, 20.86, 20.84. TOF-MS-ESI$^+$ Calcd. for $[C_{43}H_{34}Cl_6]^{H+}$: 761.0870. Found: *m/z* = 761.0853.

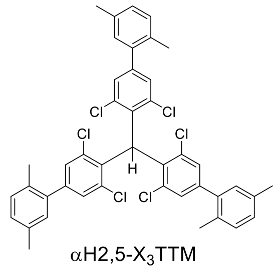

αH2,5-X$_3$TTM

Tris(3,5-dichloro-2',5'-dimethyl-[1,1'-biphenyl]-4-yl)methane (αH2,5-X$_3$TTM). αHTTM (0.300 g, 0.54 mmol, 1 equiv.), (2,5-dimethylphenyl)boronic acid (0.487 g, 3.25 mmol, 6 equiv.), $Pd(OAc)_2$ (0.0024 g, 0.011 mmol, 0.02 equiv.), SPhos (0.0089 g, 0.022 mmol, 0.04 equiv.) and K_3PO_4 (1.103 g, 5.20 mmol, 9.6 equiv.). The product was collected as white solid (0.346 g, 89%). ^{1}H NMR (400 MHz, $CDCl_3$) δ 7.33 (d, J = 1.8 Hz, 3H), 7.19 (d, J = 1.8 Hz, 3H), 7.15 (d, J = 7.6 Hz, 3H), 7.11–7.06 (m,

6H), 6.99 (s, 1H), 2.35 (s, 9H), 2.25 (s, 9H). ^{13}C NMR (100 MHz, $CDCl_3$) δ 142.92, 138.85, 137.27, 136.55, 135.57, 134.30, 132.24, 130.90, 130.55, 130.25, 129.14, 128.93, 50.59, 20.99, 20.02. TOF-MS-ASAP$^+$ Calcd. for $[C_{43}H_{34}Cl_6]^{H+}$: 761.0870. Found: *m/z* = 761.0896.

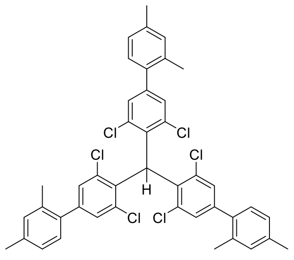

αH2,4-X_3TTM

Tris(3,5-dichloro-2',4'-dimethyl-[1,1'-biphenyl]-4-yl)methane (αH2,4-X_3TTM). αHTTM (0.300 g, 0.54 mmol, 1 equiv.), (2,4-dimethylphenyl)boronic acid (0.487 g, 3.25 mmol, 6 equiv.), $Pd(OAc)_2$ (0.0024 g, 0.011 mmol, 0.02 equiv.), SPhos (0.0089 g, 0.022 mmol, 0.04 equiv.) and K_3PO_4 (1.103 g, 5.20 mmol, 9.6 equiv.). The product was collected as white solid (0.234 g, 57%). ^{1}H NMR (400 MHz, $CDCl_3$) δ 7.31 (d, J = 1.8 Hz, 3H), 7.18 (d, J = 1.8 Hz, 3H), 7.14 (d, J = 7.6 Hz, 3H), 7.10–7.03 (m, 6H), 6.99 (s, 1H), 2.36 (s, 9H), 2.26 (s, 9H). ^{13}C NMR (100 MHz, $CDCl_3$) δ 142.76, 137.97, 137.24, 136.52, 136.21, 135.20, 134.20, 131.38, 131.00, 129.49, 129.22, 126.78, 50.57, 21.24, 20.45. TOF-MS-ASAP$^+$ Calcd. for $[C_{43}H_{34}Cl_6]^{H+}$: 761.0870. Found: *m/z* = 761.0860.

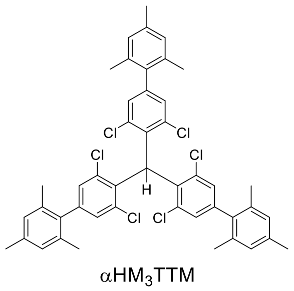

αHM_3TTM

Tris(3,5-dichloro-2',4',6'-trimethyl-[1,1'-biphenyl]-4-yl)methane (αHM_3TTM). αHTTM (10.00 g, 18.0 mmol, 1 equiv.), mesitylboronic acid (10.36 g, 63.1 mmol, 3.5 equiv.), $Pd(OAc)_2$ (0.0810 g, 0.361 mmol, 0.02 equiv.), SPhos (0.2962 g, 0.722 mmol, 0.04 equiv.) and K_3PO_4 (21.44 g, 101.0 mmol, 5.6 equiv.). Reaction was run in a two-neck round-bottom flask equipped with a condenser. The product

was collected as white solid (2.19 g, 15%). ^{1}H NMR (400 MHz, $CDCl_3$) δ 7.18 (d, J = 1.8 Hz, 3H), 7.04 (d, J = 1.8 Hz, 3H), 7.01 (s, 1H), 6.94 (s, 6H), 2.33 (s, 9H), 2.06 (d, J = 1.7 Hz, 18H). ^{13}C NMR (100 MHz, $CDCl_3$) δ 142.12, 137.64, 137.52, 136.96, 136.21, 135.88, 135.82, 134.35, 131.20, 129.44, 128.31, 128.28, 50.70, 21.20, 20.75, 20.73. TOF-MS-ASAP$^+$ Calcd. for $[C_{46}H_{40}Cl_6]^+$: 802.1261. Found: m/z = 802.1235.

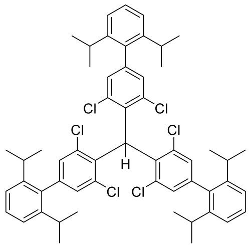

αH2,6-ipP$_3$TTM

Tris(3,5-dichloro-2',6'-diisopropyl-[1,1'-biphenyl]-4-yl)methane (αH2,6-ipP$_3$TTM). αHTTM (0.250 g, 0.45 mmol, 1 equiv.), (2,6-diisopropylphenyl)boronic acid (0.558 g, 2.71 mmol, 6 equiv.), $Pd(OAc)_2$ (0.0020 g, 0.009 mmol, 0.02 equiv.), SPhos (0.0074 g, 0.018 mmol, 0.04 equiv.) and K_3PO_4 (0.919 g, 4.33 mmol, 9.6 equiv.). The product was collected as white solid (0.064 g, 15%). ^{1}H NMR (400 MHz, $CDCl_3$) δ 7.37 (t, J = 7.8 Hz, 3H), 7.25–7.19 (m, 9H), 7.10 (d, J = 1.8 Hz, 3H), 7.07 (s, 1H), 2.77–2.64 (m, 6H), 1.18–1.06 (m, 36H). ^{13}C NMR (100 MHz, $CDCl_3$) δ 146.80, 146.64, 141.57, 137.21, 136.74, 136.68, 134.34, 131.59, 129.67, 128.69, 122.85, 122.82, 50.75, 30.56, 30.42, 24.39, 24.31, 24.29, 24.04. TOF-MS-ESI$^+$ Calcd. for $[C_{55}H_{58}Cl_6]^+$: 928.2670. Found: m/z = 928.2656.

General procedure for the synthesis of π-radicals. Following our previously reported procedure,[1] αH precursor (1 equiv.) was added into a microwave vial and the vial was subjected to three vacuum/Ar gas refill cycles. Anhydrous THF was added to dissolve all starting material followed by addition of anhydrous DMSO in 1:3 (v/v) THF/DMSO ratio making a 0.5 wt% solution of the αH precursor. The mixture was bubbled with Ar gas for 15 min and covered from light. In the darkness, 40% Bu_4NOH (aq) (2 equiv.) (bubbled with Ar gas for 15 min prior to use) was added and the mixture was stirred at

room temperature for 6 h. *p*-Chloranil (2.5 equiv.) was added, and the mixture was stirred another 1 h. The mixture was diluted with hexane, extracted three times with water and the organic phase was dried over anhydrous $MgSO_4$. Solvent was removed and the crude product was purified with column chromatography over silica gel by gradually increasing the eluent polarity from hexane to 5–10% (v/v) DCM in hexane. The product was recrystallized from DCM/EtOH. Finally, the solvent was removed and the solids were dried *in vacuo*. All radicals were stable under ambient air in both solution and solid state, but they were stored under inert gas in the dark as a standard procedure. Details of individual reactions and deviations from this procedure are provided below.

Tris(3,5-dichloro-[1,1'-biphenyl]-4-yl)methyl radical (P_3TTM). αHP_3TTM (0.230 g, 0.34 mmol, 1 equiv.), 40% Bu_4NOH (aq) (0.45 mL, 0.68 mmol, 2 equiv.), *p*-chloranil (0.208 g, 0.85 mmol, 2.5 equiv.). The product was collected as red solid (0.171 g, 74%). A larger scale reaction yielded 0.329 g (66%). TOF-MS-ASAP$^+$ Calcd. for $[C_{37}H_{21}Cl_6]^+$: 674.9774. Found: *m/z* = 674.9777. Anal. Calcd for $C_{37}H_{21}Cl_6^{\bullet}$: C, 65.52; H, 3.12; Cl, 31.36. Found: C, 65.48; H, 2.93; N, 0.00.

Tris(3,5-dichloro-4'-methyl-[1,1'-biphenyl]-4-yl)methyl radical (4-T_3TTM). αH4-T_3TTM (0.130 g, 0.18 mmol, 1 equiv.), 40% Bu_4NOH (aq) (0.24 mL, 0.36 mmol, 2 equiv.), *p*-chloranil (0.111 g, 0.45 mmol, 2.5 equiv.). The product was collected as red solid (0.104 g, 80%). TOF-MS-ASAP$^+$ Calcd. for $[C_{40}H_{27}Cl_6]^{H+}$: 718.0322. Found: *m/z* = 718.0333. Anal. Calcd for $C_{40}H_{27}Cl_6^{\bullet}$: C, 66.69; H, 3.78; Cl, 29.53. Found: C, 66.01; H, 3.62; N, 0.00.

Tris(3,5-dichloro-3'-methyl-[1,1'-biphenyl]-4-yl)methyl radical (3-T_3TTM). αH3-T_3TTM (0.350 g, 0.49 mmol, 1 equiv.), 40% Bu_4NOH (aq) (0.65 mL, 0.97 mmol, 2 equiv.), *p*-chloranil (0.298 g, 1.21 mmol, 2.5 equiv.). The product was collected as red solid (0.253 g, 72%). TOF-MS-ESI$^+$ Calcd. for

$[C_{40}H_{27}Cl_6]^+$: 717.0244. Found: *m/z* = 717.0210. Anal. Calcd for $C_{40}H_{27}Cl_6^{\bullet}$: C, 66.69; H, 3.78; Cl, 29.53. Found: C, 66.53; H, 3.66; N, 0.00.

Tris(3,5-dichloro-2'-methyl-[1,1'-biphenyl]-4-yl)methyl radical (2-T_3TTM). αH2-T_3TTM (0.150 g, 0.21 mmol, 1 equiv.), 40% Bu_4NOH (aq) (0.28 mL, 0.42 mmol, 2 equiv.), *p*-chloranil (0.128 g, 0.52 mmol, 2.5 equiv.). The product was collected as red solid (0.109 g, 73%). A larger scale reaction yielded 0.196 g (79%). TOF-MS-ESI$^+$ Calcd. for $[C_{40}H_{27}Cl_6]^+$: 717.0244. Found: *m/z* = 717.0210. Anal. Calcd for $C_{40}H_{27}Cl_6^{\bullet}$: C, 66.69; H, 3.78; Cl, 29.53. Found: C, 66.42; H, 3.68; N, 0.00.

Tris(3,5-dichloro-3',5'-dimethyl-[1,1'-biphenyl]-4-yl)methyl radical (3,5-X_3TTM). αH3,5-X_3TTM (0.150 g, 0.20 mmol, 1 equiv.), 40% Bu_4NOH (aq) (0.26 mL, 0.39 mmol, 2 equiv.), *p*-chloranil (0.121 g, 0.49 mmol, 2.5 equiv.). The product was collected as red solid (0.094 g, 63%). TOF-MS-ESI$^+$ Calcd. for $[C_{43}H_{33}Cl_6]^+$: 759.0713. Found: *m/z* = 759.0699. Anal. Calcd for $C_{43}H_{33}Cl_6^{\bullet}$: C, 67.74; H, 4.36; Cl, 27.90. Found: C, 67.24; H, 4.25; N, 0.00.

Tris(3,5-dichloro-2',6'-dimethyl-[1,1'-biphenyl]-4-yl)methyl radical (2,6-X_3TTM). αH2,6-X_3TTM (0.200 g, 0.26 mmol, 1 equiv.), 40% Bu_4NOH (aq) (0.35 mL, 0.52 mmol, 2 equiv.), *p*-chloranil (0.161 g, 0.65 mmol, 2.5 equiv.). The product was collected as red solid (0.138 g, 69%). A larger scale reaction yielded 0.136 g (62%). TOF-MS-ESI$^+$ Calcd. for $[C_{43}H_{33}Cl_6]^+$: 759.0713. Found: *m/z* = 759.0718. Anal. Calcd for $C_{43}H_{33}Cl_6^{\bullet}$: C, 67.74; H, 4.36; Cl, 27.90. Found: C, 68.07; H, 4.49; N, 0.00.

Tris(3,5-dichloro-2',5'-dimethyl-[1,1'-biphenyl]-4-yl)methyl radical (2,5-X_3TTM). αH2,5-X_3TTM (0.250 g, 0.33 mmol, 1 equiv.), 40% Bu_4NOH (aq) (0.44 mL, 0.65 mmol, 2 equiv.), *p*-chloranil (0.201 g, 0.82 mmol, 2.5 equiv.). The product was collected as red solid (0.215 g, 86%). TOF-MS-

ESI$^+$ Calcd. for $[C_{43}H_{33}Cl_6]^+$: 759.0713. Found: *m/z* = 759.0691. Anal. Calcd for $C_{43}H_{33}Cl_6^{\bullet}$: C, 67.74; H, 4.36; Cl, 27.90. Found: C, 66.38; H, 4.15; N, 0.00.

Tris(3,5-dichloro-2',4'-dimethyl-[1,1'-biphenyl]-4-yl)methyl radical (2,4-X$_3$TTM). αH2,4-X$_3$TTM (0.150 g, 0.20 mmol, 1 equiv.), 40% Bu_4NOH (aq) (0.26 mL, 0.39 mmol, 2 equiv.), *p*-chloranil (0.121 g, 0.49 mmol, 2.5 equiv.). The product was collected as red solid (0.135 g, 90%). TOF-MS-ESI$^+$ Calcd. for $[C_{43}H_{33}Cl_6]^+$: 759.0713. Found: *m/z* = 759.0694. Anal. Calcd for $C_{43}H_{33}Cl_6^{\bullet}$: C, 67.74; H, 4.36; Cl, 27.90. Found: C, 67.52; H, 4.27; N, 0.00.

Tris(3,5-dichloro-2',4',6'-trimethyl-[1,1'-biphenyl]-4-yl)methyl radical (M$_3$TTM). αHM$_3$TTM (0.110 g, 0.14 mmol, 1 equiv.), 40% Bu_4NOH (aq) (0.18 mL, 0.27 mmol, 2 equiv.), *p*-chloranil (0.084 g, 0.34 mmol, 2.5 equiv.). The product was collected as red solid (0.094 g, 86%). A larger scale reaction yielded 0.470 g (78%). TOF-MS-ASAP$^+$ Calcd. for $[C_{46}H_{39}Cl_6]^+$: 801.1183. Found: *m/z* = 801.1182. Anal. Calcd for $C_{46}H_{39}Cl_6^{\bullet}$: C, 68.68; H, 4.89; Cl, 26.44. Found: C, 68.00; H, 4.72; N, 0.00.

Tris(3,5-dichloro-2',6'-diisopropyl-[1,1'-biphenyl]-4-yl)methyl radical (2,6-ipP$_3$TTM). αH2,6-ipP$_3$TTM (0.040 g, 0.04 mmol, 1 equiv.), 40% Bu_4NOH (aq) (0.06 mL, 0.09 mmol, 2 equiv.), *p*-chloranil (0.026 g, 0.11 mmol, 2.5 equiv.). 1:1 (v/v) THF/DMSO solvent ratio was used. The product was collected as red solid (0.034 g, 85%). TOF-MS-ESI$^+$ Calcd. for $[C_{55}H_{57}Cl_6]^+$: 927.2591. Found: *m/z* = 927.2599. Anal. Calcd for $C_{55}H_{57}Cl_6^{\bullet}$: C, 70.97; H, 6.17; Cl, 22.85. Found: C, 71.17; H, 6.37; N, 0.00.

S3. Optical Spectroscopy

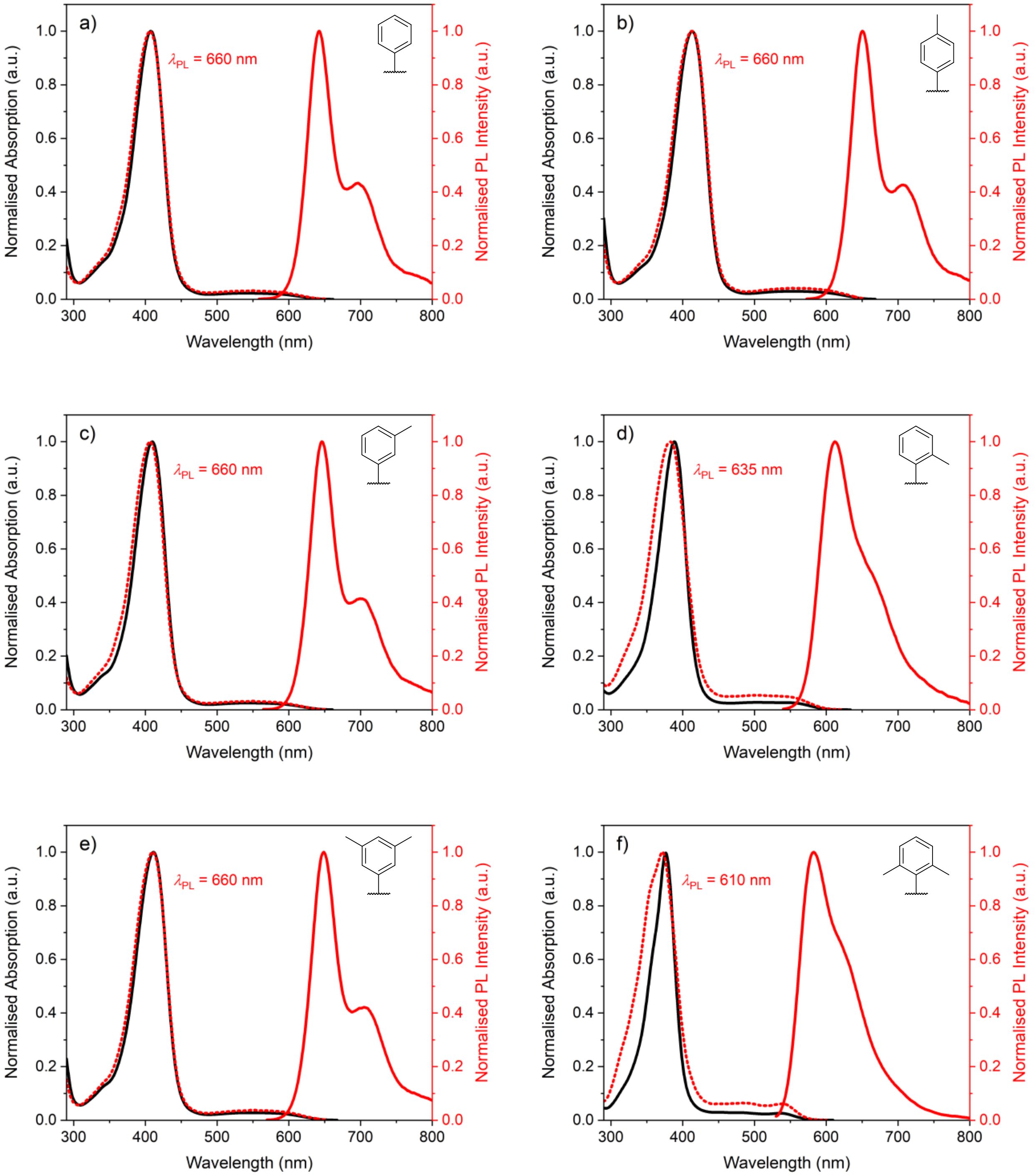

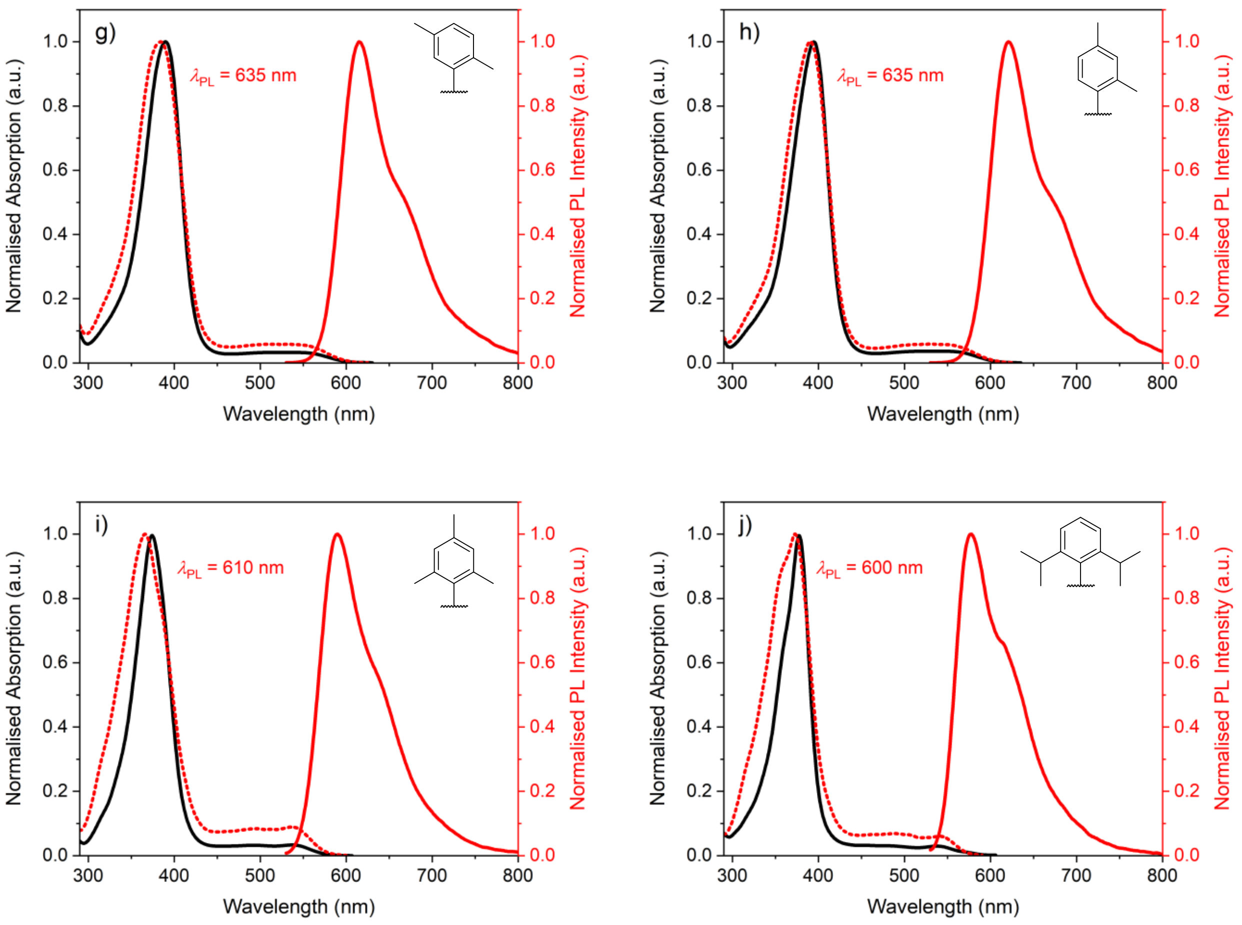

Figure S3. Optical absorption (black, solid lines) and photoluminescence spectra following 520 nm excitation (red, solid lines) for a) P_3TTM, b) 4-T_3TTM, c) 3-T_3TTM, d) 2-T_3TTM, e) 3,5-X_3TTM, f) 2,6-X_3TTM, g) 2,5-X_3TTM, h) 2,4-X_3TTM, i) M_3TTM and j) 2,6-ipP_3TTM in 0.1 mM toluene solution. Excitation spectra (red, dotted lines) for the corresponding radicals in dilute (1 µM) toluene solution with the measured photoluminescence wavelengths shown for each excitation spectrum. The respective aryl groups are shown in the insets.

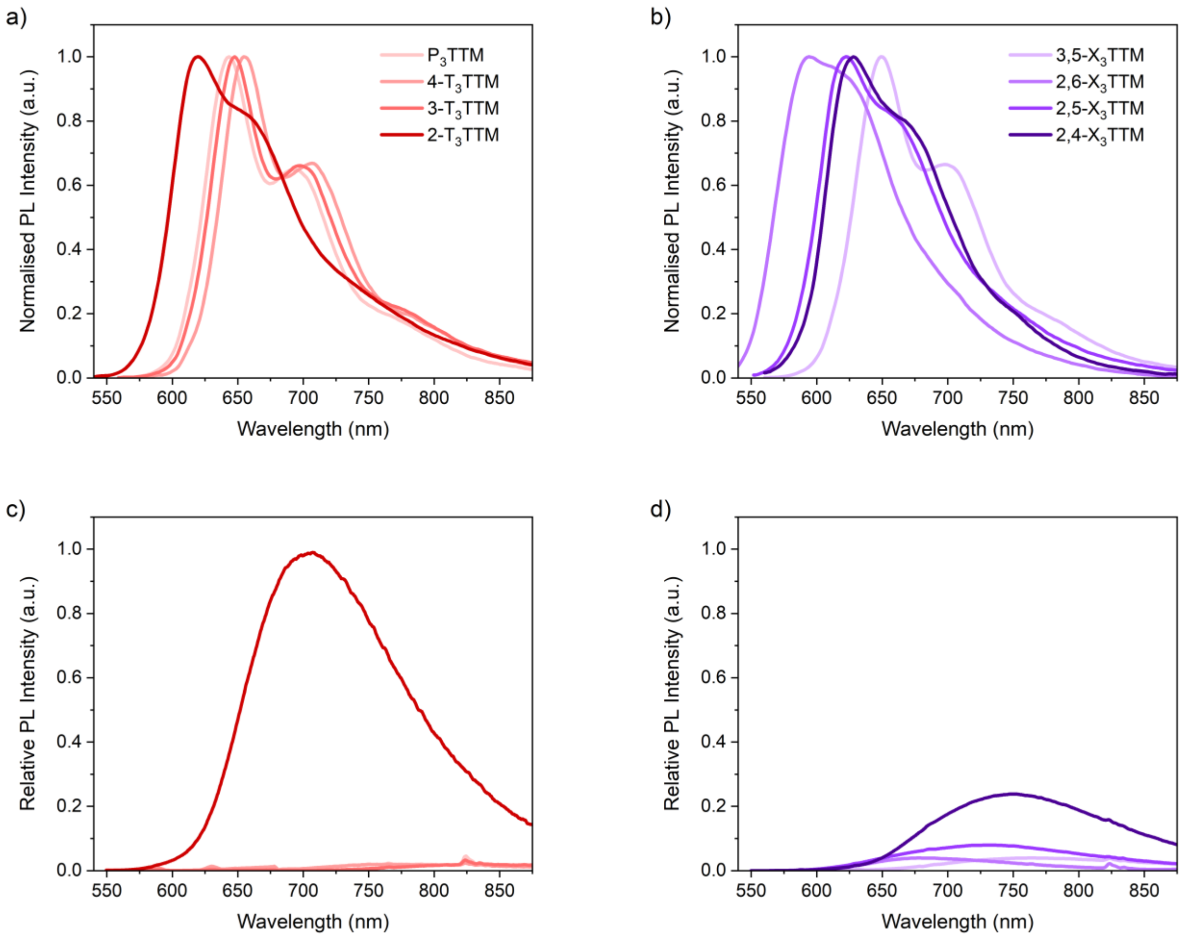

Figure S4. Steady-state photoluminescence of a) phenyl and tolyl and b) xylyl substituted radicals in 3 wt% drop-cast films in inert PMMA host following 405 nm excitation. Photoluminescence relative to the PLQE of c) phenyl and tolyl and d) xylyl substituted radicals in the neat crystalline solid state following 405 nm excitation.

Table S1. Summary of photophysical parameters of radicals in the crystalline solid state

Radical	λ_{PL}, crystal (nm)	Φ_{PL}, crystal (%)
P_3TTM	750–815	<1
4-T_3TTM	750–815	<1
3-T_3TTM	810–855	<1
2-T_3TTM	706	25
3,5-X_3TTM	765	<1
2,6-X_3TTM	683	<1
2,5-X_3TTM	732	2
2,4-X_3TTM	751	6
M_3TTM	646	5
2,6-ipP_3TTM	651	8

S4. DFT Calculations

Table S2. Frontier molecular orbital energy levels of trityl radicals (UB3LYP)

Radical	HOMOα	HOMOβ	SOMOα	SOMOβ	LUMOα	LUMOβ
P_3TTM	–6.41	–6.40	–5.20	–3.09	–1.53	–1.47
4-T_3TTM	–6.22	–6.21	–5.08	–3.00	–1.42	–1.37
3-T_3TTM	–6.33	–6.32	–5.14	–3.04	–1.45	–1.40
2-T_3TTM	–6.47	–6.46	–5.25	–3.07	–1.34	–1.27
3,5-X_3TTM	–6.25	–6.23	–5.08	–2.98	–1.39	–1.34
2,6-X_3TTM	–6.64	–6.64	–5.36	–3.08	–1.11	–1.00
2,5-X_3TTM	–6.34	–6.33	–5.19	–3.01	–1.28	–1.22
2,4-X_3TTM	–6.28	–6.27	–5.15	–2.99	–1.26	–1.20
M_3TTM	–6.46	–6.45	–5.29	–3.01	–1.05	–0.94
2,6-ipP_3TTM	–6.60	–6.59	–5.36	–3.09	–1.13	–1.02

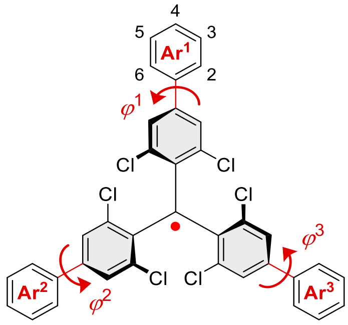

Table S3. Dihedrals and lengths of phenyl-phenyl bonds (red) of the corresponding aryl groups of trityl radicals in their optimized D_0 (UB3LYP) and D_1 geometries (UPBE0)

Radical	**φ^1 D_0 (°)**	**φ^1 D_1 (°)**	φ^2 D_0 (°)	φ^2 D_1 (°)	φ^3 D_0 (°)	φ^3 D_1 (°)	**Ar^1 D_0 (Å)**	**Ar^1 D_1 (Å)**	Ar^2 D_0 (Å)	Ar^2 D_1 (Å)	Ar^3 D_0 (Å)	Ar^3 D_1 (Å)
P_3TTM	**33.9**	**10.4**	33.6	32.4	33.9	32.4	**1.482**	**1.440**	1.481	1.475	1.482	1.475
4-T_3TTM	**32.9**	**8.6**	32.6	31.3	32.8	31.3	**1.481**	**1.437**	1.480	1.474	1.481	1.474
3-T_3TTM	**33.9**	**9.1**	33.9	32.4	34.0	32.4	**1.482**	**1.438**	1.482	1.475	1.482	1.475
2-T_3TTM	**53.5**	**37.7**	54.4	51.5	53.5	51.8	**1.485**	**1.456**	1.486	1.480	1.486	1.480
3,5-X_3TTM	**34.0**	**8.7**	33.7	32.6	34.0	32.6	**1.482**	**1.436**	1.481	1.475	1.482	1.475
2,6-X_3TTM	**84.3**	**60.7**	84.3	89.0	84.5	89.3	**1.492**	**1.475**	1.492	1.487	1.492	1.487
2,5-X_3TTM	**53.7**	**36.5**	52.8	51.0	53.8	51.1	**1.486**	**1.454**	1.485	1.480	1.486	1.480
2,4-X_3TTM	**52.9**	**35.6**	52.3	51.2	52.9	50.1	**1.485**	**1.452**	1.484	1.479	1.485	1.479
M_3TTM	**83.5**	**55.6**	83.2	88.9	82.9	88.9	**1.491**	**1.469**	1.491	1.486	1.491	1.486
2,6-ipP_3TTM	**88.4**	**65.8**	86.4	83.3	86.6	83.3	**1.493**	**1.477**	1.493	1.488	1.493	1.488

Table S4. Excited state analysis of trityl radicals (UPBE0 and UM06-2X)

Radical	Method	D_1 [a]	E (eV)[b]	f [c]	D index (Å)[d]	S_r index (a.u.)[e]	t index (Å)[f]	Orbital config.
P_3TTM	PBE0	vert.	2.46	0.0169	0.589	0.864	–2.519	Hβ–Lβ: 61.8%<br>Hα–Lα: 17.6%
		adia.	2.20	0.0181	0.734	0.868	–2.442	Hβ–Lβ: 64.9%<br>Hα–Lα: 20.1%
	M062X	vert.	2.82	0.0213	0.530	0.842	–2.255	Hβ–Lβ: 60.4%<br>Hα–Lα: 16.4%
		adia.	2.40	0.0400	0.636	0.849	–2.193	Hβ–Lβ: 69.9%<br>Hα–Lα: 16.0%
4-T_3TTM	PBE0	vert.	2.43	0.0258	0.752	0.851	–2.506	Hβ–Lβ: 61.9%<br>Hα–Lα: 16.4%
		adia.	2.17	0.0234	0.863	0.864	–2.477	Hβ–Lβ: 63.8%<br>Hα–Lα: 20.3%
	M062X	vert.	2.79	0.0296	0.622	0.832	–2.293	Hβ–Lβ: 58.0%<br>Hα–Lα: 15.5%
		adia.	2.37	0.0497	0.719	0.849	–1.825	Hβ–Lβ: 68.0%<br>Hα–Lα: 16.1%
3-T_3TTM	PBE0	vert.	2.46	0.0200	0.638	0.858	–2.136	Hβ–Lβ: 59.1%<br>Hα–Lα: 15.2%
		adia.	2.18	0.0186	0.776	0.869	–2.173	Hβ–Lβ: 63.2%<br>Hα–Lα: 20.7%
	M062X	vert.	2.82	0.0296	0.598	0.837	–1.892	Hβ–Lβ: 56.1%<br>Hα–Lα: 15.0%
		adia.	2.39	0.0426	0.658	0.850	–1.724	Hβ–Lβ: 68.8%<br>Hα–Lα: 16.1%
2-T_3TTM	PBE0	vert.	2.57	0.0251	0.713	0.830	–1.824	Hβ–Lβ: 62.1%<br>Hα–L+1α: 8.6%
		adia.	2.28	0.0385	1.039	0.796	–1.405	Hβ–Lβ: 76.7%<br>Hα–Lα: 11.3%
	M062X	vert.	2.92	0.0269	0.635	0.813	–1.671	Hβ–Lβ: 55.6%<br>H–1β–Lβ: 14.4%
		adia.	2.47	0.0508	0.733	0.819	–1.466	Hβ–Lβ: 72.9%<br>Hα–Lα: 12.0%
3,5-X_3TTM	PBE0	vert.	2.45	0.0227	0.718	0.853	–2.472	Hβ–Lβ: 61.6%<br>Hα–Lα: 16.4%
		adia.	2.17	0.0195	0.802	0.872	–2.516	Hβ–Lβ: 62.0%<br>Hα–Lα: 21.1%
	M062X	vert.	2.81	0.0261	0.595	0.834	–2.246	Hβ–Lβ: 58.3%<br>Hα–Lα: 15.6%
		adia.	2.38	0.0441	0.665	0.854	–2.255	Hβ–Lβ: 67.9%<br>Hα–Lα: 16.6%
2,6-X_3TTM	PBE0	vert.	2.69	0.0123	0.422	0.822	–1.662	H–3β–Lβ: 34.8%<br>H–9β–Lβ: 17.5%
		adia.	2.41	0.0296	1.031	0.744	–1.249	Hβ–Lβ: 80.4%<br>Hα–Lα: 7.3%

	M062X	vert.	3.02	0.0173	0.481	0.804	–1.566	H–3β–Lβ: 30.0%<br>H–9β–Lβ: 22.2%
		adia.	2.56	0.0422	0.742	0.781	–1.474	Hβ–Lβ: 76.7%<br>Hα–Lα: 8.9%
2,5-X_3TTM	PBE0	vert.	2.56	0.0284	0.774	0.823	–2.064	Hβ–Lβ: 51.8%<br>H–4β–Lβ: 10.5%
		adia.	2.26	0.0412	1.139	0.801	–1.783	Hβ–Lβ: 73.7%<br>Hα–Lα: 11.8%
	M062X	vert.	2.91	0.0290	0.695	0.811	–1.828	Hβ–Lβ: 44.0%<br>H–1β–Lβ: 13.7%
		adia.	2.45	0.0544	0.755	0.819	–1.486	Hβ–Lβ: 70.0%<br>Hα–Lα: 11.9%
2,4-X_3TTM	PBE0	vert.	2.54	0.0334	0.868	0.814	–1.511	Hβ–Lβ: 58.2%<br>H–1β–Lβ: 11.6%
		adia.	2.25	0.0467	1.235	0.798	–1.437	Hβ–Lβ: 74.5%<br>Hα–Lα: 11.7%
	M062X	vert.	2.90	0.0314	0.666	0.811	–1.622	Hβ–Lβ: 45.9%<br>H–1β–Lβ: 23.3%
		adia.	2.44	0.0610	0.849	0.814	–1.413	Hβ–Lβ: 70.7%<br>Hα–Lα: 11.6%
M_3TTM	PBE0	vert.	2.69	0.0141	0.447	0.822	–1.779	H–6β–Lβ: 46.5%<br>H–1β–Lβ: 16.0%
		adia.	2.38	0.0393	1.281	0.749	–1.240	Hβ–Lβ: 73.8%<br>Hα–Lα: 7.8%
	M062X	vert.	3.20	0.0189	0.469	0.803	–1.613	H–6β–Lβ: 45.9%<br>H–1β–Lβ: 16.6%
		adia.	2.54	0.0506	0.840	0.781	–1.503	Hβ–Lβ: 69.0%<br>Hα–Lα: 9.1%
2,6-ipP_3TTM	PBE0	vert.	2.67	0.0144	0.462	0.814	–1.585	H–3β–Lβ: 34.4%<br>H–7β–Lβ: 16.0%
		adia.	2.38	0.0310	1.088	0.736	–1.163	Hβ–Lβ: 80.2%<br>Hα–Lα: 6.8%
	M062X	vert.	2.99	0.0197	0.502	0.795	–1.475	H–3β–Lβ: 34.5%<br>H–7β–Lβ: 17.7%
		adia.	2.51	0.0416	0.712	0.786	–1.258	Hβ–Lβ: 74.9%<br>Hα–Lα: 9.2%

[a] Vertical and adiabatic excited state based on optimized D_0 and D_1 geometry, respectively. [b] Excited state energy. [c] Oscillator strength. [d] Distance between centroid of hole and electron. [e] Overlap integral of hole and electron distribution. [f] Separation of hole and electron in direction of charge transfer (larger negative value implies that hole and electron are less separated).

Table S5. Excited state analysis of structures from X-ray crystallography (UPBE0)

Radical	Mol. index [a]	E (eV)[b]	*f* [c]	*D* index (Å)[d]	S_r index (a.u.)[e]	*t* index (Å)[f]	Orbital config.
P_3TTM	1	2.49	0.0150	0.441	0.867	–1.841	Hβ–Lβ: 51.0%<br>Hα–Lα: 18.2%
	2	2.61	0.0082	0.452	0.886	–1.973	Hβ–Lβ: 52.5%<br>Hα–Lα: 21.3%
	3	2.53	0.0244	0.463	0.858	–1.326	Hβ–Lβ: 62.0%<br>Hα–L+1α: 14.2%
	4	2.51	0.0175	0.618	0.865	–2.500	Hβ–Lβ: 52.5%<br>Hα–Lα: 11.9%
4-T_3TTM	1	2.57	0.0145	0.644	0.855	–1.391	H–1β–Lβ: 62.0%<br>Hα–Lα: 17.8%
	2	2.41	0.0292	0.590	0.849	–2.426	Hβ–Lβ: 58.4%<br>Hα–L+1α: 10.5%
	3	2.52	0.0154	0.774	0.851	–2.408	H–1β–Lβ: 52.5%<br>Hα–Lα: 14.4%
3-T_3TTM	1	2.52	0.0231	0.261	0.865	–2.003	H–1β–Lβ: 44.5%<br>Hα–L+1α: 16.7%
	2	2.52	0.0243	0.472	0.863	–1.398	Hβ–Lβ: 59.2%<br>Hα–L+1α: 18.0%
2-T_3TTM	1	2.67/<br>2.68 [g]	0.0143/<br>0.0136 [g]	0.976/<br>0.815 [g]	0.814/<br>0.836 [g]	–1.565/<br>–1.436 [g]	Hβ–Lβ: 57.0/56.7% [g]<br>Hα–Lα: 12.2/12.1% [g]
3,5-X_3TTM	1	2.43	0.0378	0.301	0.841	–2.839	Hβ–Lβ: 67.6%<br>Hα–L+1α: 14.9%
2,6-X_3TTM	1	2.76	0.0128	0.079	0.832	–2.106	H–4β–Lβ: 47.1%<br>H–9β–Lβ: 25.4%
2,4-X_3TTM	1	2.67	0.0259	0.714	0.792	–1.710	Hβ–Lβ: 56.7%<br>H–9β–Lβ: 11.1%
	2	2.60	0.0283	0.774	0.825	–1.901	Hβ–Lβ: 41.0%<br>H–1β–Lβ: 25.9%
M_3TTM	1	2.73	0.0158	0.499	0.804	–1.123	H–7β–Lβ: 32.4%<br>H–1β–Lβ: 29.3%
2,6-ipP_3TTM	1	2.72	0.0156	0.794	0.795	–1.474	H–1β–Lβ: 35.8%<br>H–3β–Lβ: 28.0%

[a] Molecular structure from X-ray crystallography (Table S8). [b] Vertical excited state energy based on X-ray crystallographic geometry (see Section S6). [c] Oscillator strength. [d] Distance between centroid of hole and electron. [e] Overlap integral of hole and electron distribution. [f] Separation of hole and electron in direction of charge transfer (larger negative value implies that hole and electron are less separated). [g] The molecule shows 2-fold disorder of one 2-methylphenyl ring (see Section S6), both components are calculated.

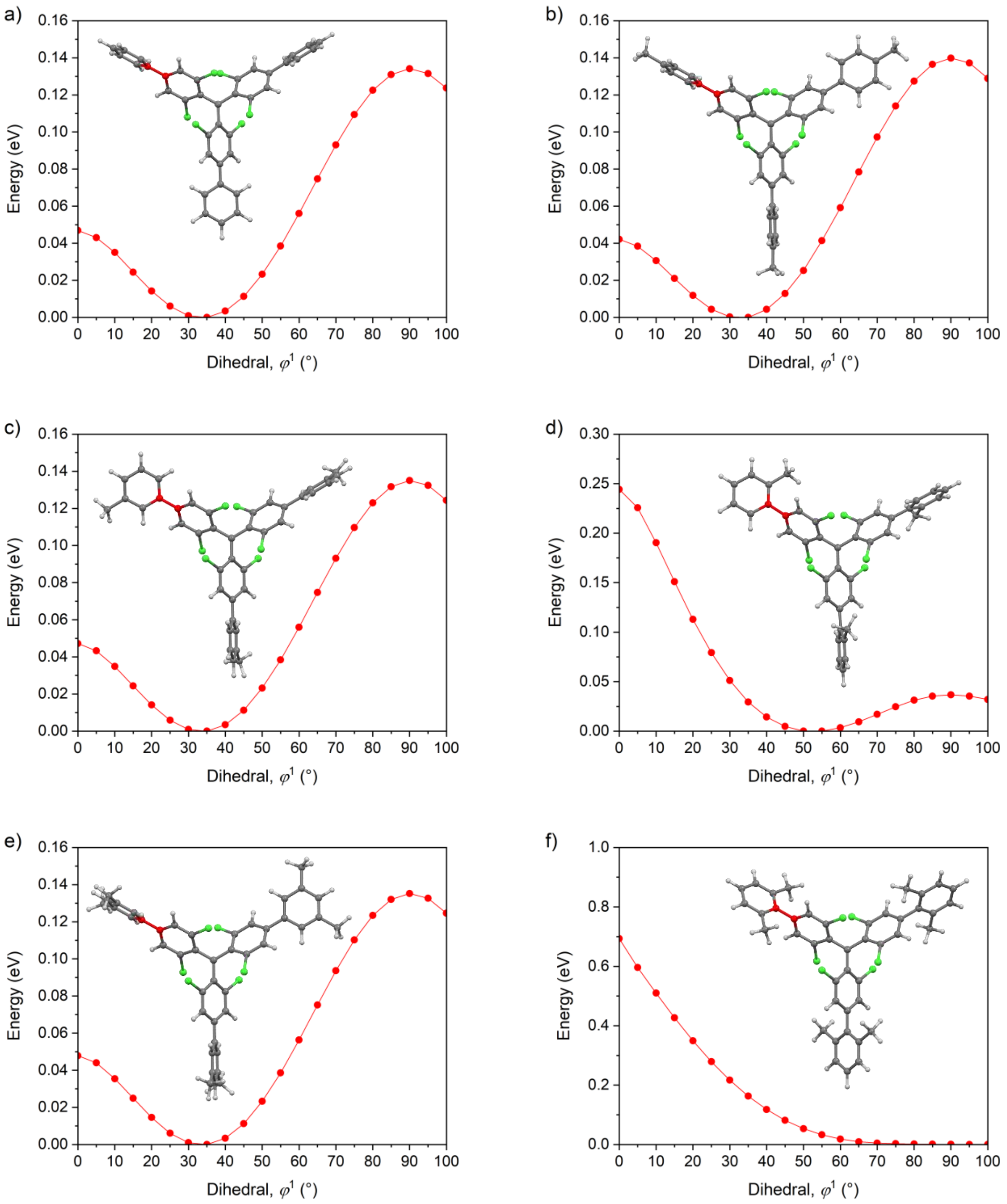
a)
b)
c)
d)
e)
f)
Energy (eV)
Dihedral, φ1 (°)

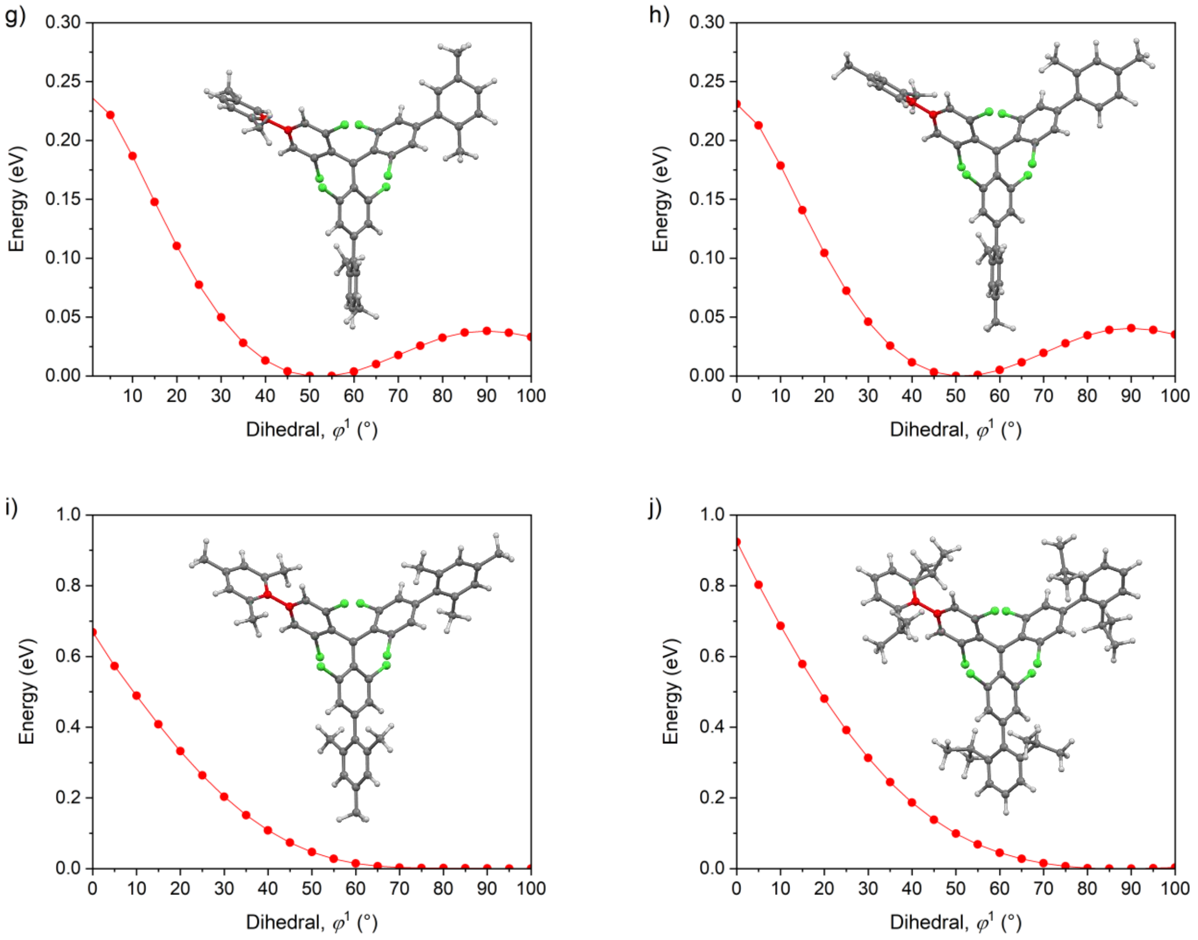

Figure S5. Potential energy as a function of dihedral for aryl group Ar^1 in a) P$_3$TTM, b) 4-T$_3$TTM, c) 3-T$_3$TTM, d) 2-T$_3$TTM, e) 3,5-X$_3$TTM, f) 2,6-X$_3$TTM, g) 2,5-X$_3$TTM, h) 2,4-X$_3$TTM, i) M$_3$TTM and j) 2,6-ipP$_3$TTM. The corresponding phenyl-phenyl bond is coloured red in the optimized ground state geometries in insets.

Electronic structure of optimized ground state geometries

P_3TTM

	α	β
LUMO	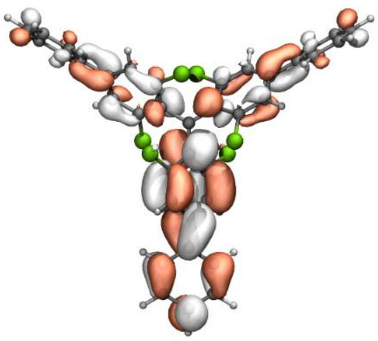<br>174a = –1.53 eV Occ.: 0	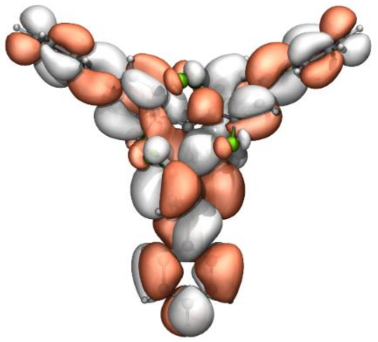<br>174b = –1.47 eV Occ.: 0
SOMO	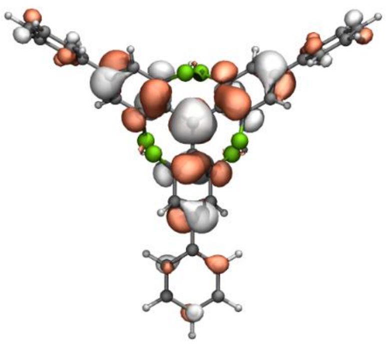<br>173a = –5.20 eV Occ.: 1	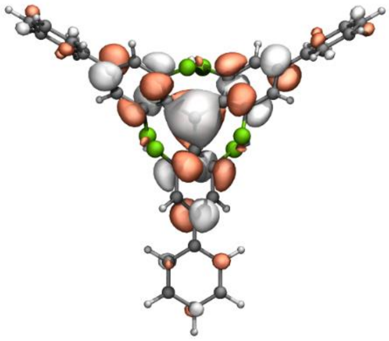<br>173b = –3.09 eV Occ.: 0
HOMO	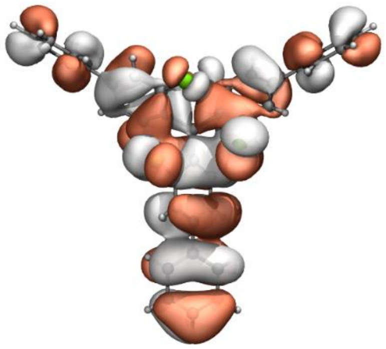<br>172a = –6.41 eV Occ.: 1	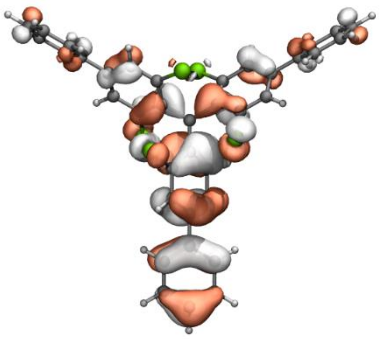<br>172b = –6.40 eV Occ.: 1
HOMO–1	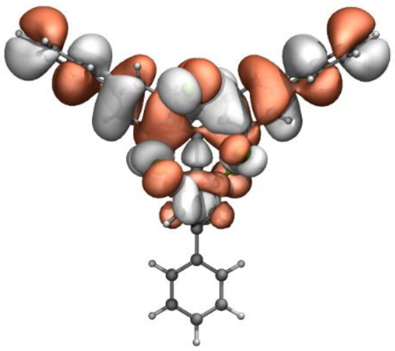<br>171a = –6.42 eV Occ.: 1	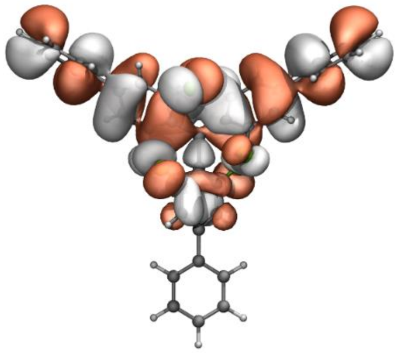<br>171b = –6.41 eV Occ.: 1

4-T_3TTM

	α	β
LUMO	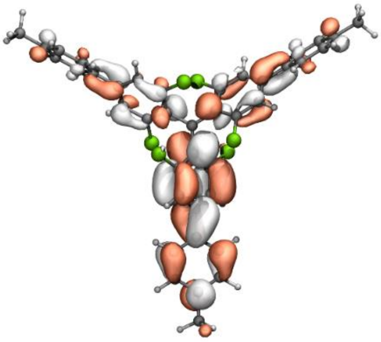 186a = –1.42 eV Occ.: 0	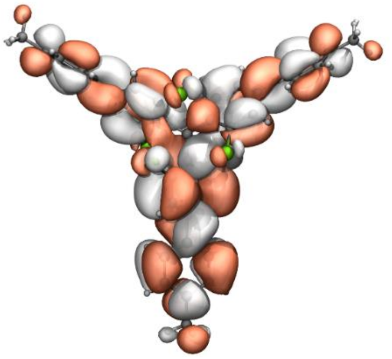 186b = –1.37 eV Occ.: 0
SOMO	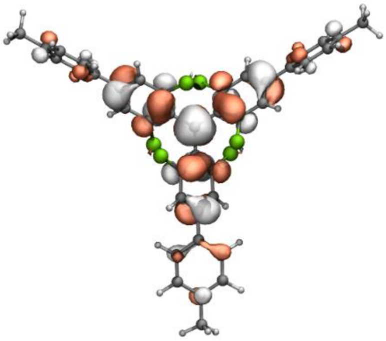 185a = –5.08 eV Occ.: 1	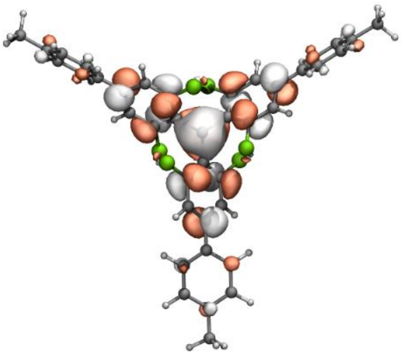 185b = –3.00 eV Occ.: 0
HOMO	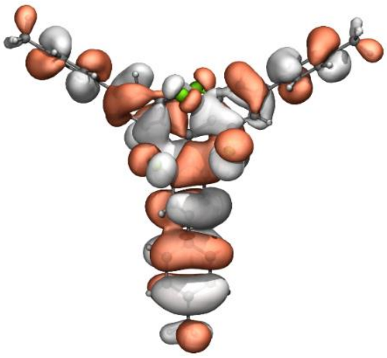 184a = –6.22 eV Occ.: 1	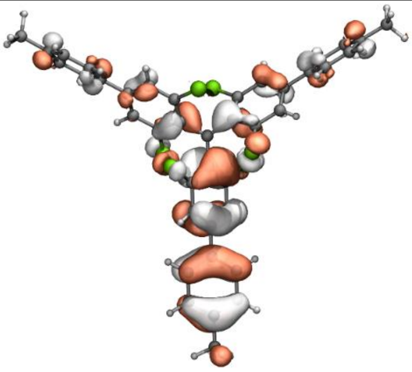 184b = –6.21 eV Occ.: 1
HOMO–1	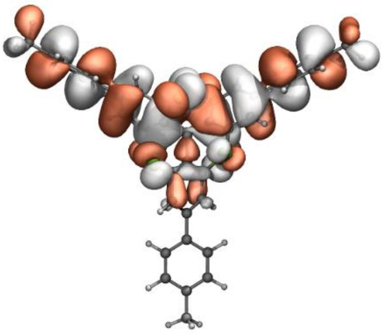 183a = –6.23 eV Occ.: 1	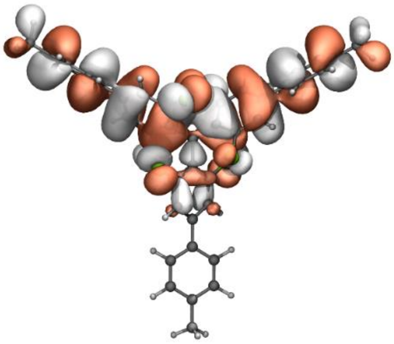 183b = –6.22 eV Occ.: 1

3-T_3TTM

	α	β
LUMO	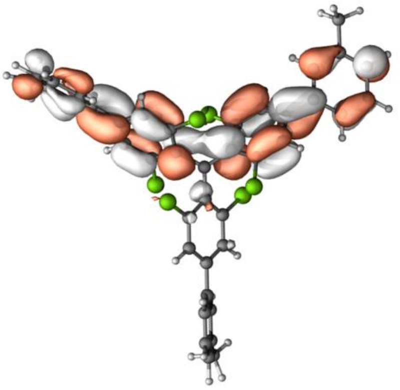 186a = –1.45 eV Occ.: 0	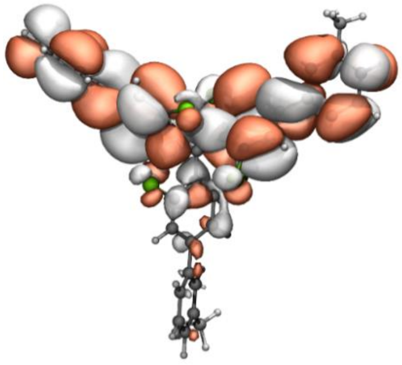 186b = –1.40 eV Occ.: 0
SOMO	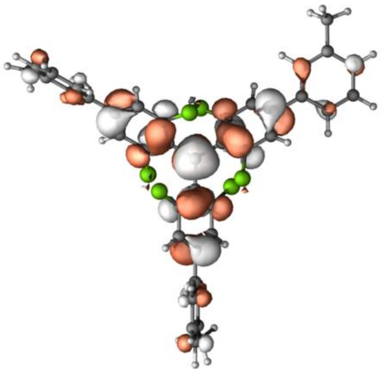 185a = –5.14 eV Occ.: 1	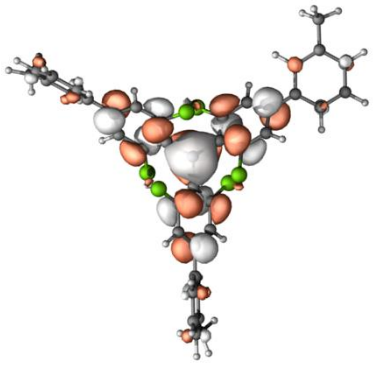 185b = –3.04 eV Occ.: 0
HOMO	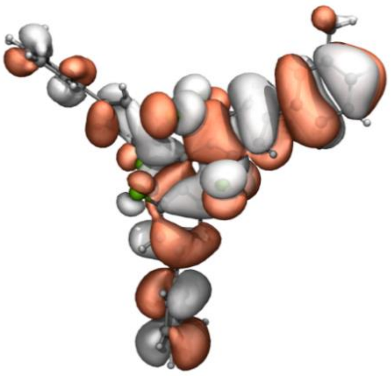 184a = –6.33 eV Occ.: 1	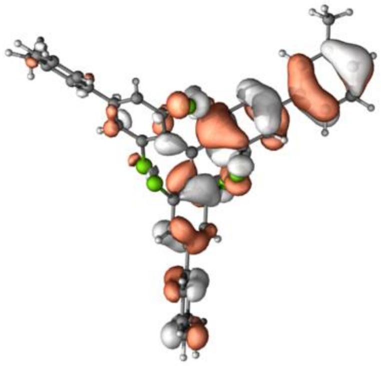 184b = –6.32 eV Occ.: 1
HOMO–1	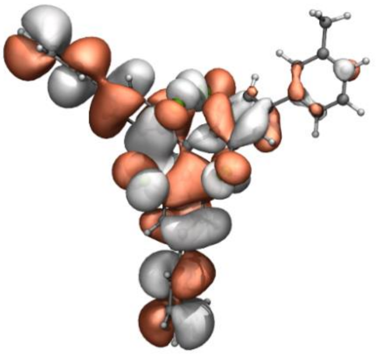 183a = –6.34 eV Occ.: 1	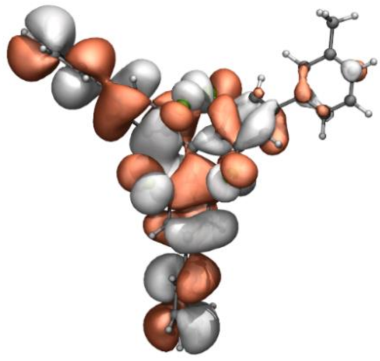 183b = –6.32 eV Occ.: 1

2-T$_3$TTM

	α	β
LUMO+1	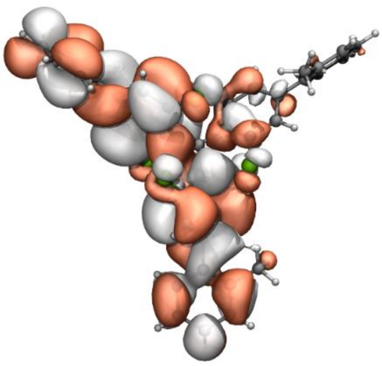 187a = –1.31 eV Occ.: 0	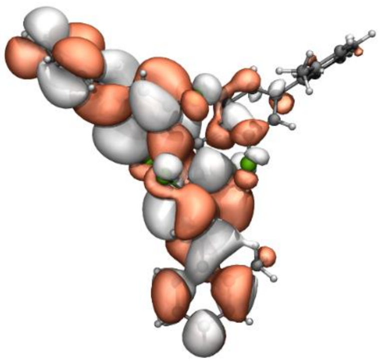 187b = –1.25 eV Occ.: 0
LUMO	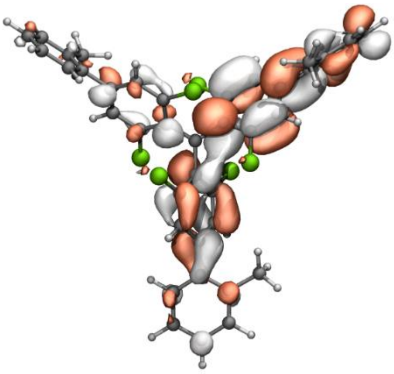 186a = –1.34 eV Occ.: 0	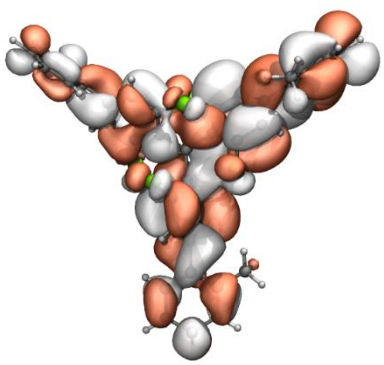 186b = –1.27 eV Occ.: 0
SOMO	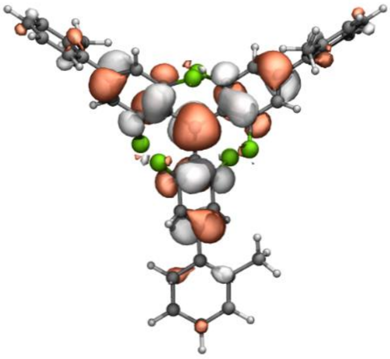 185a = –5.25 eV Occ.: 1	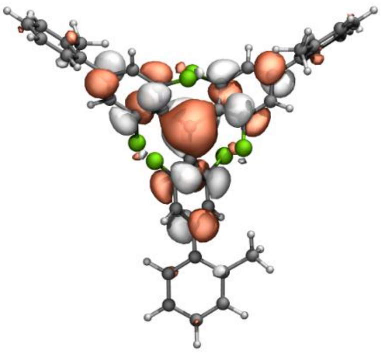 185b = –3.07 eV Occ.: 0
HOMO	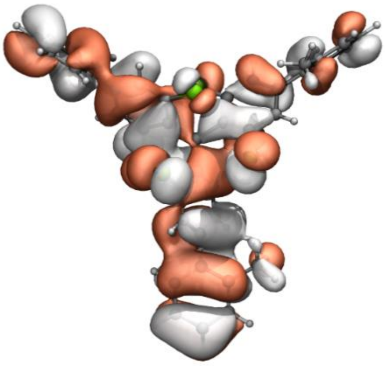 184a = –6.47 eV Occ.: 1	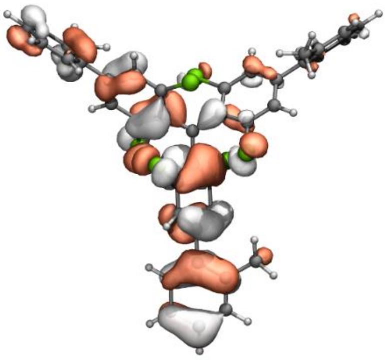 184b = –6.46 eV Occ.: 1

HOMO–1	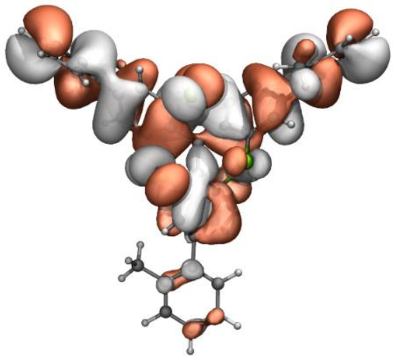 183a = –6.50 eV Occ.: 1	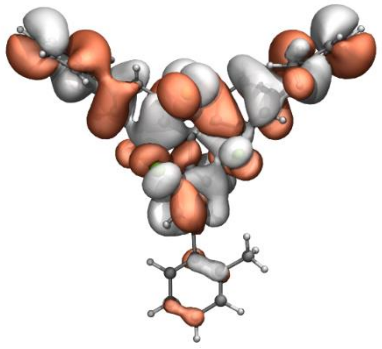 183b = –6.49 eV Occ.: 1

3,5-X_3TTM

	α	β
LUMO	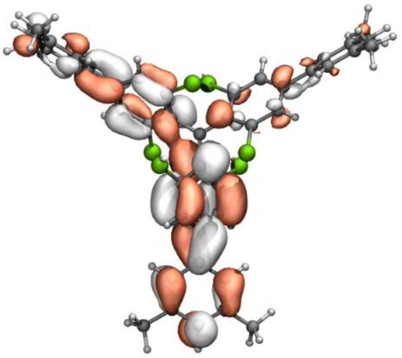 186a = –1.39 eV Occ.: 0	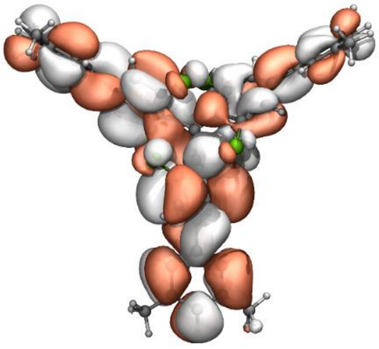 186b = –1.34 eV Occ.: 0
SOMO	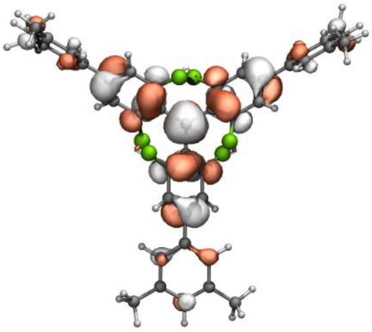 185a = –5.08 eV Occ.: 1	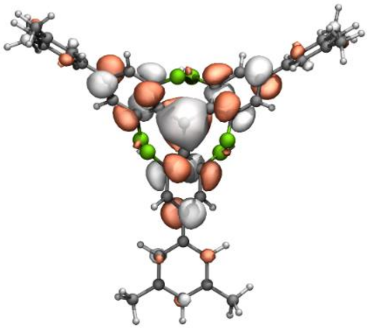 185b = –2.98 eV Occ.: 0
HOMO	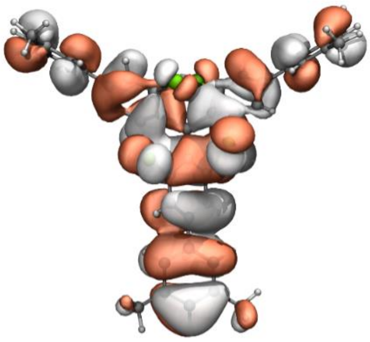 184a = –6.25 eV Occ.: 1	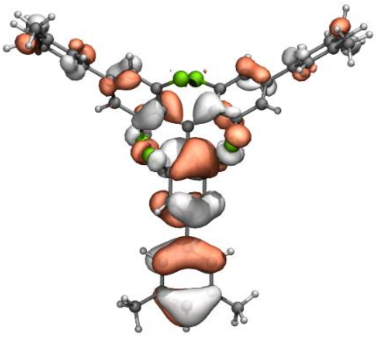 184b = –6.23 eV Occ.: 1

HOMO–1	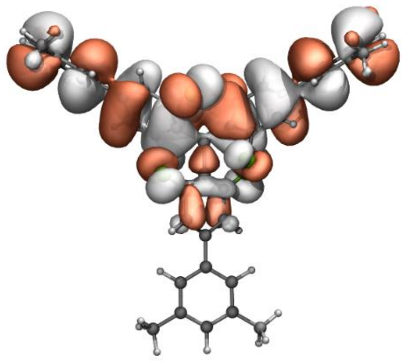 183a = –6.26 eV Occ.: 1	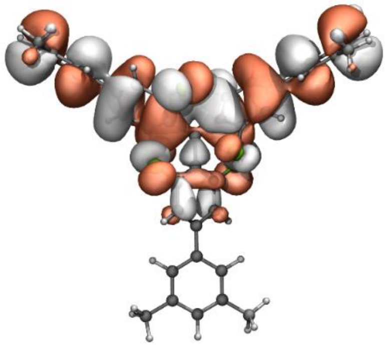 183b = –6.24 eV Occ.: 1

2,6-X_3TTM

	α	β
LUMO	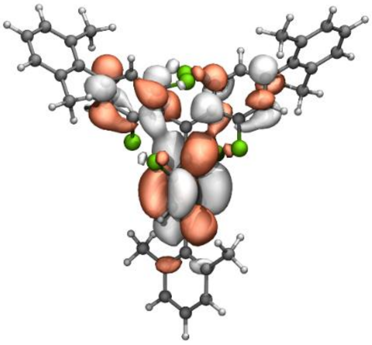 198a = –1.11 eV Occ.: 0	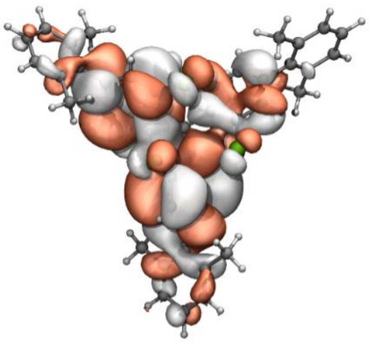 198b = –1.00 eV Occ.: 0
SOMO	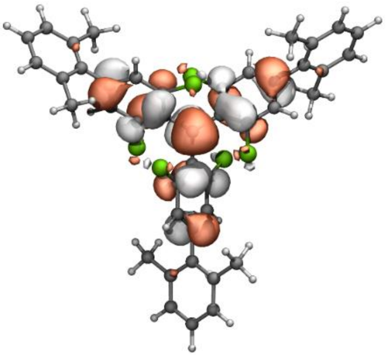 197a = –5.36 eV Occ.: 1	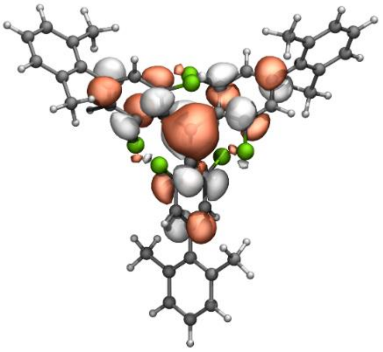 197b = –3.08 eV Occ.: 0
HOMO	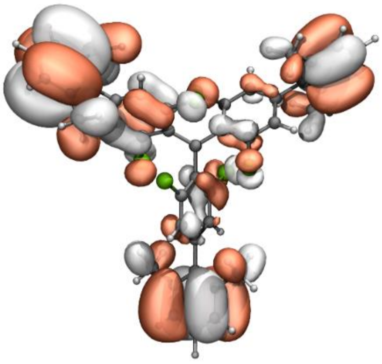 196a = –6.64 eV Occ.: 1	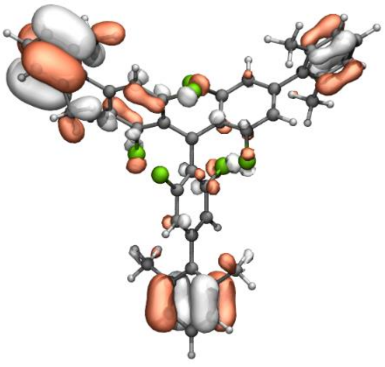 196b = –6.64 eV Occ.: 1

HOMO–1	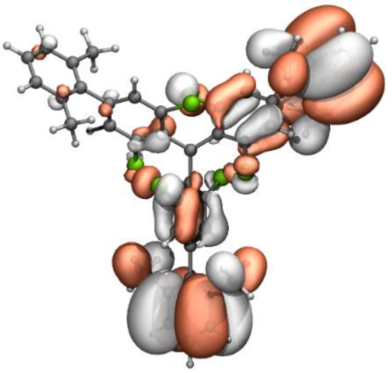<br>195a = –6.64 eV Occ.: 1	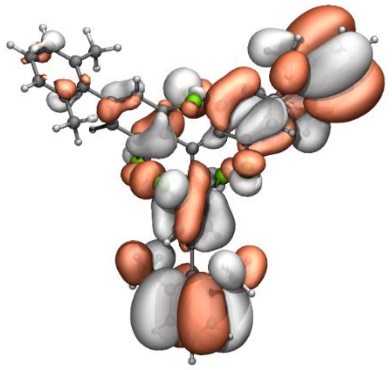<br>195b = –6.64 eV Occ.: 1
HOMO–2	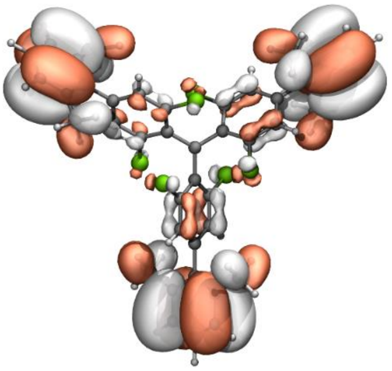<br>194a = –6.65 eV Occ.: 1	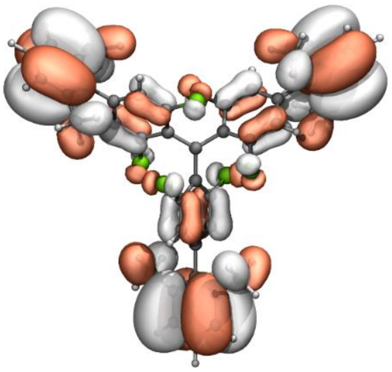<br>194b = –6.64 eV Occ.: 1
HOMO–3	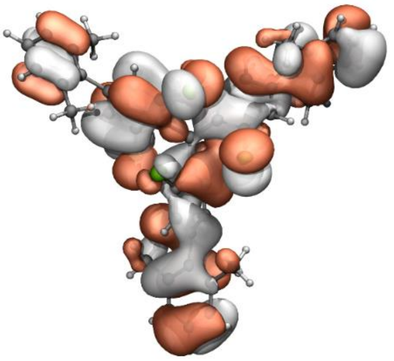<br>193a = –6.71 eV Occ.: 1	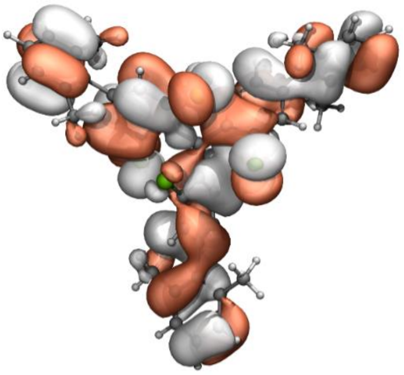<br>193b = –6.69 eV Occ.: 1
HOMO–4	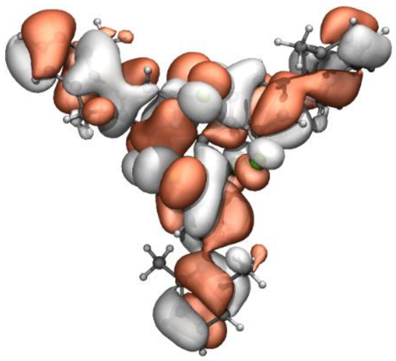<br>192a = –6.71 eV Occ.: 1	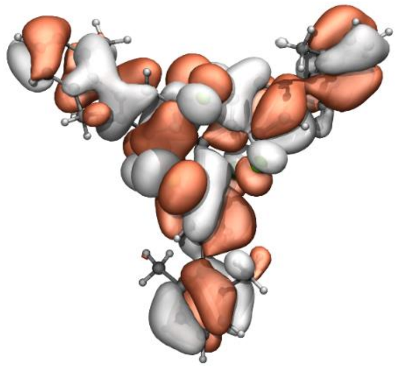<br>192b = –6.69 eV Occ.: 1

HOMO–5	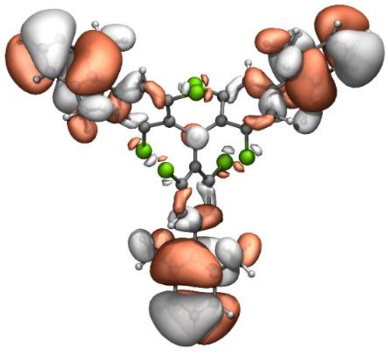 191a = –6.76 eV Occ.: 1	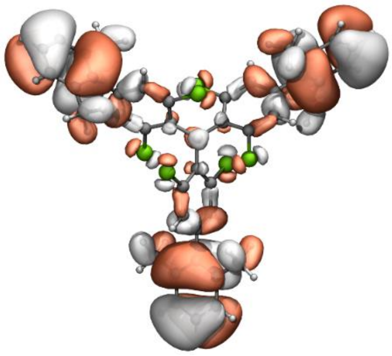 191b = –6.75 eV Occ.: 1
HOMO–6	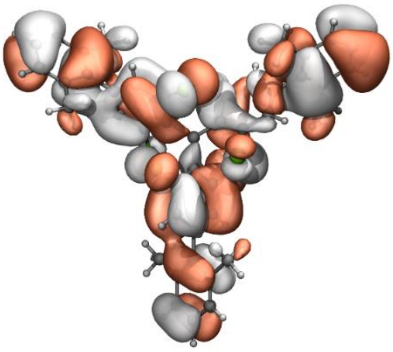 190a = –6.77 eV Occ.: 1	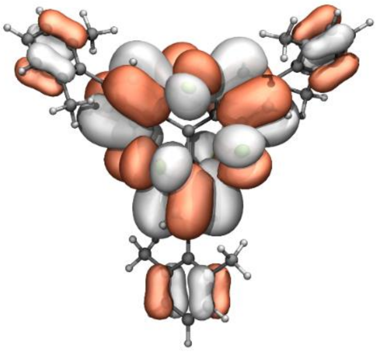 190b = –6.76 eV Occ.: 1
HOMO–7	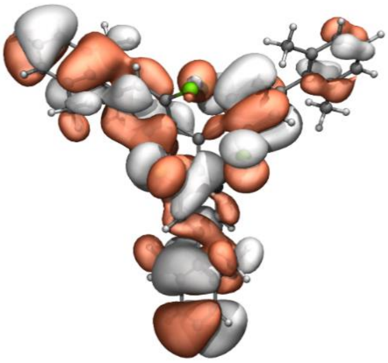 189a = –6.77 eV Occ.: 1	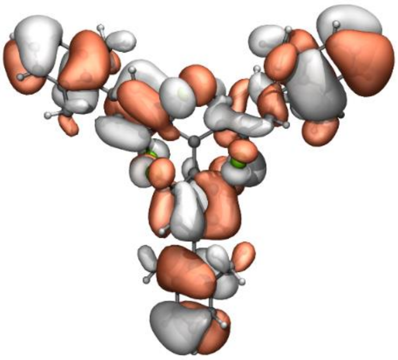 189b = –6.76 eV Occ.: 1
HOMO–8	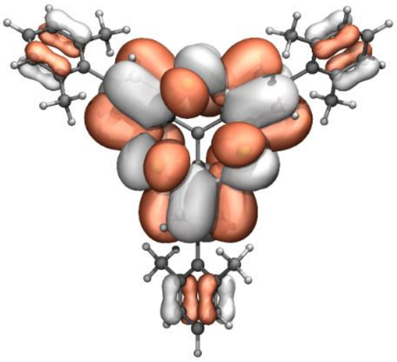 188a = –6.79 eV Occ.: 1	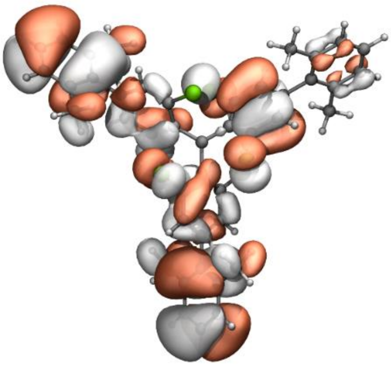 188b = –6.76 eV Occ.: 1

HOMO–9	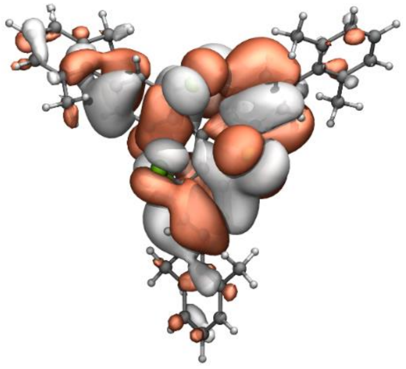 187a = –6.92 eV Occ.: 1	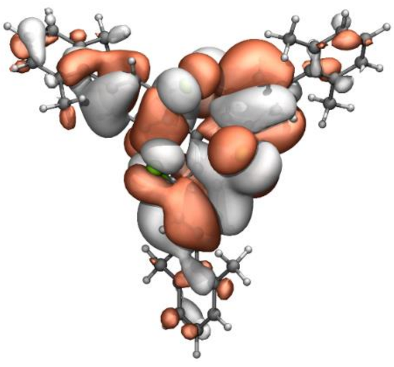 187b = –6.92 eV Occ.: 1

2,5-X_3TTM

	α	β
LUMO+1	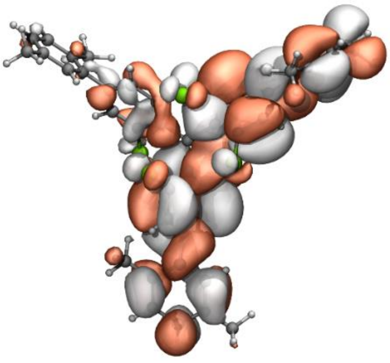 199a = –1.26 eV Occ.: 0	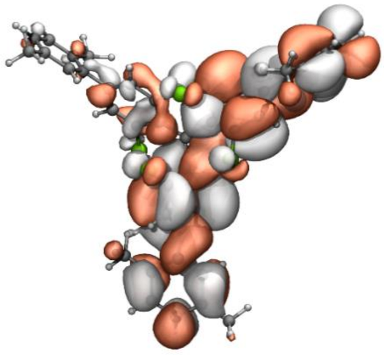 199b = –1.20 eV Occ.: 0
LUMO	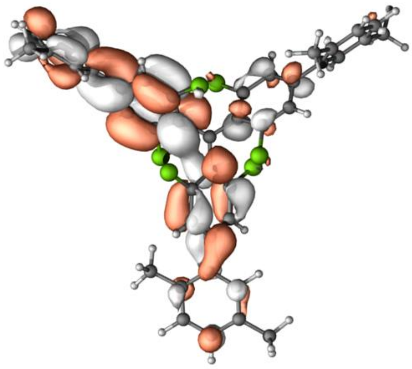 198a = –1.28 eV Occ.: 0	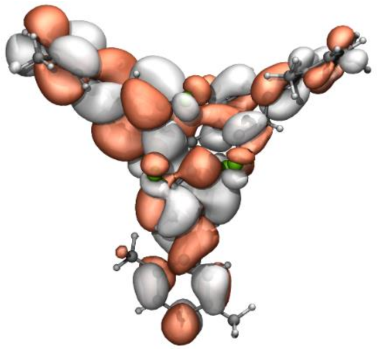 198b = –1.22 eV Occ.: 0
SOMO	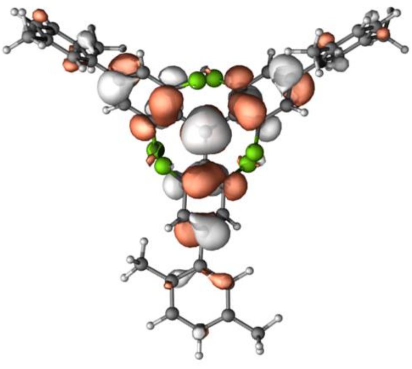 197a = –5.19 eV Occ.: 1	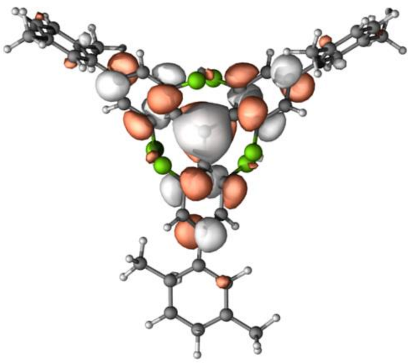 197b = –3.01 eV Occ.: 0

HOMO	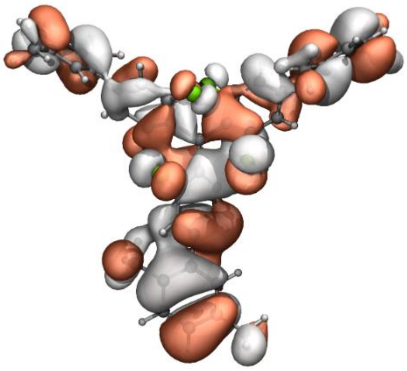<br>196a = –6.34 eV Occ.: 1	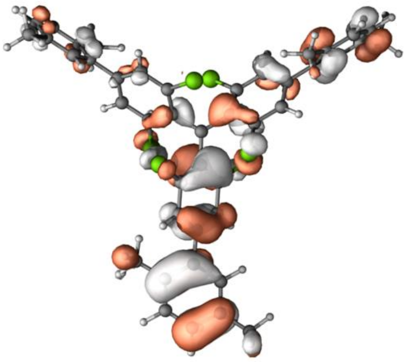<br>196b = –6.33 eV Occ.: 1
HOMO–1	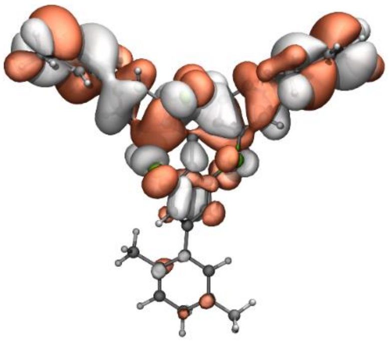<br>195a = –6.37 eV Occ.: 1	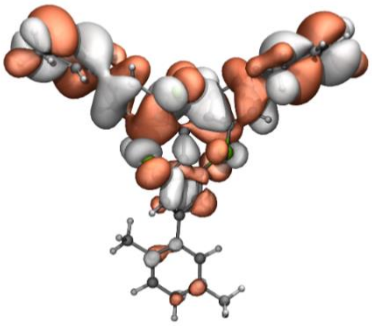<br>195b = –6.35 eV Occ.: 1
HOMO–2	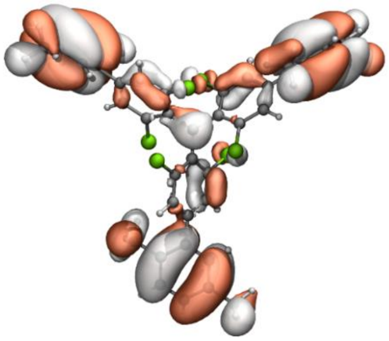<br>194a = –6.54 eV Occ.: 1	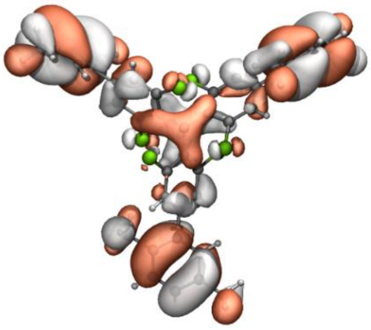<br>194b = –6.47 eV Occ.: 1
HOMO–3	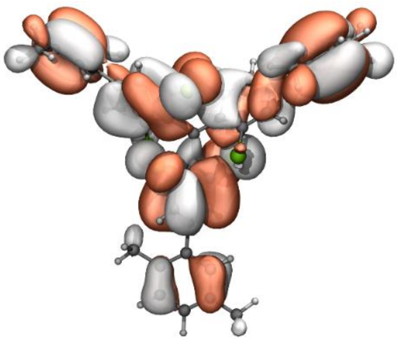<br>193a = –6.63 eV Occ.: 1	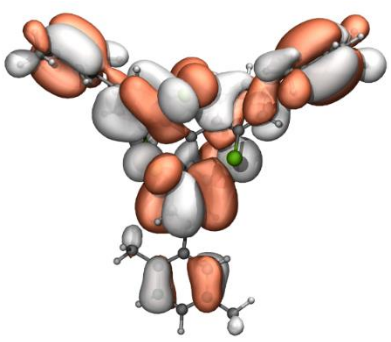<br>193b = –6.61 eV Occ.: 1

HOMO–4	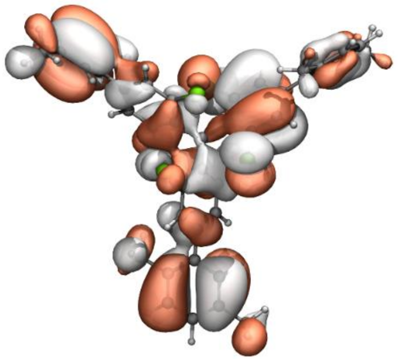 192a = –6.66 eV Occ.: 1	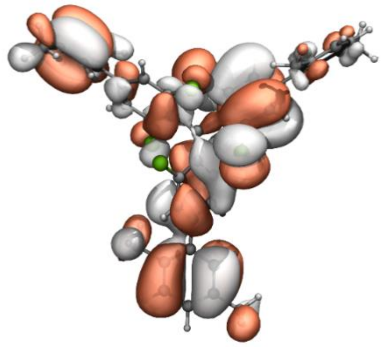 192b = –6.63 eV Occ.: 1

2,4-X$_3$TTM

	α	β
LUMO+1	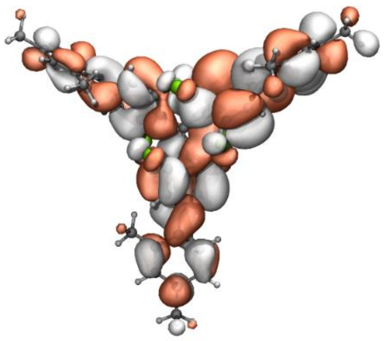 199a = –1.22 eV Occ.: 0	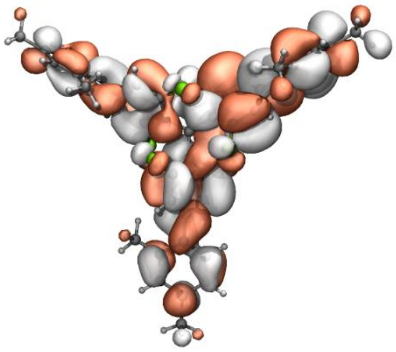 199b = –1.16 eV Occ.: 0
LUMO	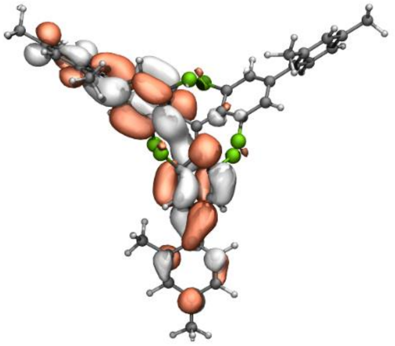 198a = –1.26 eV Occ.: 0	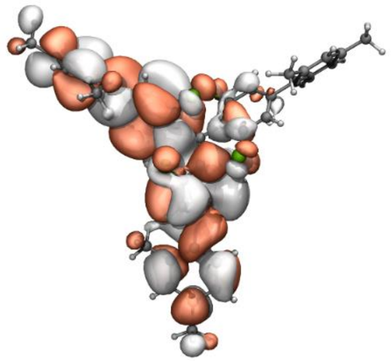 198b = –1.20 eV Occ.: 0
SOMO	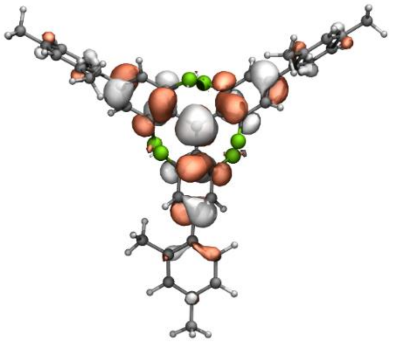 197a = –5.15 eV Occ.: 1	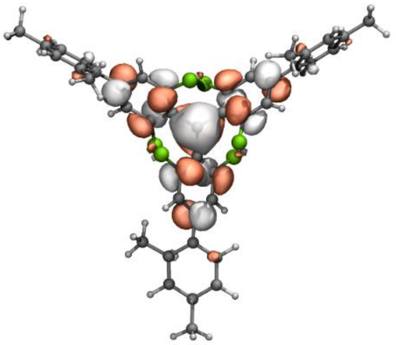 197b = –2.99 eV Occ.: 0

HOMO	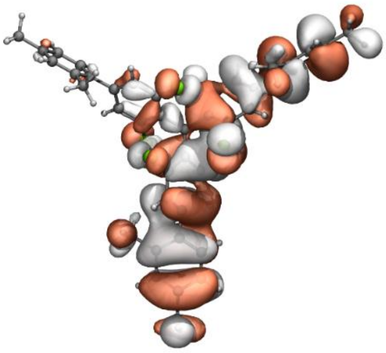 196a = –6.28 eV Occ.: 1	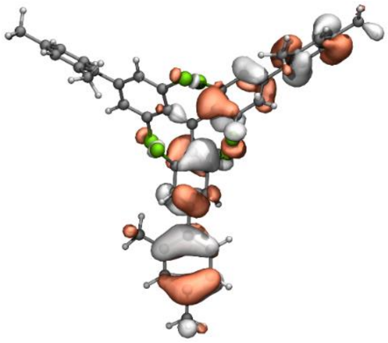 196b = –6.27 eV Occ.: 1
HOMO–1	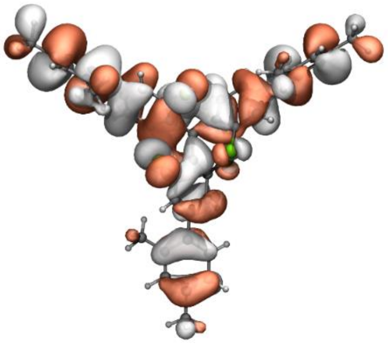 195a = –6.31 eV Occ.: 1	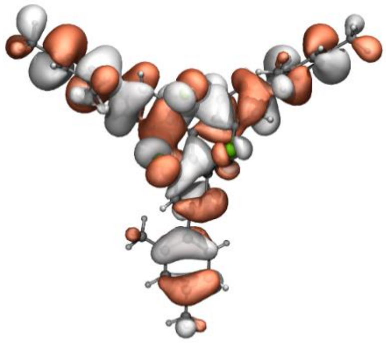 195b = –6.30 eV Occ.: 1

M$_3$TTM

	α	β
LUMO	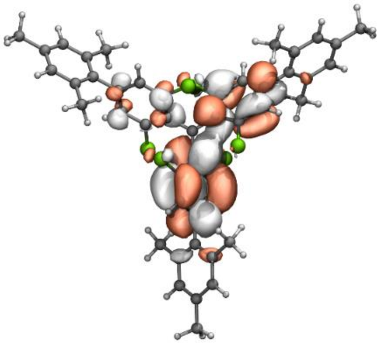 210a = –1.05 eV Occ.: 0	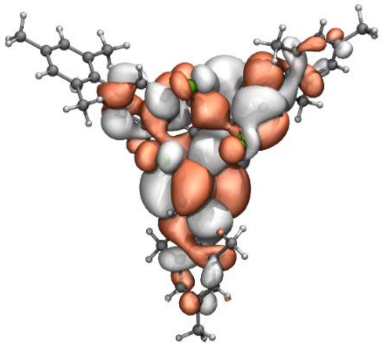 210b = –0.94 eV Occ.: 0
SOMO	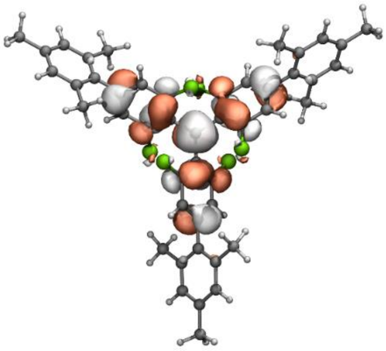 209a = –5.29 eV Occ.: 1	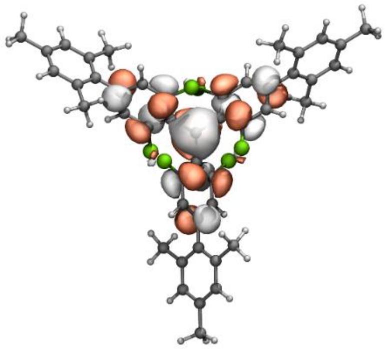 209b = –3.01 eV Occ.: 0

HOMO	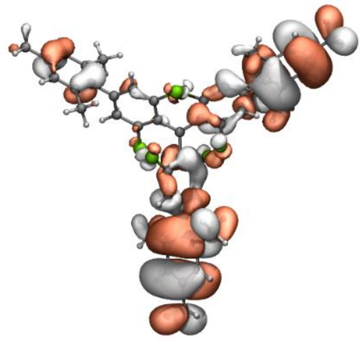<br>208a = –6.46 eV Occ.: 1	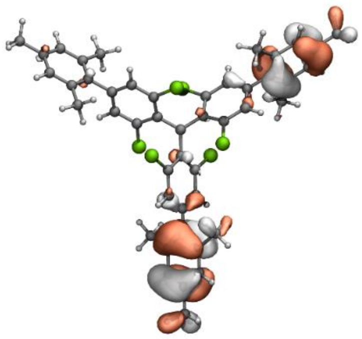<br>208b = –6.45 eV Occ.: 1
HOMO–1	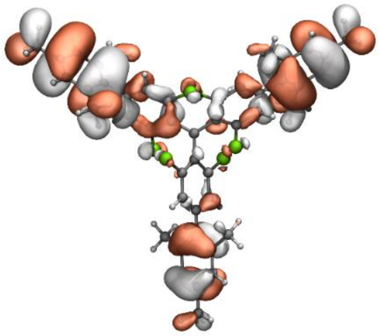<br>207a = –6.46 eV Occ.: 1	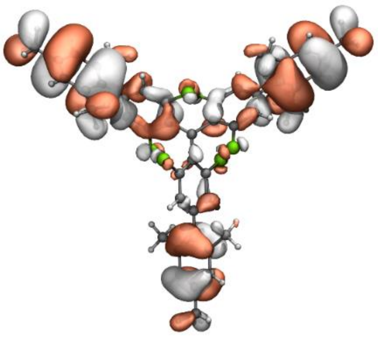<br>207b = –6.46 eV Occ.: 1
HOMO–2	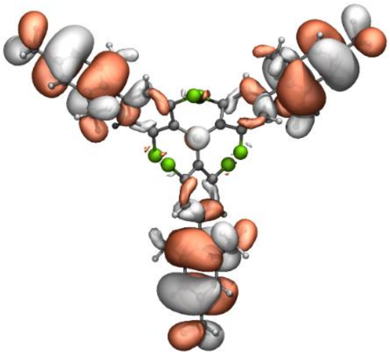<br>206a = –6.47 eV Occ.: 1	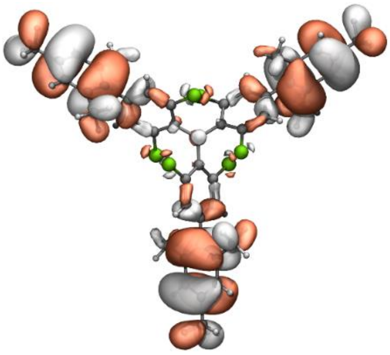<br>206b = –6.46 eV Occ.: 1
HOMO–3	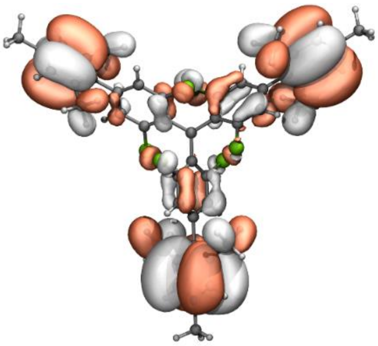<br>205a = –6.57 eV Occ.: 1	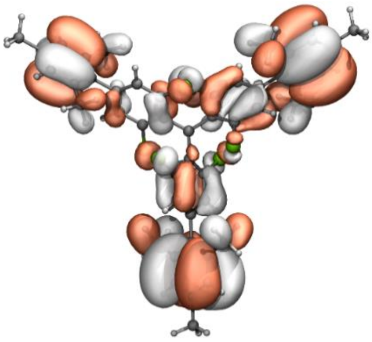<br>205b = –6.56 eV Occ.: 1

HOMO–4	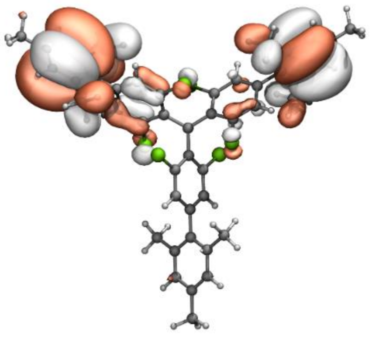 204a = –6.57 eV Occ.: 1	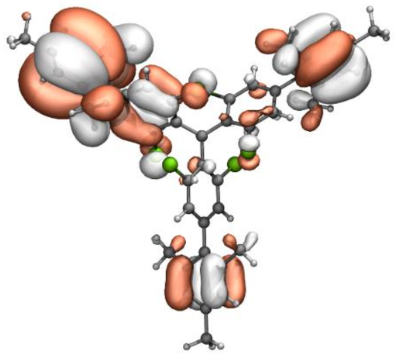 204b = –6.57 eV Occ.: 1
HOMO–5	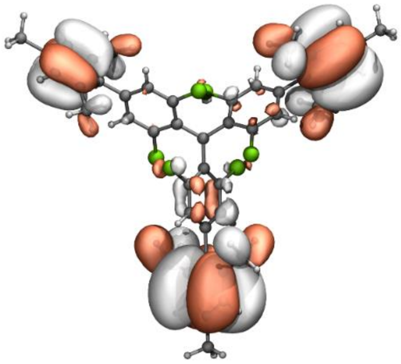 203a = –6.57 eV Occ.: 1	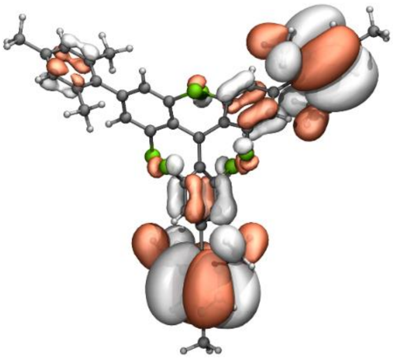 203b = –6.57 eV Occ.: 1
HOMO–6	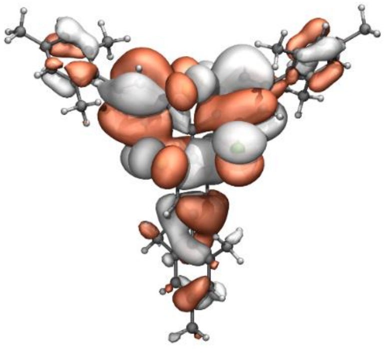 202a = –6.67 eV Occ.: 1	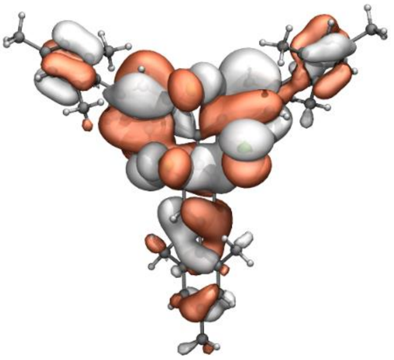 202b = –6.64 eV Occ.: 1
HOMO–7	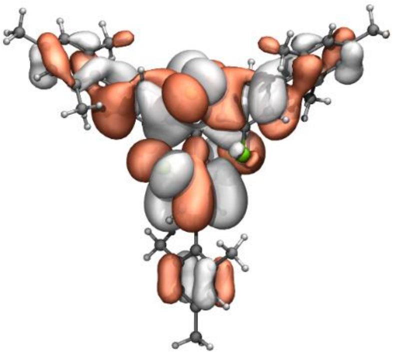 201a = –6.67 eV Occ.: 1	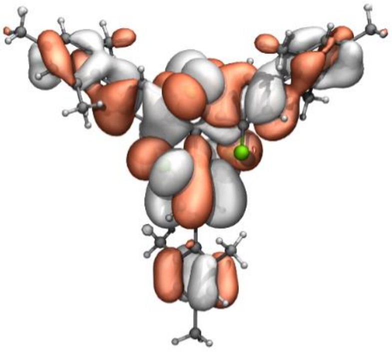 201b = –6.64 eV Occ.: 1

HOMO–8	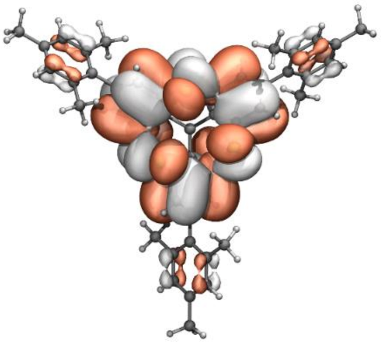 200a = –6.73 eV Occ.: 1	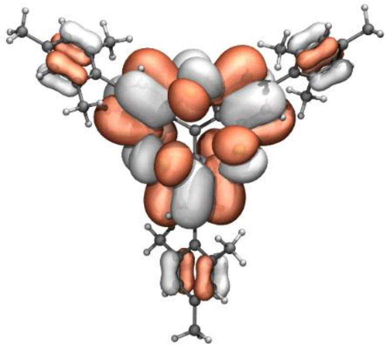 200b = –6.69 eV Occ.: 1
HOMO–9	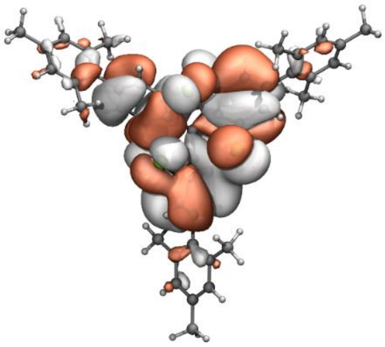 199a = –6.90 eV Occ.: 1	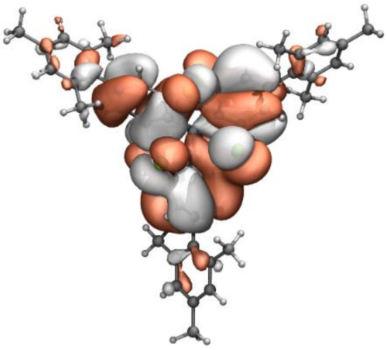 199b = –6.85 eV Occ.: 1
HOMO–10	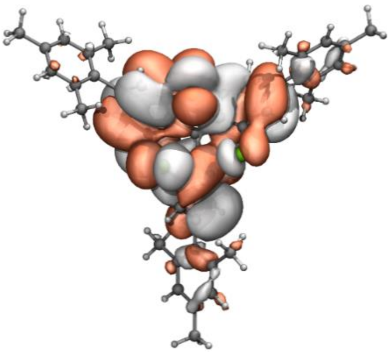 198a = –6.90 eV Occ.: 1	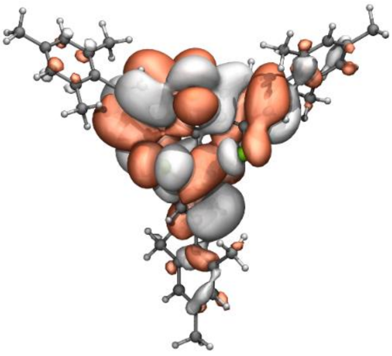 198a = –6.85 eV Occ.: 1

2,6-ipP$_3$TTM

	α	β
LUMO+1	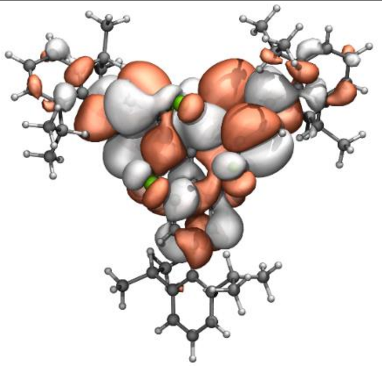 247a = –1.12 eV Occ.: 0	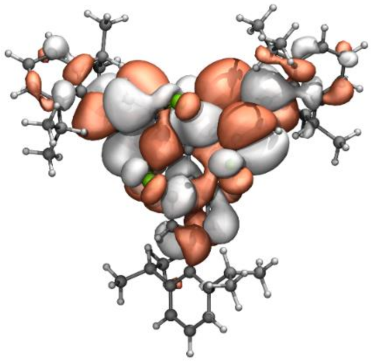 247b = –1.01 eV Occ.: 0

LUMO	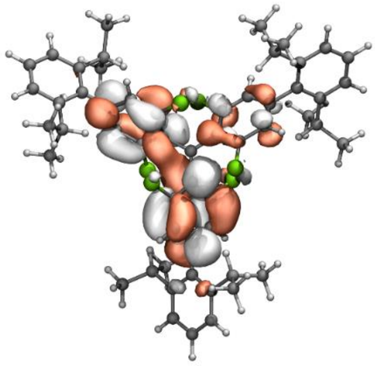 246a = –1.13 eV Occ.: 0	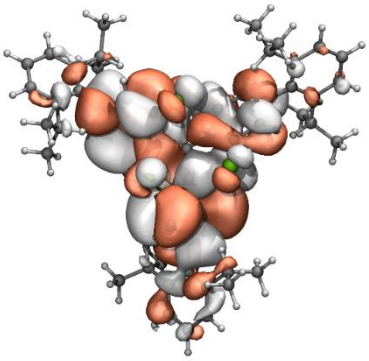 246b = –1.02 eV Occ.: 0
SOMO	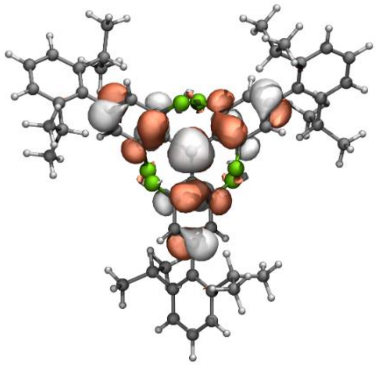 245a = –5.36 eV Occ.: 1	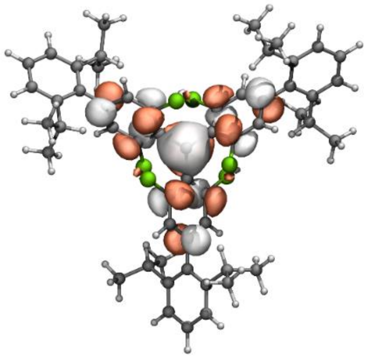 245b = –3.09 eV Occ.: 0
HOMO	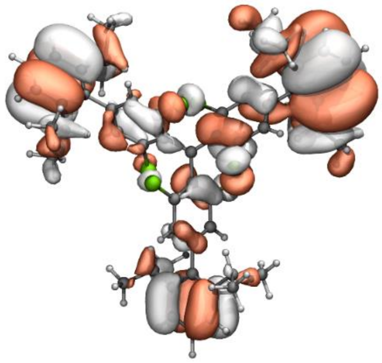 244a = –6.60 eV Occ.: 1	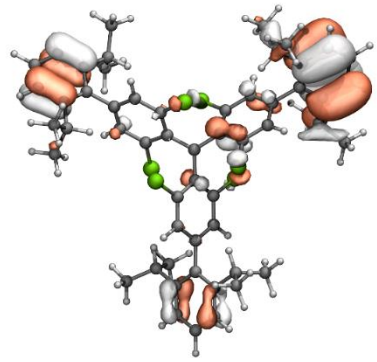 244b = –6.59 eV Occ.: 1
HOMO–1	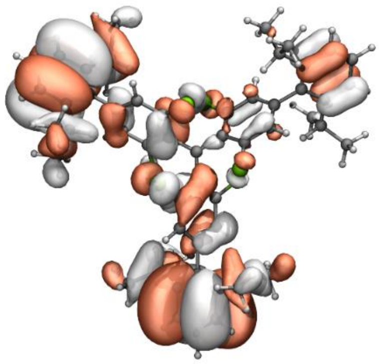 243a = –6.60 eV Occ.: 1	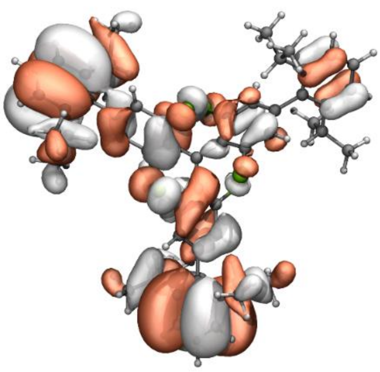 243b = –6.60 eV Occ.: 1

HOMO–2	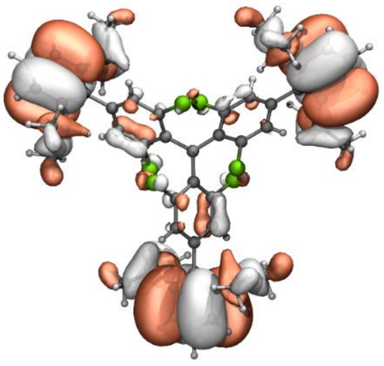 242a = –6.61 eV Occ.: 1	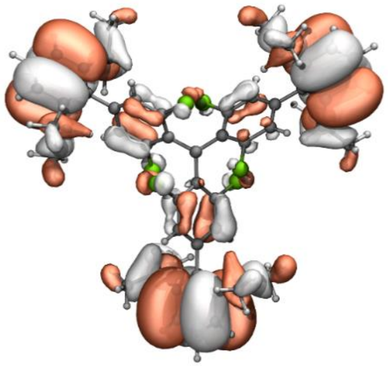 242b = –6.61 eV Occ.: 1
HOMO–3	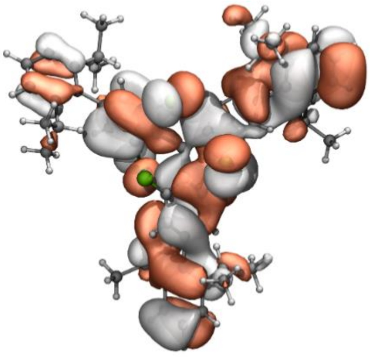 241a = –6.71 eV Occ.: 1	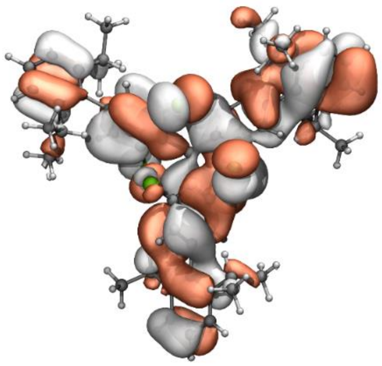 241b = –6.69 eV Occ.: 1
HOMO–4	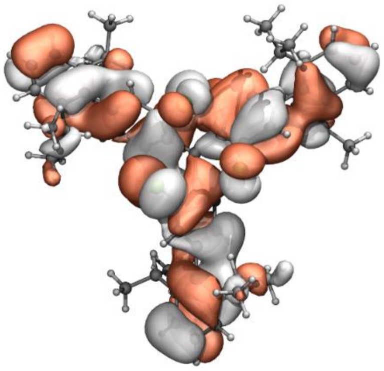 240a = –6.72 eV Occ.: 1	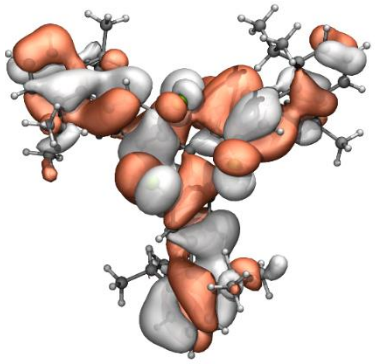 240b = –6.70 eV Occ.: 1
HOMO–5	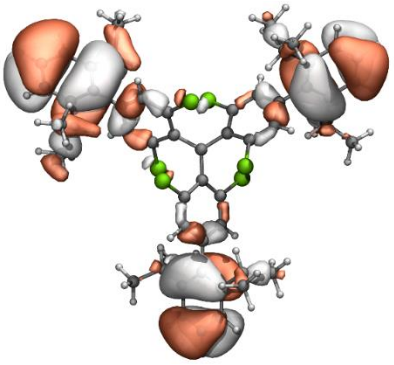 239a = –6.78 eV Occ.: 1	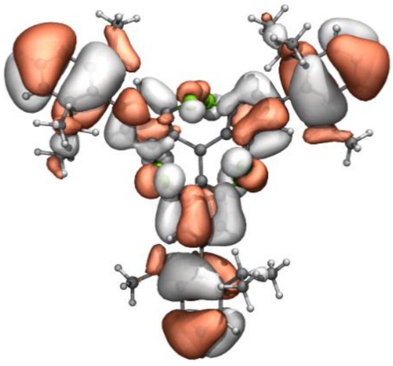 239b = –6.78 eV Occ.: 1

HOMO–6	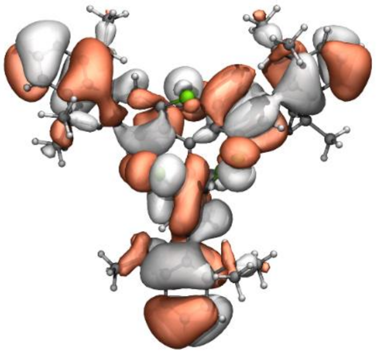 238a = –6.79 eV Occ.: 1	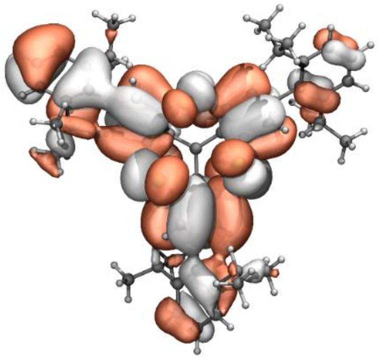 238b = –6.78 eV Occ.: 1
HOMO–7	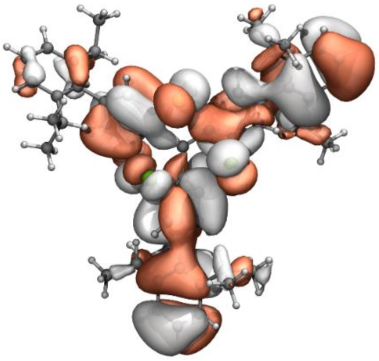 237a = –6.79 eV Occ.: 1	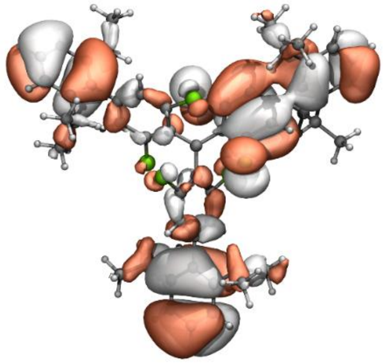 237b = –6.78 eV Occ.: 1
HOMO–8	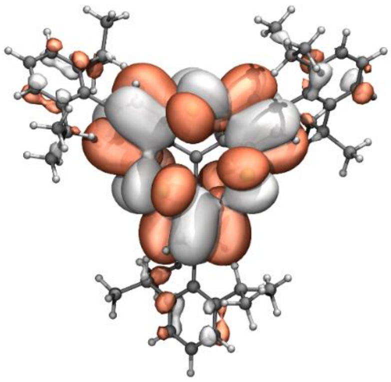 236a = –6.82 eV Occ.: 1	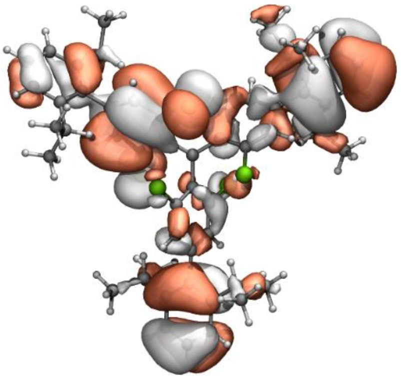 236b = –6.78 eV Occ.: 1
HOMO–9	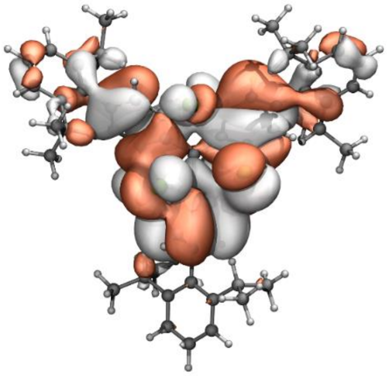 235a = –6.97 eV Occ.: 1	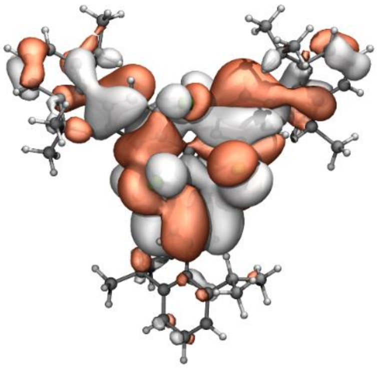 235b = –6.92 eV Occ.: 1

HOMO–10	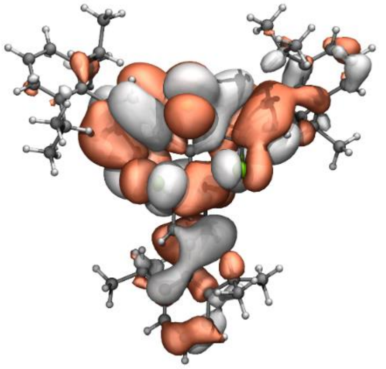<br>234a = –6.98 eV Occ.: 1	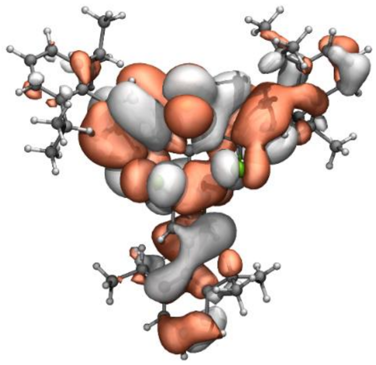<br>234a = –6.93 eV Occ.: 1

Electron spin density for optimized ground state geometries

	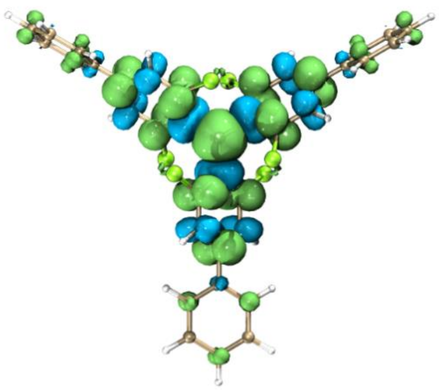<br>P_3TTM<br>SDI: 12.25	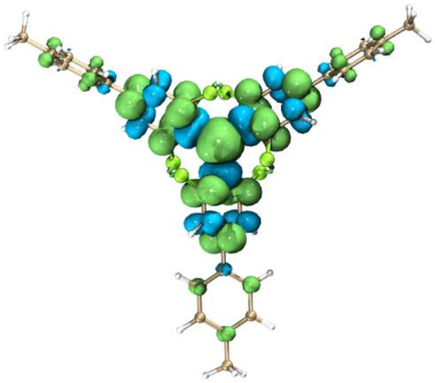<br>4-T_3TTM<br>SDI: 12.21
	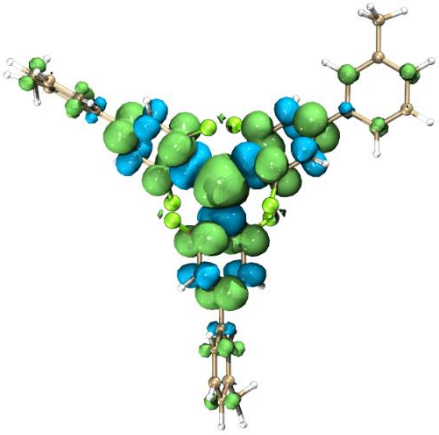<br>3-T_3TTM<br>SDI: 12.19	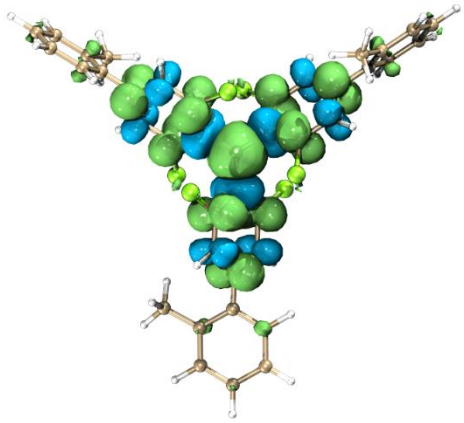<br>2-T_3TTM<br>SDI: 11.55

	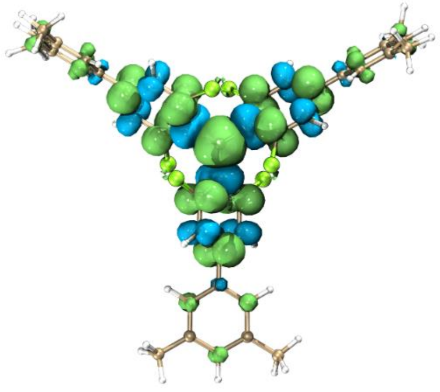<br>3,5-X$_3$TTM<br>SDI: 12.21	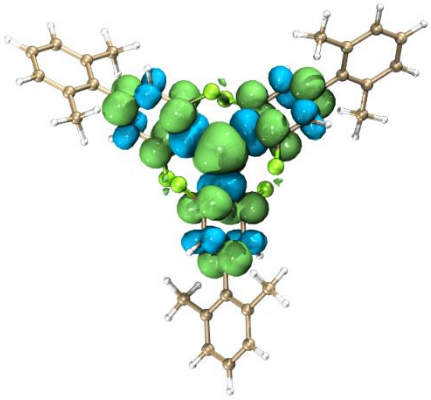<br>2,6-X$_3$TTM<br>SDI: 11.26
	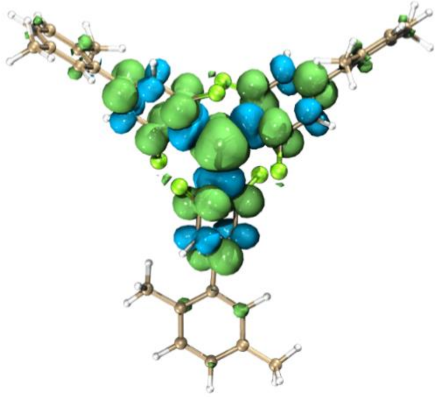<br>2,5-X$_3$TTM<br>SDI: 11.66	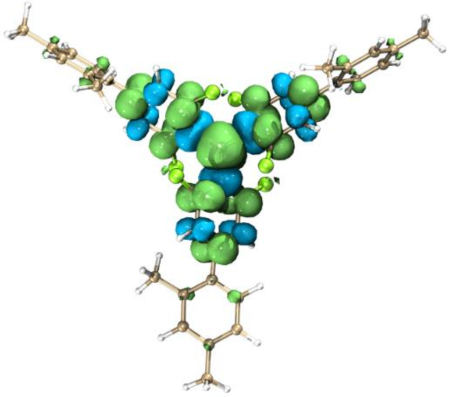<br>2,4-X$_3$TTM<br>SDI: 11.76
	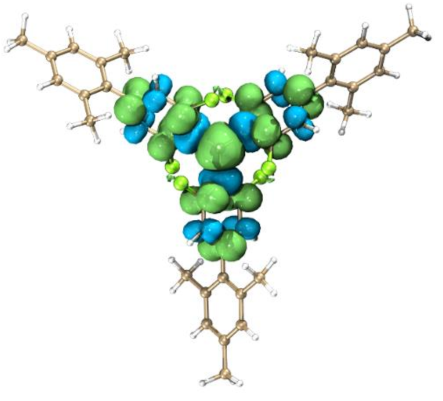<br>M$_3$TTM<br>SDI: 11.24	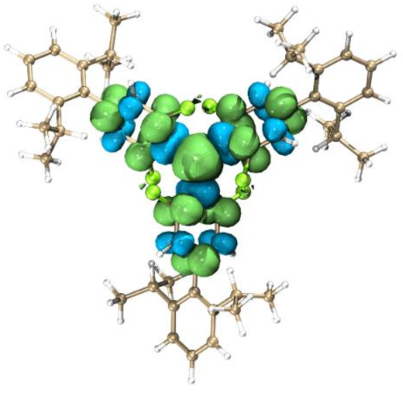<br>2,6-ipP$_3$TTM<br>SDI: 11.33

Hole-electron analysis for vertical and adiabatic excited states

P_3TTM

	D_1 vertical	D_1 adiabatic
Electron	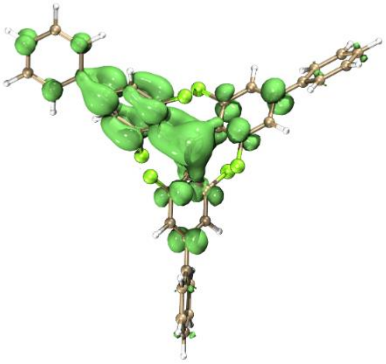<br>SOMOβ: 65.2%<br>LUMOα: 19.5%	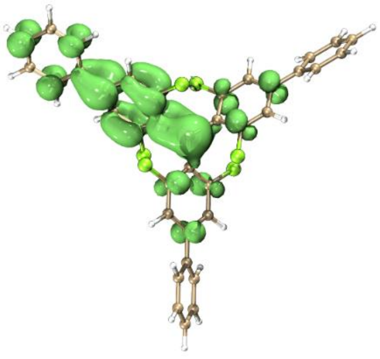<br>SOMOβ: 66.3%<br>LUMOα: 23.9%
Hole	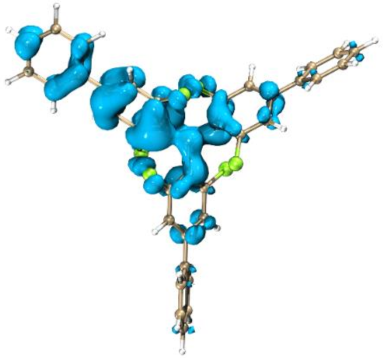<br>HOMOβ: 63.5%<br>SOMOα: 18.1%	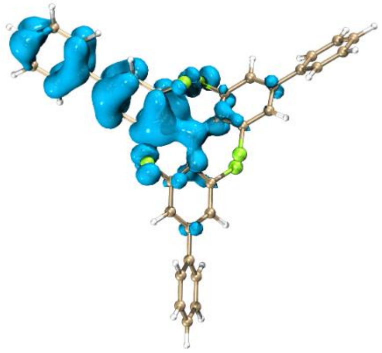<br>HOMOβ: 68.3%<br>SOMOα: 20.6%

4-T_3TTM

	D_1 vertical	D_1 adiabatic
Electron	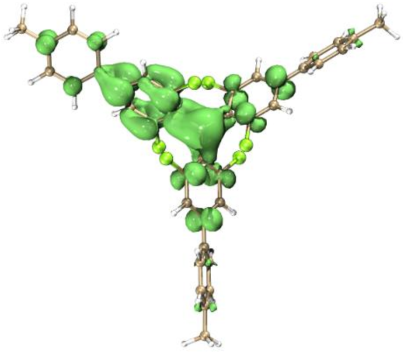<br>SOMOβ: 66.9%<br>LUMOα: 18.3%	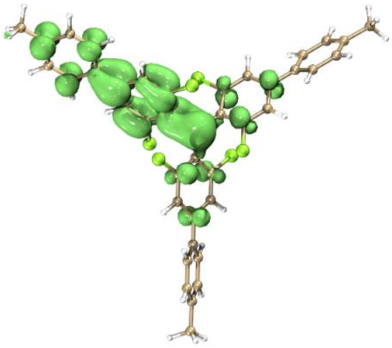<br>SOMOβ: 65.8%<br>LUMOα: 24.2%

Hole	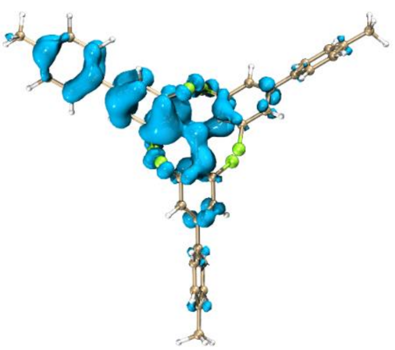<br>HOMOβ: 63.7%<br>SOMOα: 16.8%	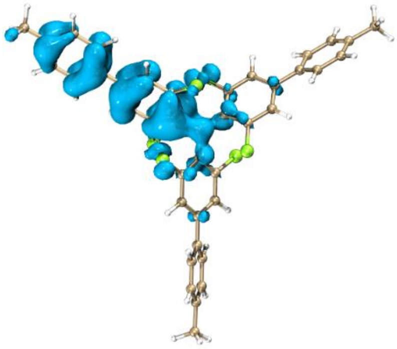<br>HOMOβ: 67.6%<br>SOMOα: 20.7%

3-T_3TTM

	D_1 vertical	D_1 adiabatic
Electron	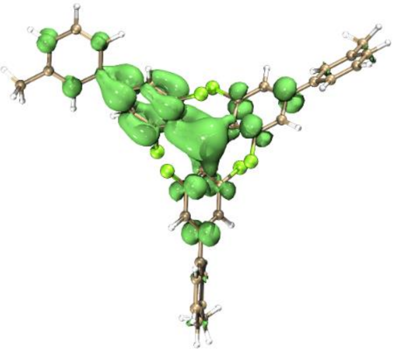<br>SOMOβ: 66.0%<br>LUMOα: 17.0%	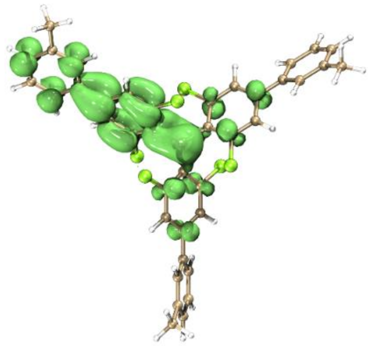<br>SOMOβ: 64.9%<br>LUMOα: 24.8%
Hole	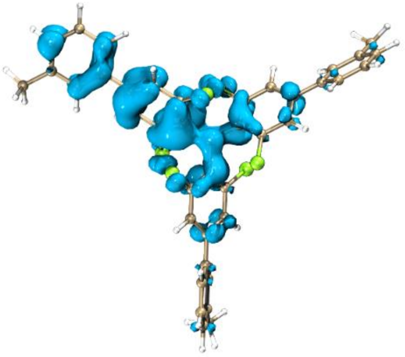<br>HOMOβ: 60.8%<br>SOMOα: 17.4%	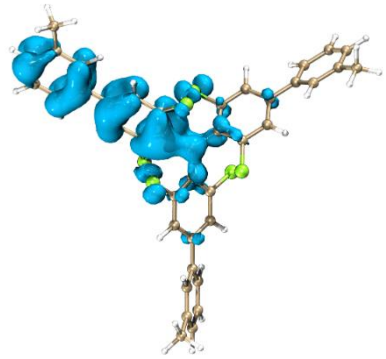<br>HOMOβ: 67.0%<br>SOMOα: 21.2%

2-T_3TTM

	D_1 vertical	D_1 adiabatic
Electron	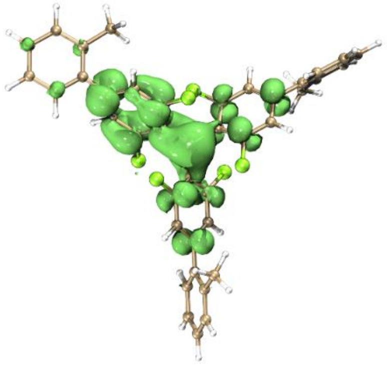<br>SOMOβ: 72.4%<br>LUMO+1α: 9.3%	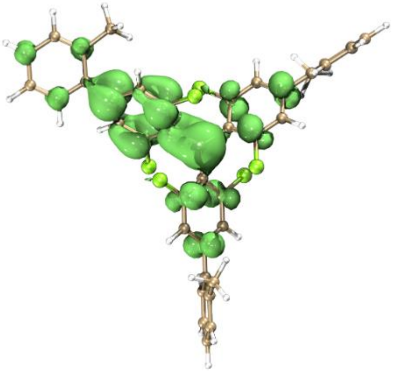<br>SOMOβ: 79.9%<br>LUMOα: 13.2%
Hole	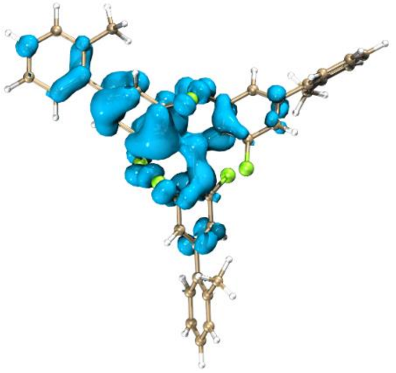<br>HOMOβ: 63.3%<br>SOMOα: 13.7%	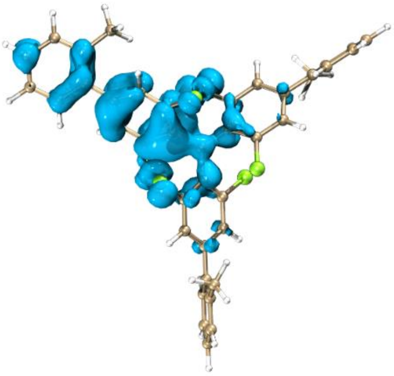<br>HOMOβ: 78.6%<br>SOMOα: 11.8%

3,5-X_3TTM

	D_1 vertical	D_1 adiabatic
Electron	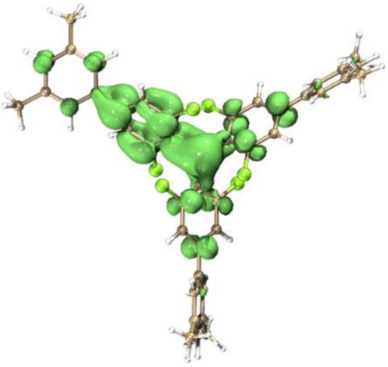<br>SOMOβ: 66.5%<br>LUMOα: 18.2%	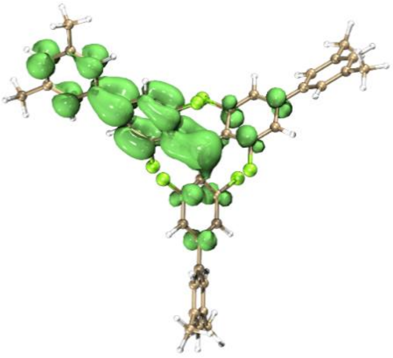<br>SOMOβ: 63.8%<br>LUMOα: 25.4%

Hole	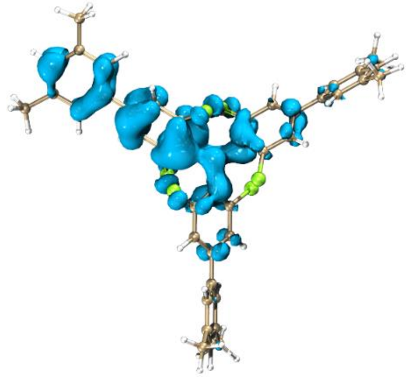<br>HOMOβ: 63.4%<br>SOMOα: 16.9%	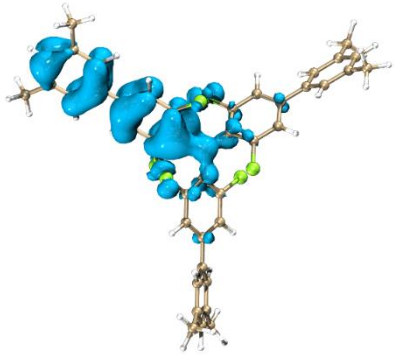<br>HOMOβ: 66.3%<br>SOMOα: 21.5%

2,6-X_3TTM

	D_1 vertical	D_1 adiabatic
Electron	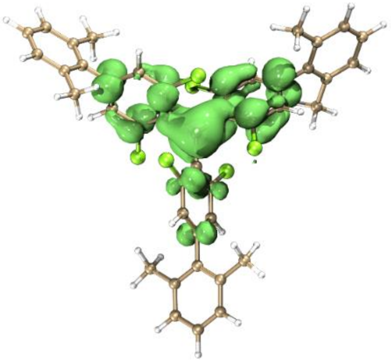<br>SOMOβ: 72.9%<br>LUMO+1α: 7.8%	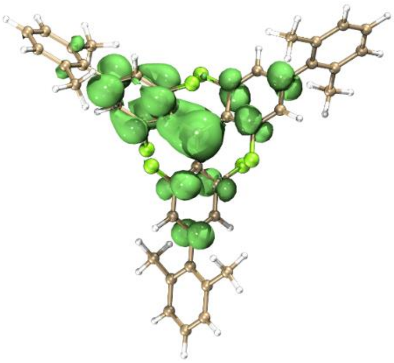<br>SOMOβ: 85.6%<br>LUMOα: 8.9%
Hole	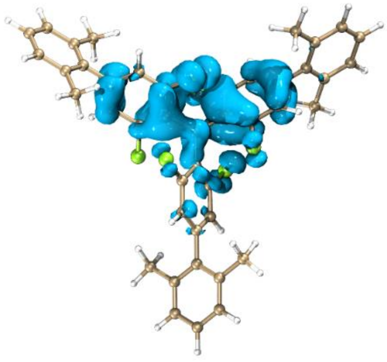<br>HOMO–3β: 35.3%<br>HOMO–7β: 13.3%<br>HOMO–9β: 18.2%<br>SOMOα: 12.7%	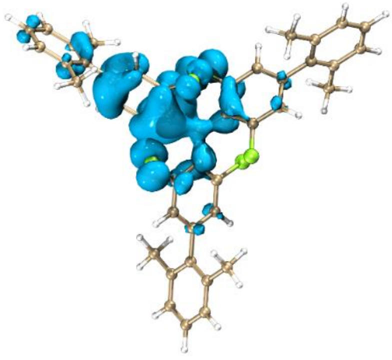<br>HOMOβ: 81.8%<br>SOMOα: 7.7%

2,5-X_3TTM

	D_1 vertical	D_1 adiabatic
Electron	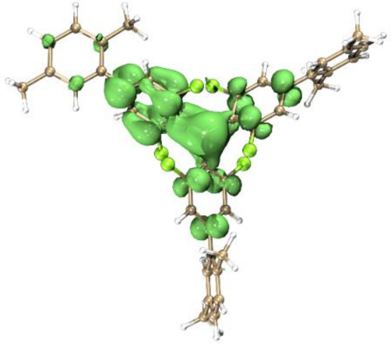<br>SOMOβ: 72.9%<br>LUMOα: 8.2%	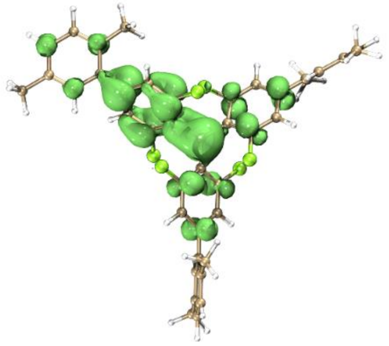<br>SOMOβ: 79.0%<br>LUMOα: 13.8%
Hole	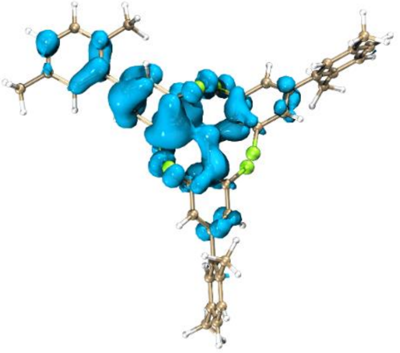<br>HOMOβ: 52.9%<br>HOMO–4β: 10.8%<br>SOMOα: 13.2%	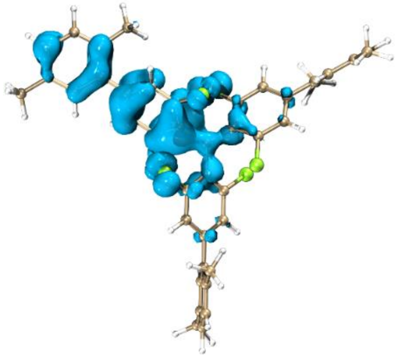<br>HOMOβ: 75.8%<br>SOMOα: 12.3%

2,4-X_3TTM

	D_1 vertical	D_1 adiabatic
Electron	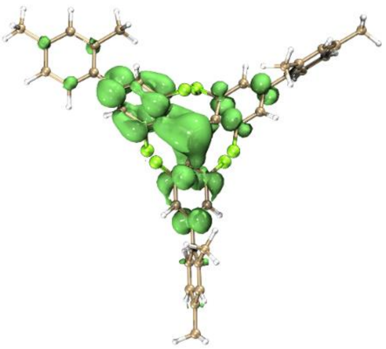<br>SOMOβ: 73.9%<br>LUMO+1α: 7.5%	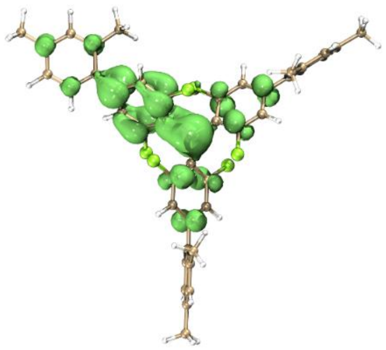<br>SOMOβ: 79.2%<br>LUMOα: 13.8%

Hole	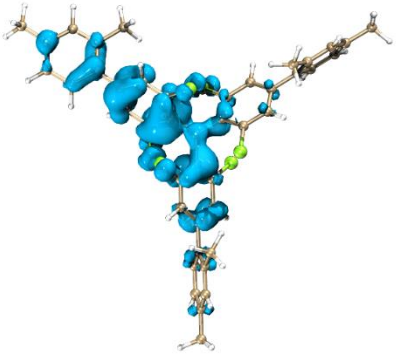<br>HOMOβ: 59.4%<br>SOMOα: 12.7%	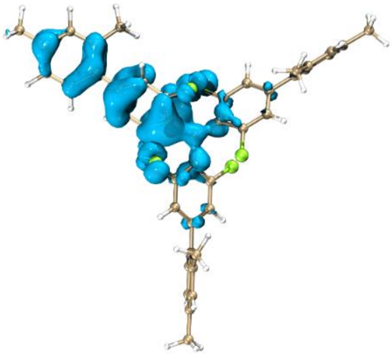<br>HOMOβ: 76.7%<br>SOMOα: 12.2%

M_3TTM

	D_1 vertical	D_1 adiabatic
Electron	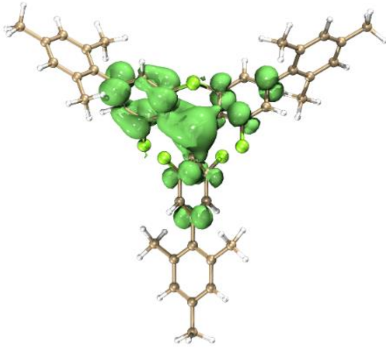<br>SOMOβ: 73.0%<br>LUMOα: 12.7%	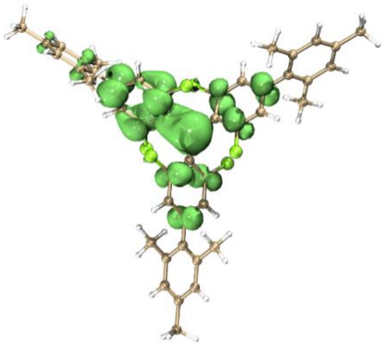<br>SOMOβ: 85.2%<br>LUMOα: 9.3%
Hole	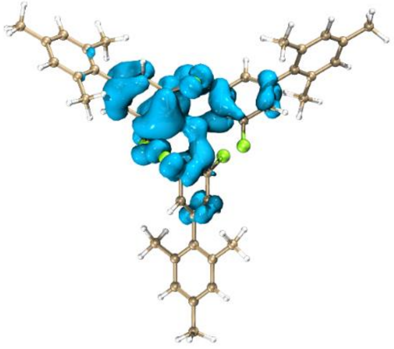<br>HOMO–6β: 47.1%<br>HOMO–10β: 16.4%<br>SOMOα: 12.7%	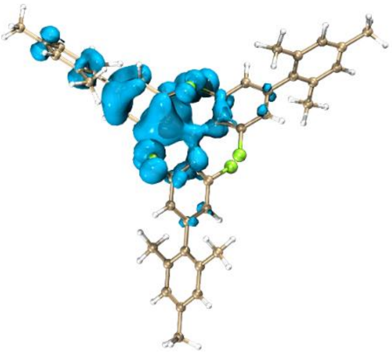<br>HOMOβ: 75.3%<br>SOMOα: 8.2%

2,6-ipP$_3$TTM

	D$_1$ vertical	D$_1$ adiabatic
Electron	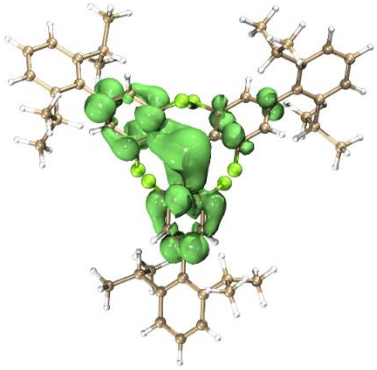<br>SOMOβ: 73.9%<br>LUMOα: 7.1%	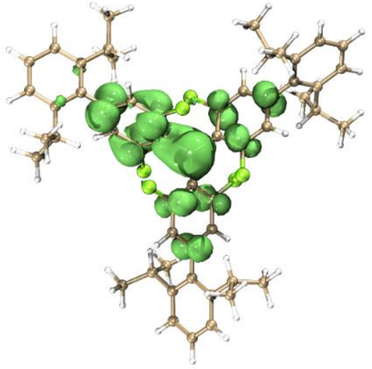<br>SOMOβ: 86.4%<br>LUMOα: 8.2%
Hole	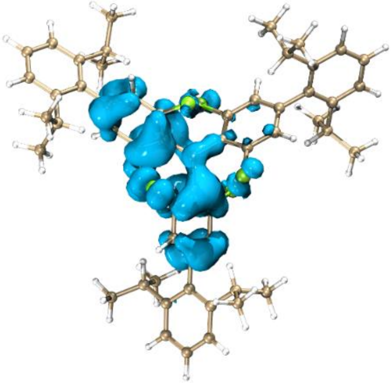<br>HOMO–3β: 34.8%<br>HOMO–7β: 16.3%<br>HOMO–10β: 12.7%<br>SOMOα: 12.4%	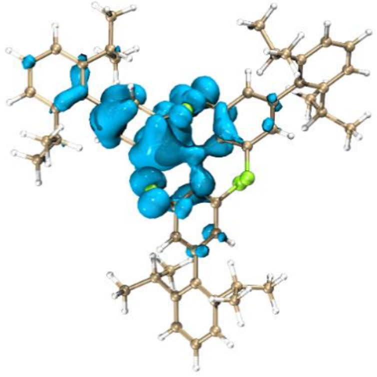<br>HOMOβ: 81.5%<br>SOMOα: 7.2%

S5. Cyclic Voltammetry

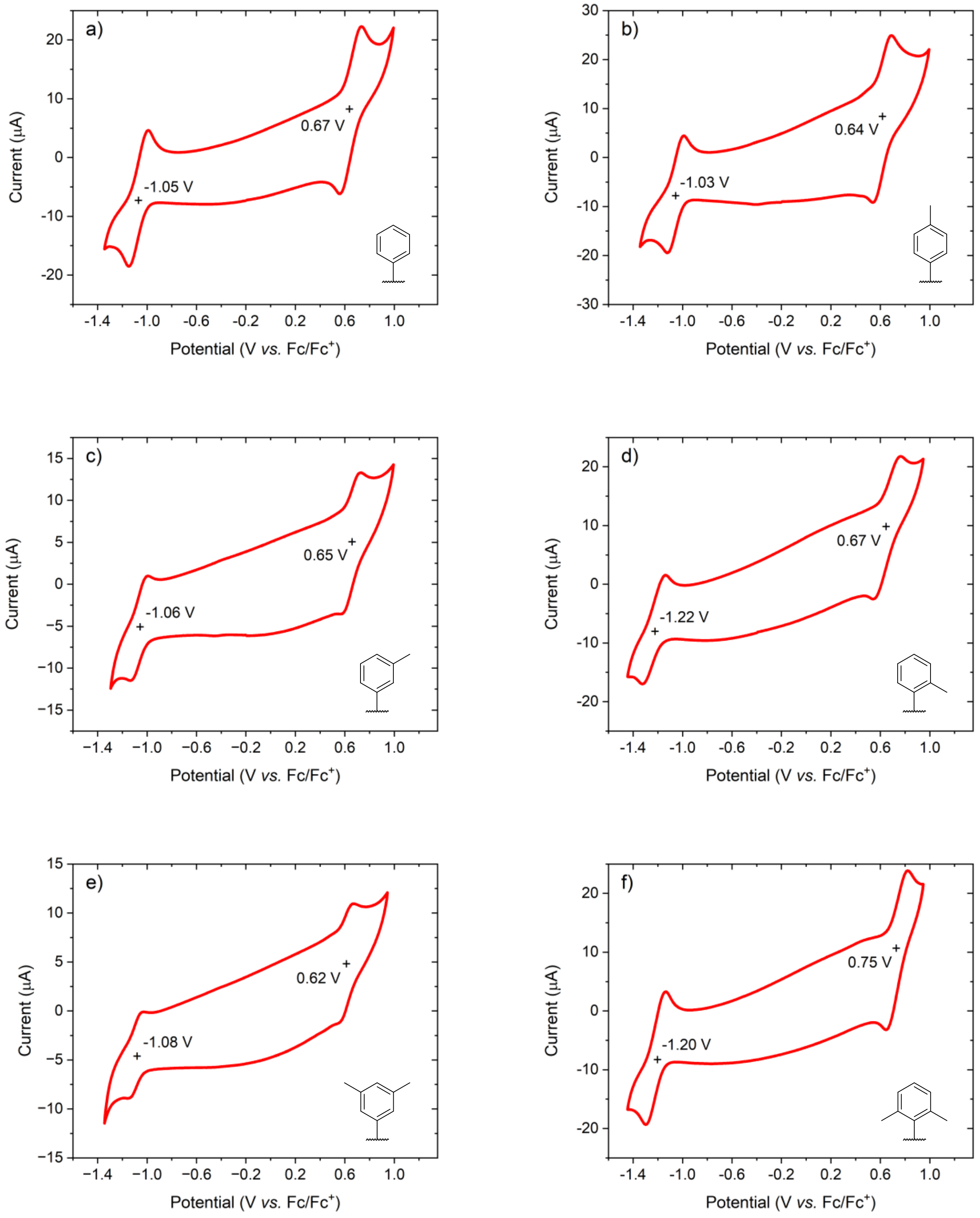

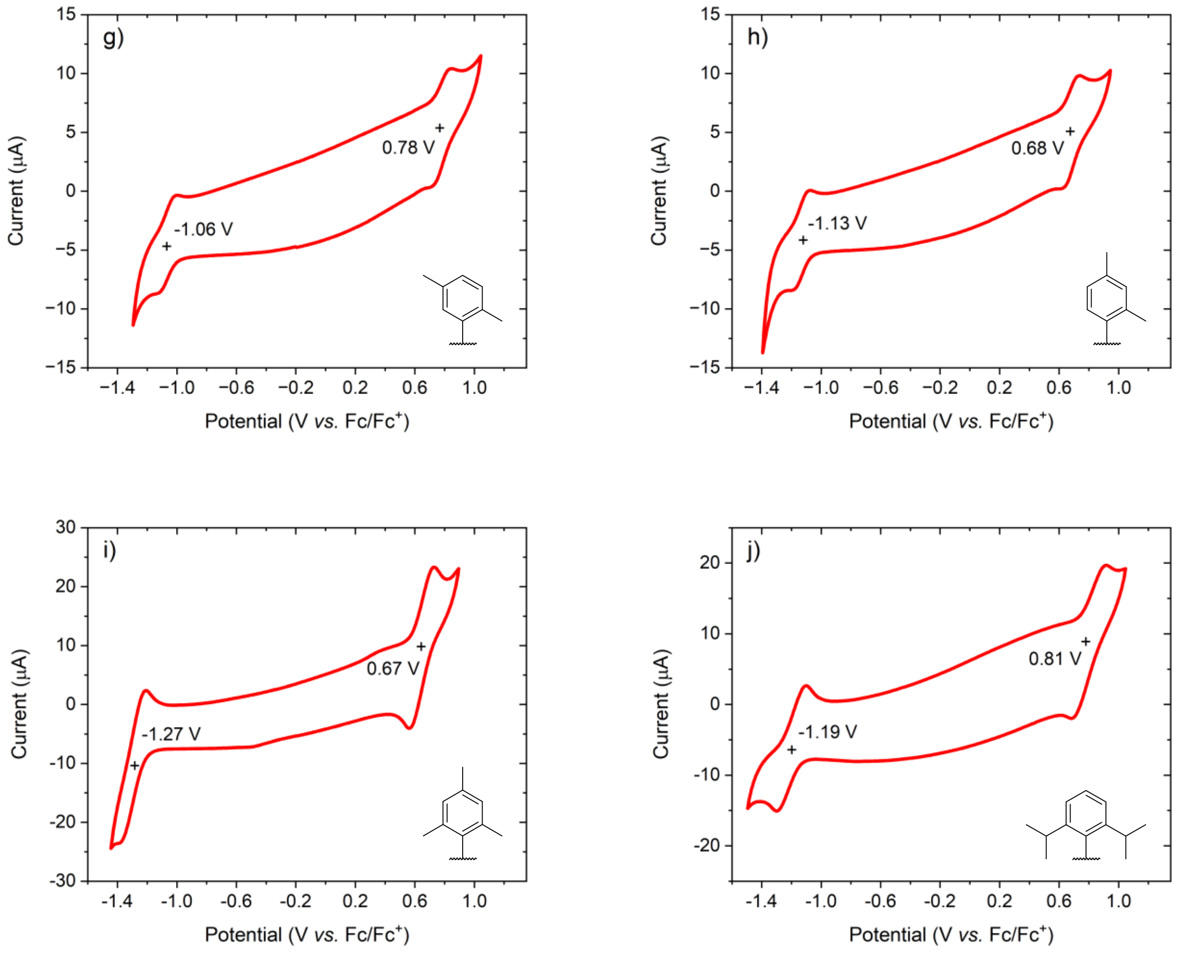

Figure S6. Cyclic voltammetry showing full reduction–oxidation cycles for a) P_3TTM, b) 4-T_3TTM, c) 3-T_3TTM, d) 2-T_3TTM, e) 3,5-X_3TTM, f) 2,6-X_3TTM, g) 2,5-X_3TTM, h) 2,4-X_3TTM, i) M_3TTM and j) 2,6-ipP_3TTM in 0.1 M solution of Bu_4NPF_6 in anhydrous THF at a scan rate of 0.1 V s^{-1}. The respective aryl groups are shown in the insets.

S6. X-Ray Crystallography

Refinement Details

P_3TTM. Crystals were thin needles and diffraction was weak. The data were integrated to 0.95 Å resolution, but I/σ(I) falls below 3 around 1.00 Å. The resulting R(int) value is high, and the precision of the structure is limited accordingly. The structure includes four independent molecules in the asymmetric unit, which show some variation of the dihedrals between the best planes of the phenyl rings, spanning the range 21.4–53.4° (Table S8).

4-T_3TTM. The structure is commensurately modulated. The average structure can be represented in space group C2/c in a unit cell with 1/5th of the volume (a = 14.3, b = 18.8, c = 14.3, β = 119.1°), with half a molecule in the asymmetric unit (on a 2-fold axis). In this description, the displacement ellipsoids of all molecules are stretched along the *b* axis, indicative of some deficiency with the model. The supercell structure (as in Table S6) shows a modulation where the molecules related by approximate translation are displaced subtly along the *b* axis:

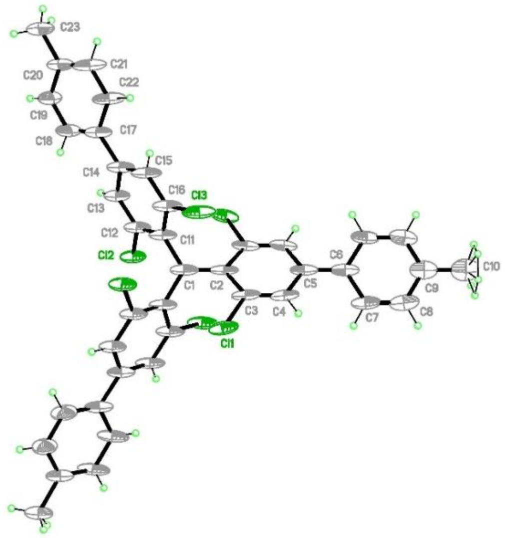

4-T_3TTM molecule refined in the crystallographic subcell: displacement ellipsoids are stretched along the *b* axis (horizontal).

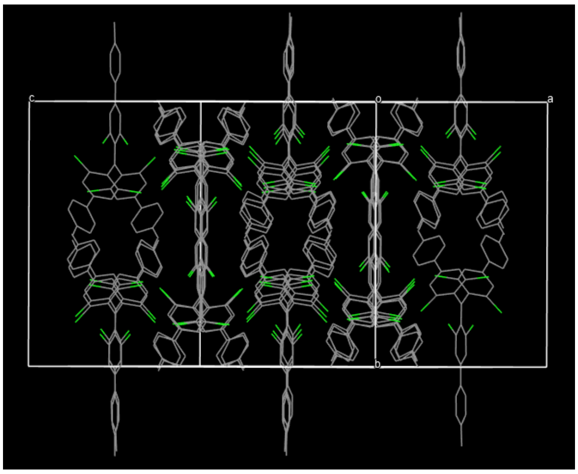

Supercell description, viewed along the direction of approximate, showing displacement of the molecules along the *b* axis (vertical).

The supercell description is considered to be the best overall description of the structure. This model includes three molecules in the asymmetric unit, one of which is situated on a crystallographic 2-fold axis, and two of which are situated on general positions ($Z' = 2.5$). The molecules show some geometrical variation, with the dihedrals between the best planes of the phenyl rings spanning the range 10.0–34.0° (Table S8).

3-T$_3$TTM. Although the crystal structure is non-centrosymmetric, the refined value of the Flack parameter is consistent with inversion twinning. The final refinement was carried out on this basis, with "TWIN/BASF" commands in *SHELXL*. The structure includes two molecules in the asymmetric unit, one of which is situated on a crystallographic 2-fold axis, and one of which is situated on a general position ($Z' = 1.5$). In both molecules, two of the methylphenyl rings appear ordered while the third shows two orientations related by a 2-fold rotation. For the molecule on the crystallographic 2-fold axis, the disorder arises around the 2-fold and is therefore implied to be 50:50. The other molecule shows a major (ca. 85%) and a minor (ca. 15%) disorder component. The dihedrals between the best planes of the phenyl rings span the range 24.6–51.3° (Table S8), which is comparable to the range seen in P$_3$TTM.

2-T$_3$TTM. The structure includes one molecule on a crystallographic general position, with two methylphenyl rings appearing ordered and one showing 2-fold disorder with approximately 50:50 occupancy. The geometry around the disordered methylphenyl ring is distorted, showing an apparent bending at the central C–C bond. This may result in part from approximations associated with modelling disorder in this region or it may reflect a genuine distortion of the molecule in the crystal. The X-ray analysis was repeated for two crystals from different crystallization batches and the results were the same. The same distortion is seen to a lesser extent for the minor disorder component in 3-T$_3$TTM.

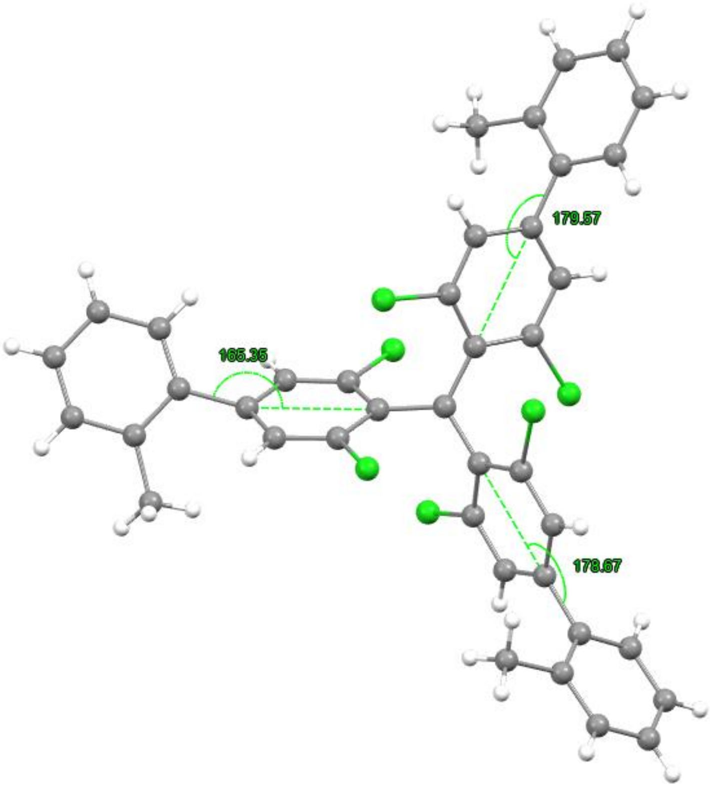

Apparent bending of the central C–C bond for the disordered 2-methylphenyl ring in 2-T$_3$TTM. Only one component is shown; the other disorder component shows a similar bend “downwards” (see Figure 4a in the main manuscript).

3,5-X$_3$TTM. The structure includes one molecule situated on a crystallographic 2-fold axis (Z' = 0.5), consistent with true C_2 point symmetry for the molecule. The dihedrals between the best planes of the phenyl rings span a narrow range 29.4–30.6° (Table S8).

2,6-X$_3$TTM. The crystals contain DCM solvent molecules. One molecular site is clear in the asymmetric unit (around 0.68, 0.73, 0.86) giving two DCM per formula unit and 8 per unit cell. Multiple partially occupied molecules are required to model this DCM, and a better result is obtained by applying the *SQUEEZE* algorithm. *SQUEEZE* corrects for 334 electrons per unit cell, consistent with the anticipated 8 × DCM (8 × 42 = 336). Application of *SQUEEZE* lowers *wR*2 by ca. 10% and improves the precision of the main molecule significantly. The structure includes one 2,6-X$_3$TTM

molecule situated on a crystallographic 2-fold axis ($Z' = 0.5$), consistent with true C_2 point symmetry. The presence of two *ortho*-methyl groups yields consistently large dihedrals between the best planes of the phenyl rings: 72.8–84.1° (Table S8).

2,4-X$_3$TTM. The structure includes one molecule on a crystallographic 3-fold axis and one molecule on a site of 32 point symmetry (D_3). The latter molecule shows disorder of each dimethylphenyl ring around the crystallographic 2-fold axis, but with the phenyl ring displaced from the 2-fold axis in a manner similar to that illustrated above for 3-T$_3$TTM and 2-T$_3$TTM. As for 3-T$_3$TTM and 2-T$_3$TTM, the apparent bending at the central C–C bond may be in part due to approximations modelling the disorder in this region, but the fact that it is seen in three different structures suggests it is probably a real observation, by which the molecular geometry is distorted by crystal packing effects. In this structure, the 2,4-X$_3$TTM molecules are arranged in approximately planar layers perpendicular to the 3-fold axis, and the disorder occurs specifically in every third layer. The dihedrals between the best planes of the phenyl rings are quite different for the two crystallographically distinct molecules, being 64.4° for the molecule on the 3-fold axis and 37.6° for the disordered molecule (Table S8).

M$_3$TTM. The crystal structure has been published previously (CCDC 2195366).[1] The structure includes one molecule on a crystallographic 2-fold axis, with dihedrals between the best planes of the phenyl rings spanning the range 81.7–87.8° (Table S8).

2,6-ipP$_3$TTM. The structure includes one molecule on a crystallographic general position, with the dihedrals between the best planes of the phenyl rings spanning the range 70.0–89.0° (Table S8).

Attempted structure determination for 2,5-X$_3$TTM. Several 2,5-X$_3$TTM crystals were examined from different crystallization batches. The following simulated precession images are representative

results. *hkl* indices refer to the unit cell: *a* = 9.84, *b* = 9.86, *c* = 9.85 Å, α = 90.0, β = 90.2, γ = 88.9°. Although the *h*0*l* and *h*1*l* images appear to show sharp Bragg peaks, the smearing of the peaks in the other images reveal imperfections in these crystals. To date, it has not been possible to establish a structure from these diffraction patterns.

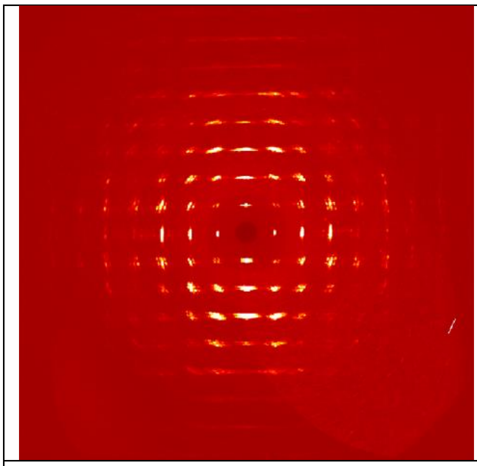	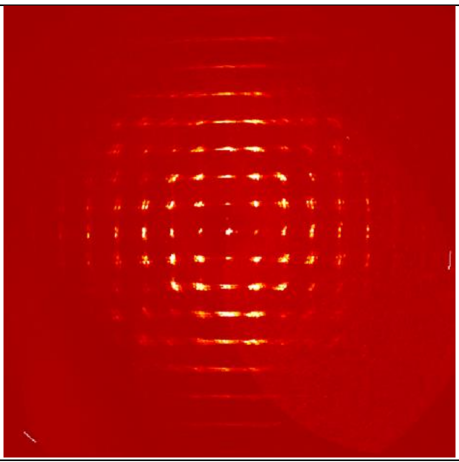
**0*kl***	**1*kl***
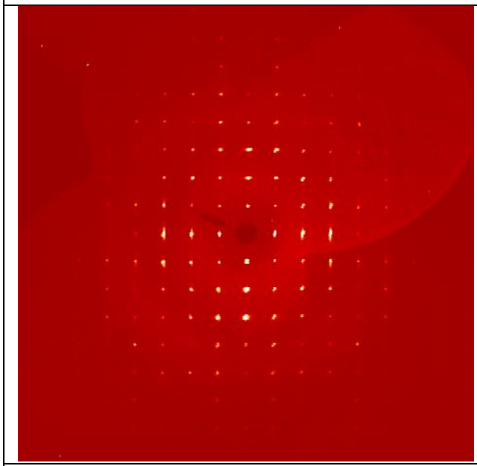	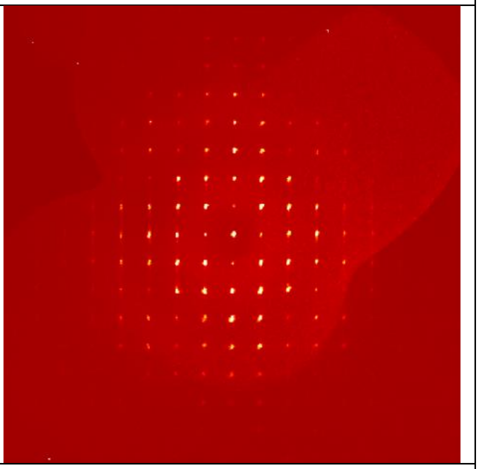
h*0*l	***h*1*l***
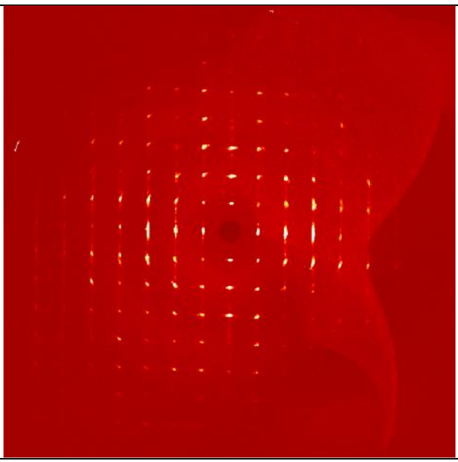	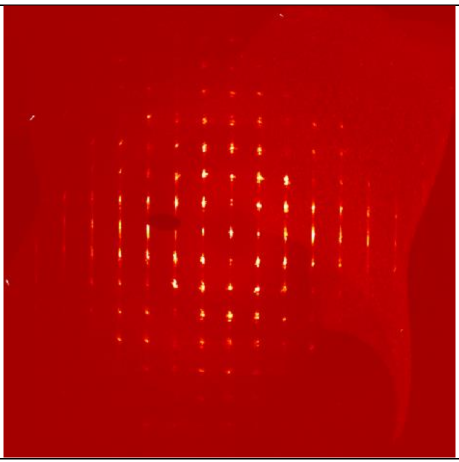
***hk*0**	***hk*1**

Table S6. Experimental and crystal refinement parameters

	P_3TTM	4-T_3TTM	3-T_3TTM [a]	2-T_3TTM
CCDC number	2305974	2305973	2305968	2305970
Cambridge data ID	HB_B2_0046	HB_B1_0078	HB_B1_0084	HB_B2_0045
Chemical formula	$C_{37}H_{21}Cl_6$	$C_{40}H_{27}Cl_6$	$C_{40}H_{27}Cl_6$	$C_{40}H_{27}Cl_6$
Formula weight	678.24	720.31	720.31	720.31
Temperature / K	180(2)	180(2)	180(2)	180(2)
Crystal system	triclinic	monoclinic	monoclinic	orthorhombic
Space group	P –1	C 2/c	I 2	P b c a
a / Å	12.9954(7)	38.301(2)	19.0105(13)	18.3082(12)
b / Å	17.4139(9)	18.8493(12)	14.0367(6)	14.0332(9)
c / Å	29.7775(16)	24.6749(14)	19.1590(8)	26.9220(17)
α / °	80.442(4)	90	90	90
β / °	85.743(4)	108.749(2)	94.7460(13)	90
γ / °	69.774(3)	90	90	90
Unit-cell volume / $Å^3$	6234.3(6)	16868.7(17)	5094.9(5)	6916.9(8)
Z	8	20	6	8
Calc. density / g cm^{-3}	1.445	1.418	1.409	1.383
F(000)	2760	7380	2214	2952
Radiation type	Cu Kα	Cu Kα	Cu Kα	Cu Kα
Absorption coefficient / mm^{-1}	5.233	4.867	4.835	4.748
Crystal size / mm^3	0.30×0.02×0.01	0.12×0.12×0.04	0.14×0.14×0.02	0.26×0.12×0.06
2θ range / °	5.47–109.02	4.87–134.44	9.26–133.33	6.57–133.16
Completeness to max 2θ	0.994	0.992	0.993	0.997
No. of reflections measured	90858	154448	29024	65185
No. of independent reflections	15305	14995	8804	6093
R(int.)	0.2002	0.1410	0.0342	0.0403
No. parameters / restraints	1550 / 0	1047 / 0	641 / 1	483 / 0
Final R1 values (I > 2s(I))	0.0567	0.0793	0.0264	0.0259
Final wR(F^2) values (all data)	0.1550	0.2573	0.0678	0.0680
Goodness-of-fit on F^2	0.988	1.071	1.036	1.040
Largest diff. peak & hole / e $Å^{-3}$	0.418, –0.389	0.722, –0.867	0.398, –0.185	0.210, –0.314
Flack parameter			0.457(10) [a]	

[a] Refined as an inversion twin.

Table S7. Experimental and crystal refinement parameters

	3,5-X_3TTM	2,6-X_3TTM [a]	2,4-X_3TTM	2,6-ipP_3TTM
CCDC number	2305971	2305967	2305969	2305972
Cambridge data ID	HB_B1_0085	HB_B1_0065	HB_B1_0092	HB_B1_0079
Chemical formula	$C_{43}H_{33}Cl_6$	$C_{43}H_{33}Cl_6$ [+solv]	$C_{43}H_{33}Cl_6$	$C_{55}H_{57}Cl_6$
Formula weight	762.39	762.39	762.39	930.70
Temperature / K	180(2)	180(2)	180(2)	180(2)
Crystal system	monoclinic	monoclinic	trigonal	monoclinic
Space group	C 2/c	C 2/c	P –3 c 1	P 21/n
a / Å	11.6930(4)	24.3544(8)	15.5401(3)	13.5094(4)
b / Å	19.0547(7)	13.6294(5)	15.5401(3)	17.0165(5)
c / Å	16.8545(6)	13.8120(5)	27.4732(10)	22.2151(6)
α / °	90	90	90	90
β / °	103.156(2)	107.1114(13)	90	95.3271(14)
γ / °	90	90	120	90
Unit-cell volume / $Å^3$	3656.7(2)	4381.8(3)	5745.8(3)	5084.8(3)
Z	4	4	6	4
Calc. density / g cm^{-3}	1.385	1.156	1.322	1.216
F(000)	1572	1572	2358	1956
Radiation type	Cu Kα	Cu Kα	Cu Kα	Cu Kα
Absorption coefficient / mm^{-1}	4.521	3.773	4.316	3.337
Crystal size / mm^3	0.18×0.12×0.04	0.20×0.10×0.08	0.14×0.10×0.08	0.16×0.12×0.06
2θ range / °	9.58–133.36	9.30–133.27	6.43–133.37	6.55–133.36
Completeness to max 2θ	0.996	0.978	1.000	0.997
No. of reflections measured	26460	18058	68020	92463
No. of independent reflections	3223	3801	3403	8976
R(int.)	0.0399	0.0340	0.0687	0.0600
No. parameters / restraints	227 / 0	227 / 0	251 / 48	562 / 0
Final R1 values (I > 2s(I))	0.0300	0.0296	0.0347	0.0379
Final wR(F^2) values (all data)	0.0856	0.0801	0.0900	0.1044
Goodness-of-fit on F^2	1.040	1.071	1.060	1.040
Largest diff. peak & hole / e $Å^{-3}$	0.260, –0.333	0.235, –0.249	0.361, –0.395	0.936, –0.367

[a] The SQUEEZE algorithm[4] has been applied to handle poorly-resolved dichloromethane molecules (two per 2,6-X_3TTM molecule).

Table S8. Dihedrals between the least-square planes of the phenyl rings and the corresponding phenyl-phenyl bond lengths

Radical	Mol. index	Point symmetry	φ^1 (°)[a]	φ^2 (°)[a]	φ^3 (°)[a]	Ar[1] (Å)[a]	Ar[2] (Å)[a]	Ar[3] (Å)[a]
P_3TTM	1	1	37.5	31.4	26.3	1.488	1.473	1.466
	2	1	53.4	32.4	31.6	1.489	1.487	1.493
	3	1	41.0	34.1	33.8	1.474	1.478	1.485
	4	1	40.1	34.1	21.4	1.483	1.484	1.486
4-T_3TTM	1	2 (C_2)	30.9	22.6	22.6	1.487	1.482	1.482
	2	1	32.5	27.4	10.0	1.458	1.475	1.484
	3	1	34.0	22.5	11.8	1.480	1.483	1.488
3-T_3TTM	1	2 (C_2)	51.3 [b]	30.9	30.9	1.485 [b]	1.486	1.486
	2	1	24.6/37.5 [b]	30.3	25.7	1.526/1.375 [b]	1.487	1.487
2-T_3TTM	1	1	49.5/47.9 [b]	75.9	68.6	1.541/1.515 [b]	1.493	1.495
3,5-X_3TTM	1	2 (C_2)	30.6	29.4	29.4	1.485	1.489	1.489
2,6-X_3TTM	1	2 (C_2)	84.1	72.8	72.8	1.499	1.497	1.497
2,4-X_3TTM	1	3 (C_3)	64.4	64.4	64.4	1.490	1.490	1.490
	2	32 (D_3)	37.6 [b]	37.6 [b]	37.6 [b]	1.522 [b]	1.522 [b]	1.522 [b]
M_3TTM	1	2	87.8	81.7	81.7	1.498	1.505	1.505
2,6-ipP_3TTM	1	1	89.0	81.9	70.0	1.505	1.500	1.501

[a] The three dihedrals φ^1, φ^2 and φ^3 and the corresponding phenyl-phenyl bonds are illustrated in Figure 4a in the main manuscript. [b] The molecule shows disorder of aryl groups (see discussion above).

S7. EPR Spectroscopy

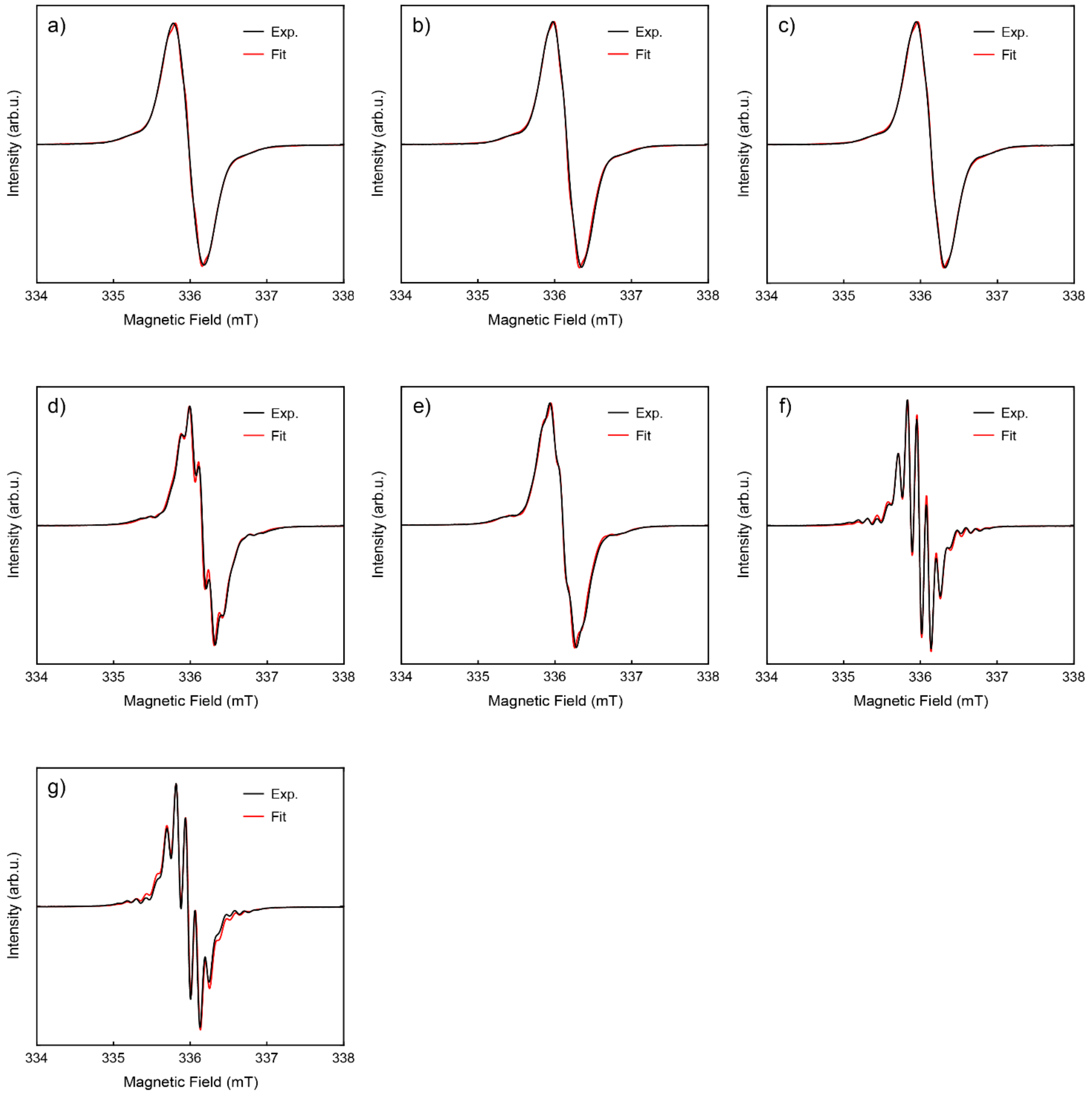

Figure S7. Continuous-wave X-band EPR spectra for a) 4-T_3TTM, b) 3-T_3TTM, c) 3,5-X_3TTM, d) 2,5-X_3TTM, e) 2,4-X_3TTM, f) M_3TTM and g) 2,6-ipP_3TTM measured at 200 K in 1 mM toluene solution (black) and simulated spectra (red) with fit parameters given in Table S9.

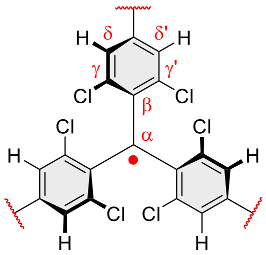

Table S9. Summary of the EPR data measured at 200 K and relevant fit parameters

Radical	g-value	Linewidth (mT)	^{13}C, α (MHz)[a,b]	^{13}C, β (MHz)[a,c]	^{13}C, γ/γ' (MHz)[a,d]	^{1}H, δ/δ' (MHz)[a,e]
P_3TTM	2.0156	0.248	34.5	3.0	--[f]	3.12
4-T_3TTM	2.0156	0.270	33.5	3.2	--[f]	3.35
3-T_3TTM	2.0156	0.260	33.0	3.0	--[f]	3.12
2-T_3TTM	2.0156	0.171	24.5	3.8	3.6	3.28
3,5-X_3TTM	2.0156	0.265	33.0	3.0	--[f]	3.20
2,6-X_3TTM	2.0156	0.149	26.0	3.6	3.4	3.40
2,5-X_3TTM	2.0156	0.205	33.5	3.2	--[f]	3.40
2,4-X_3TTM	2.0156	0.225	34.5	3.0	--[f]	3.12
M_3TTM	2.0156	0.136	26.0	3.6	3.4	3.37
2,6-ipP_3TTM	2.0156	0.164	25.7	3.6	3.5	3.45

[a] Hyperfine coupling constant between the unpaired electron and [b] α-carbon, [c] β-carbon, [d] γ/γ'-carbon and [e] δ/δ'-hydrogen nuclei. [f] Hyperfine coupling not resolved.

S8. NMR Spectra

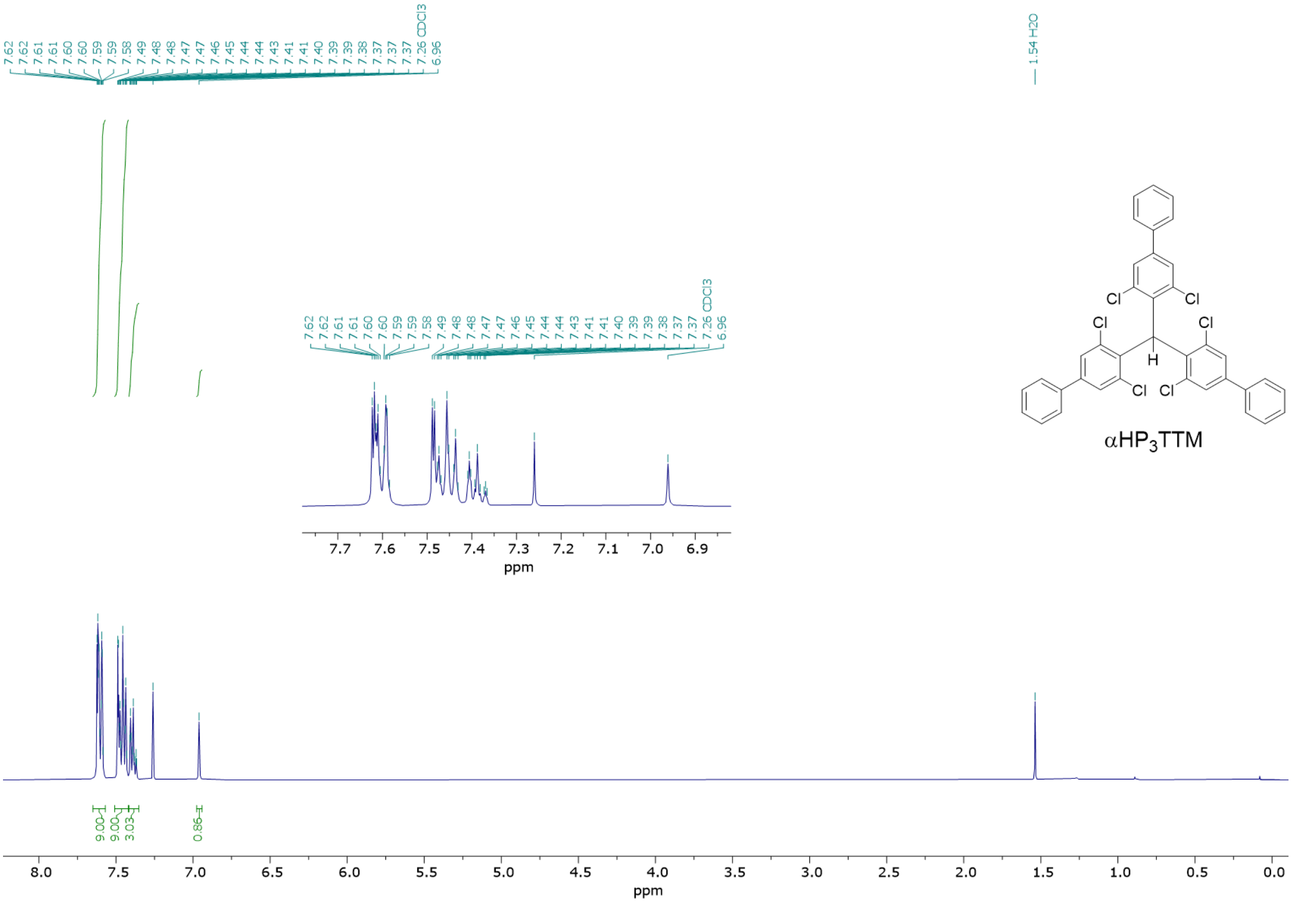

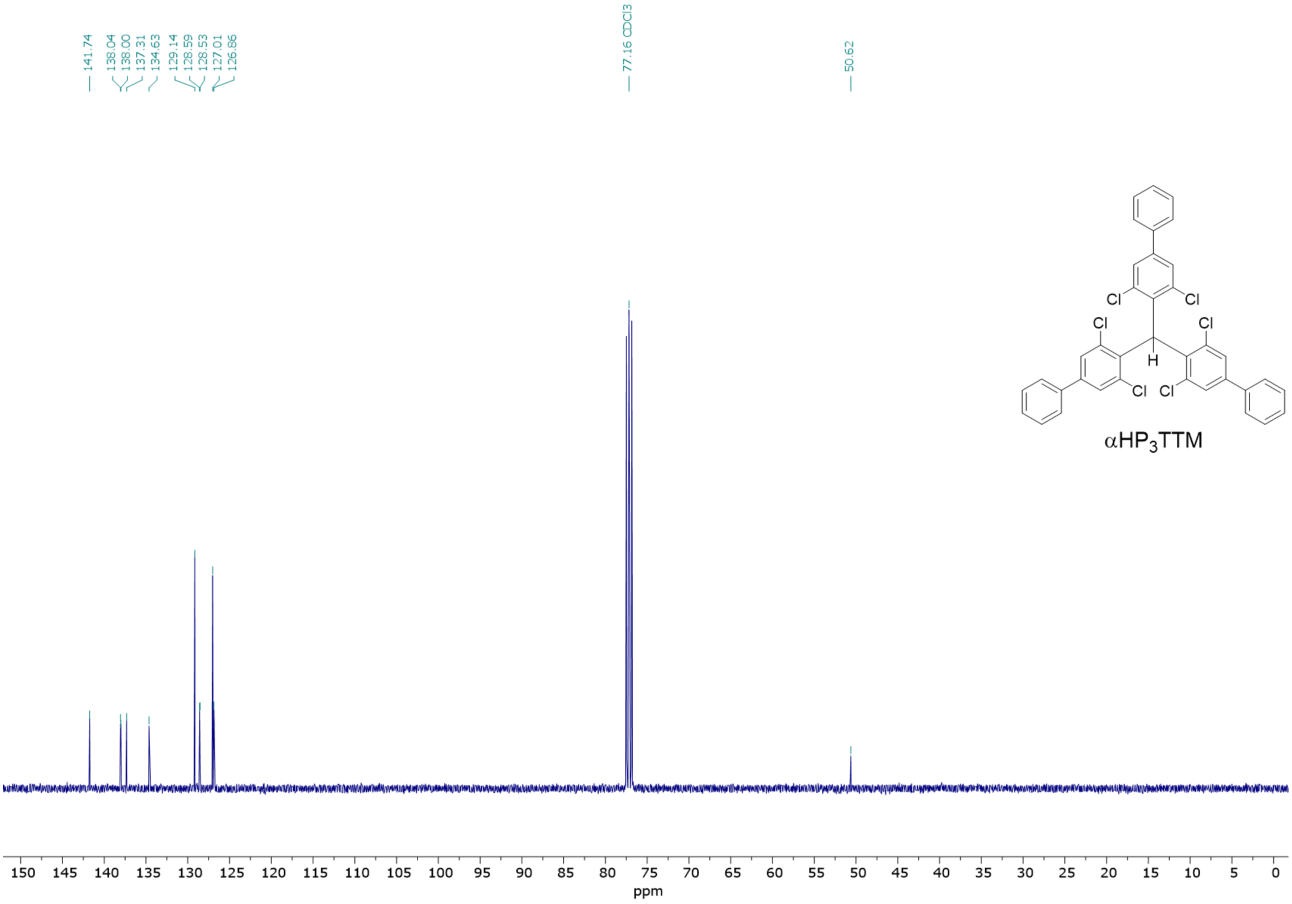

COSY

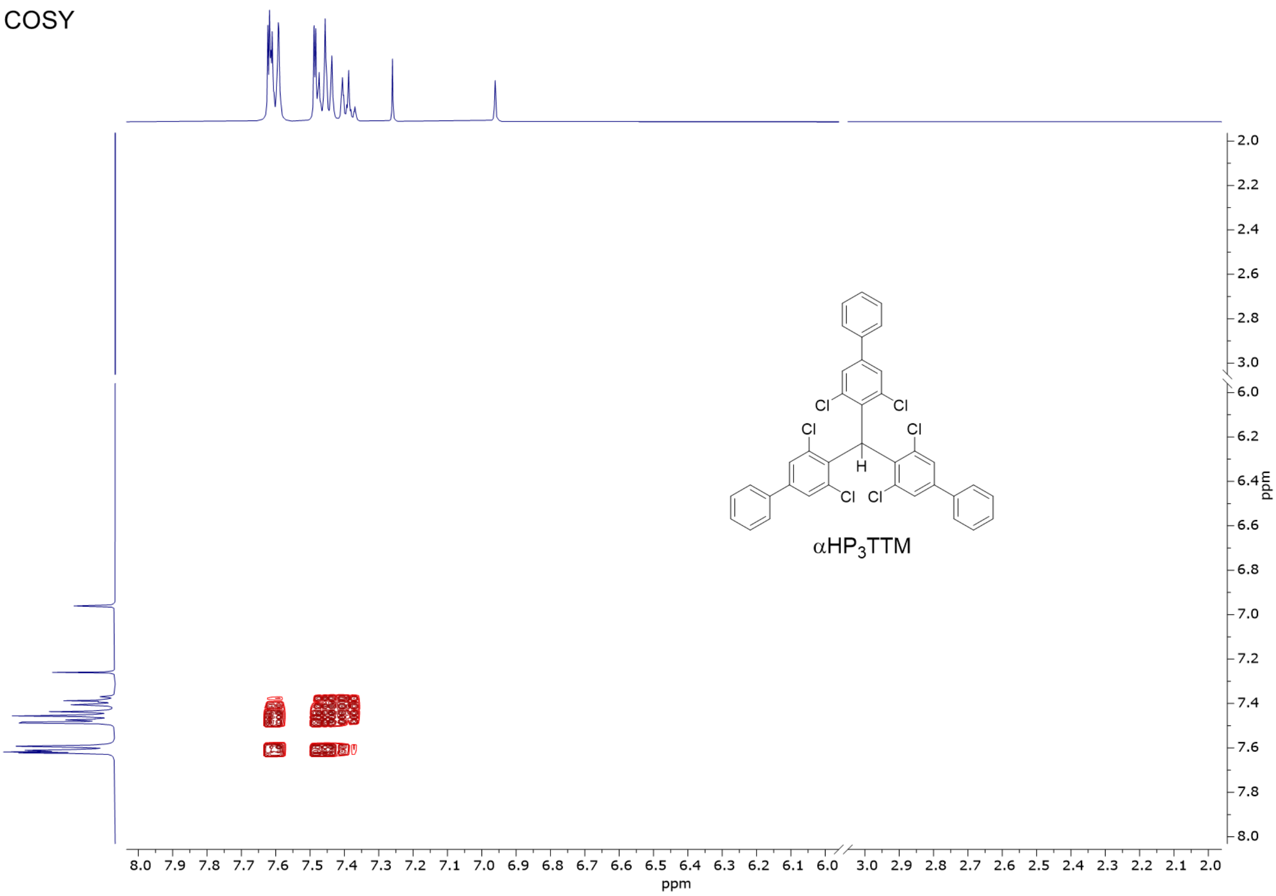

HSQC

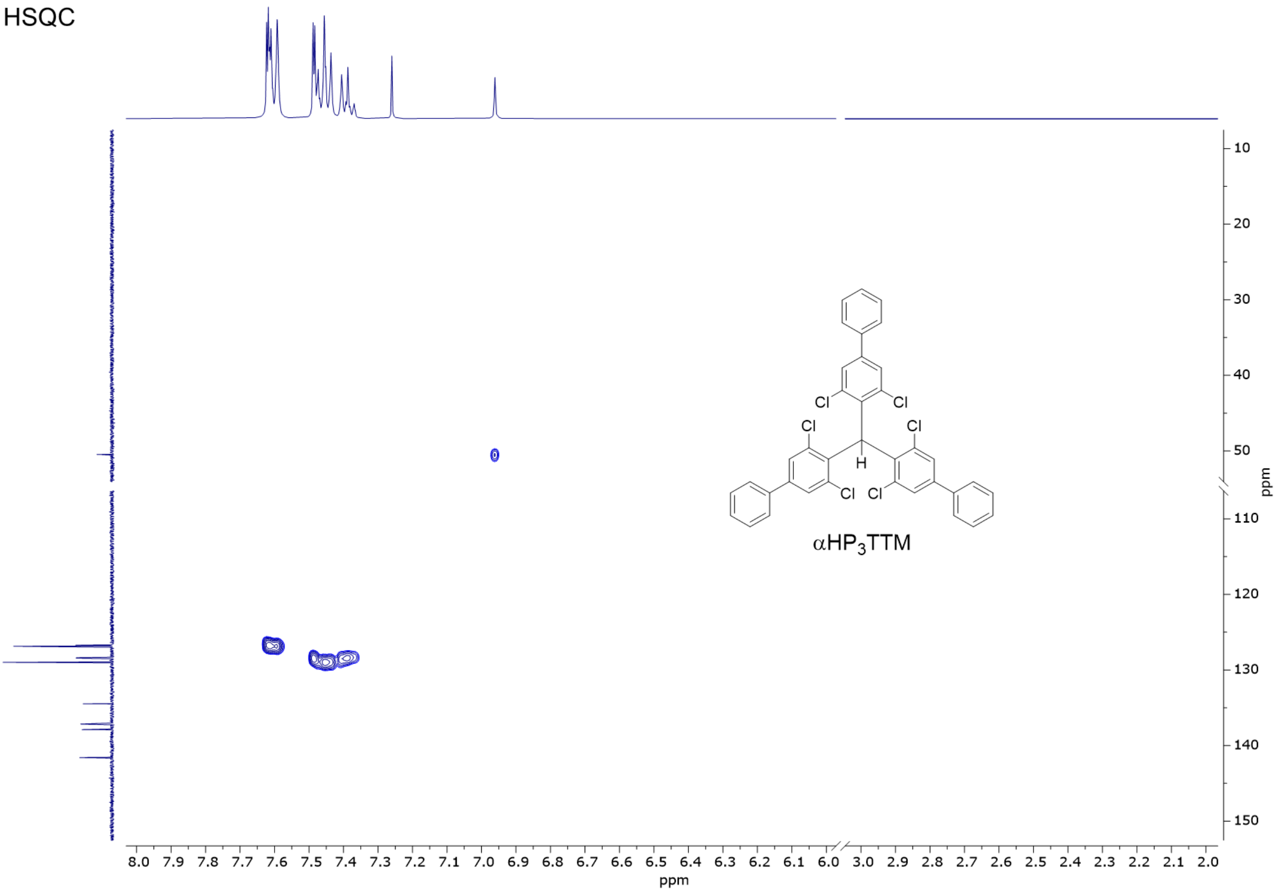

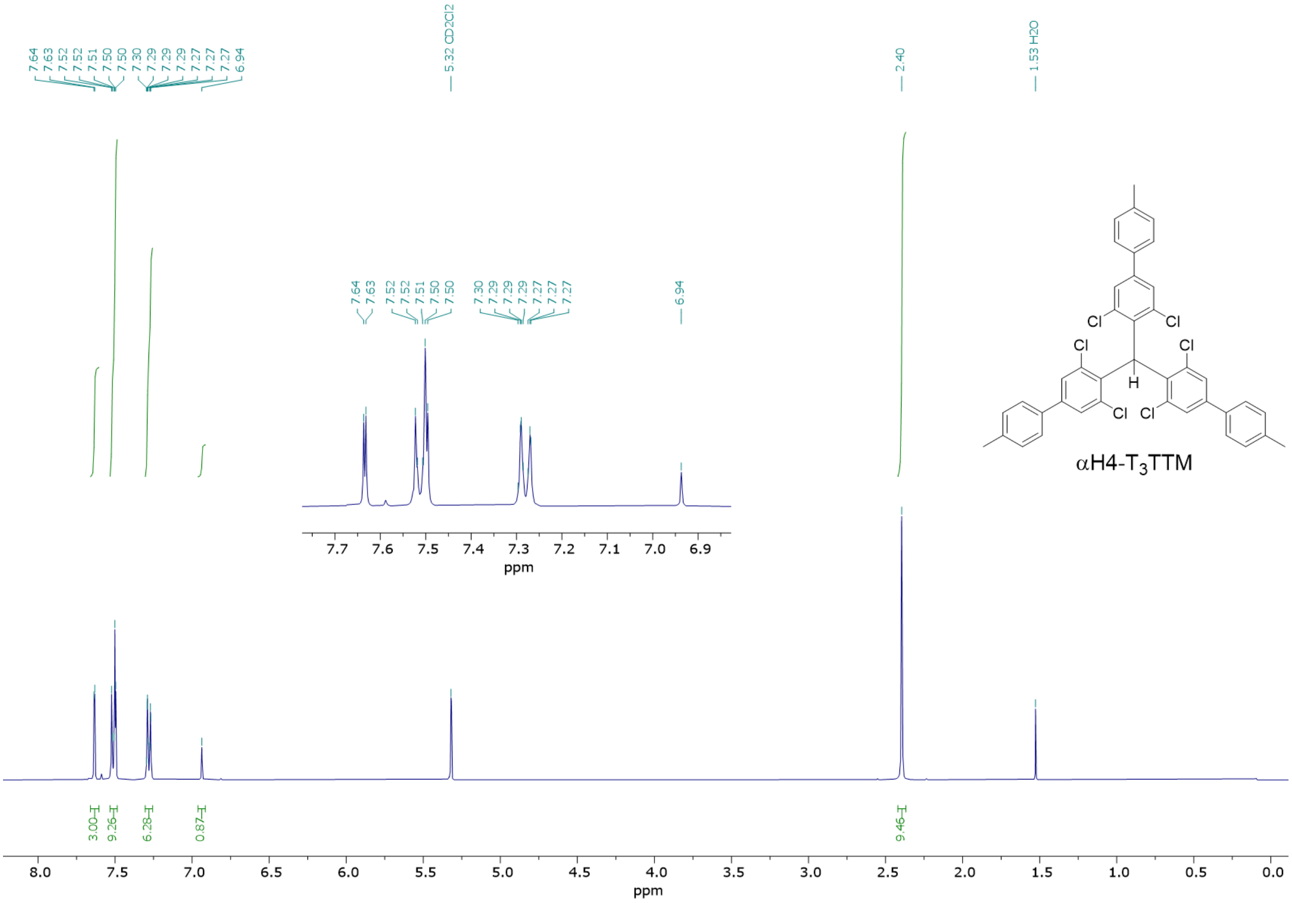
7.64
7.63
7.52
7.52
7.51
7.50
7.50
7.30
7.29
7.29
7.29
7.27
7.27
7.27
6.94
5.32 CD2Cl2
2.40
1.53 H2O
3.00
9.26
6.28
0.87
9.46
ppm
αH4-T_3TTM

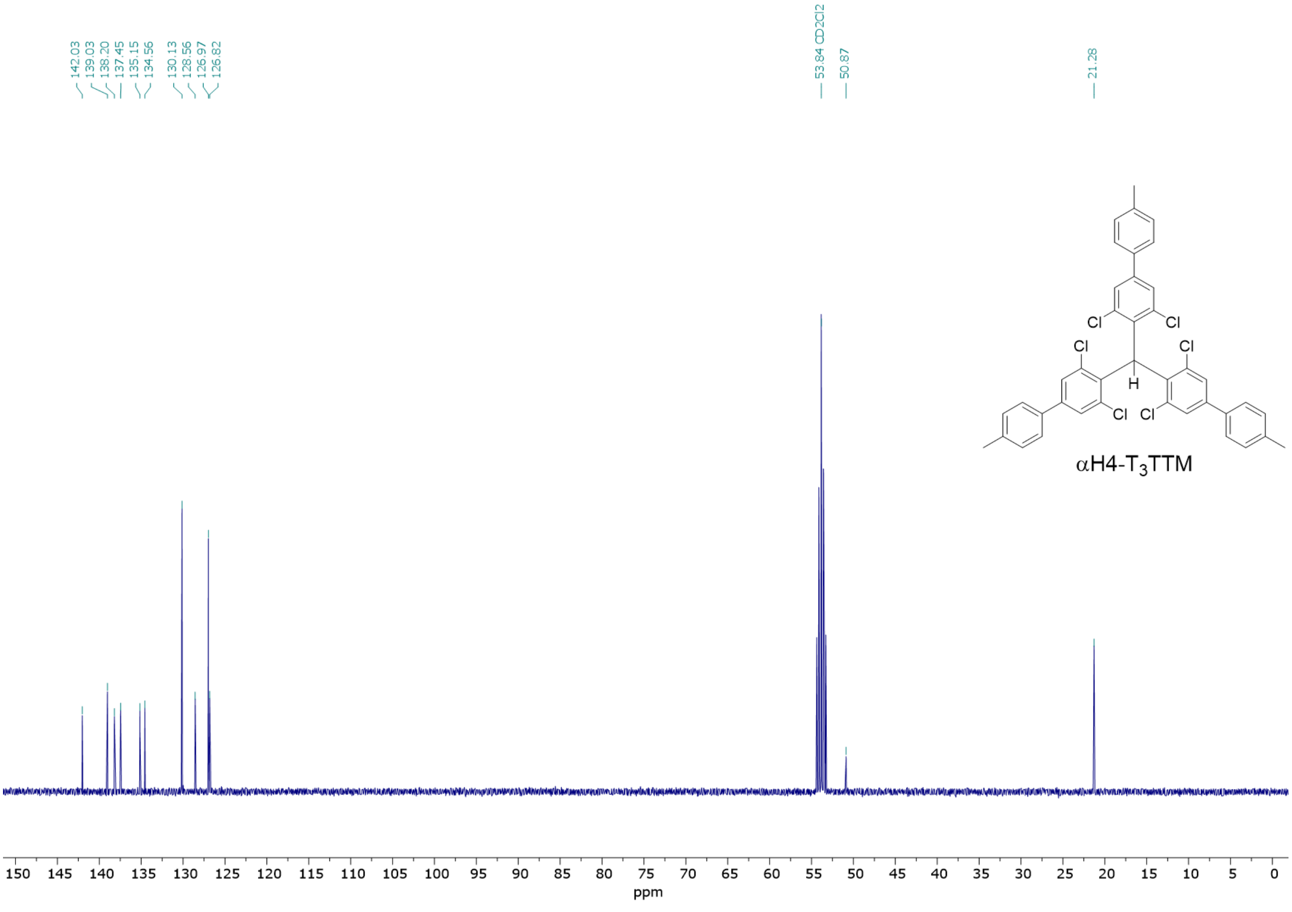
142.03
139.03
138.20
137.45
135.15
134.56
130.13
128.56
126.97
126.82
53.84 CD2Cl2
50.87
21.28
ppm
αH4-T_3TTM

COSY

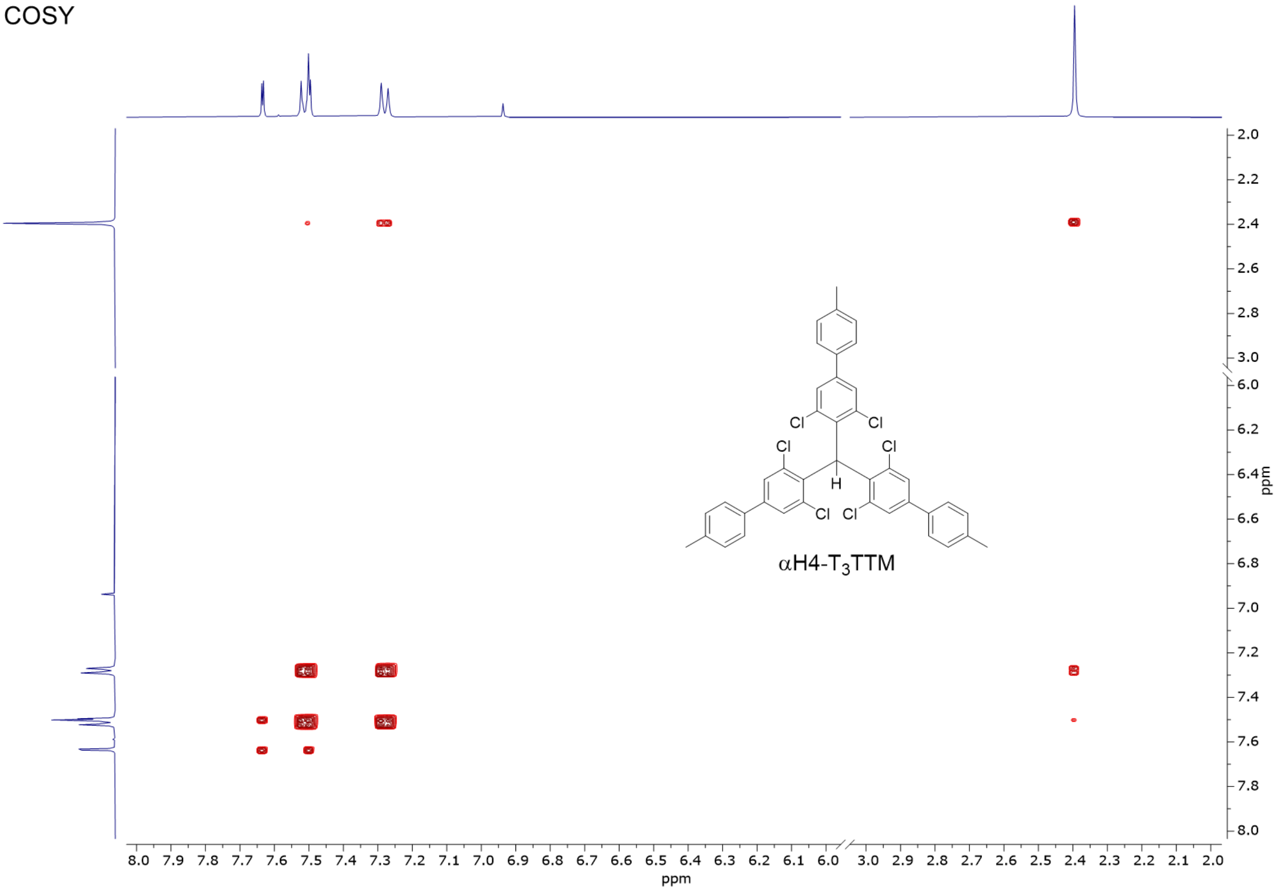

HSQC

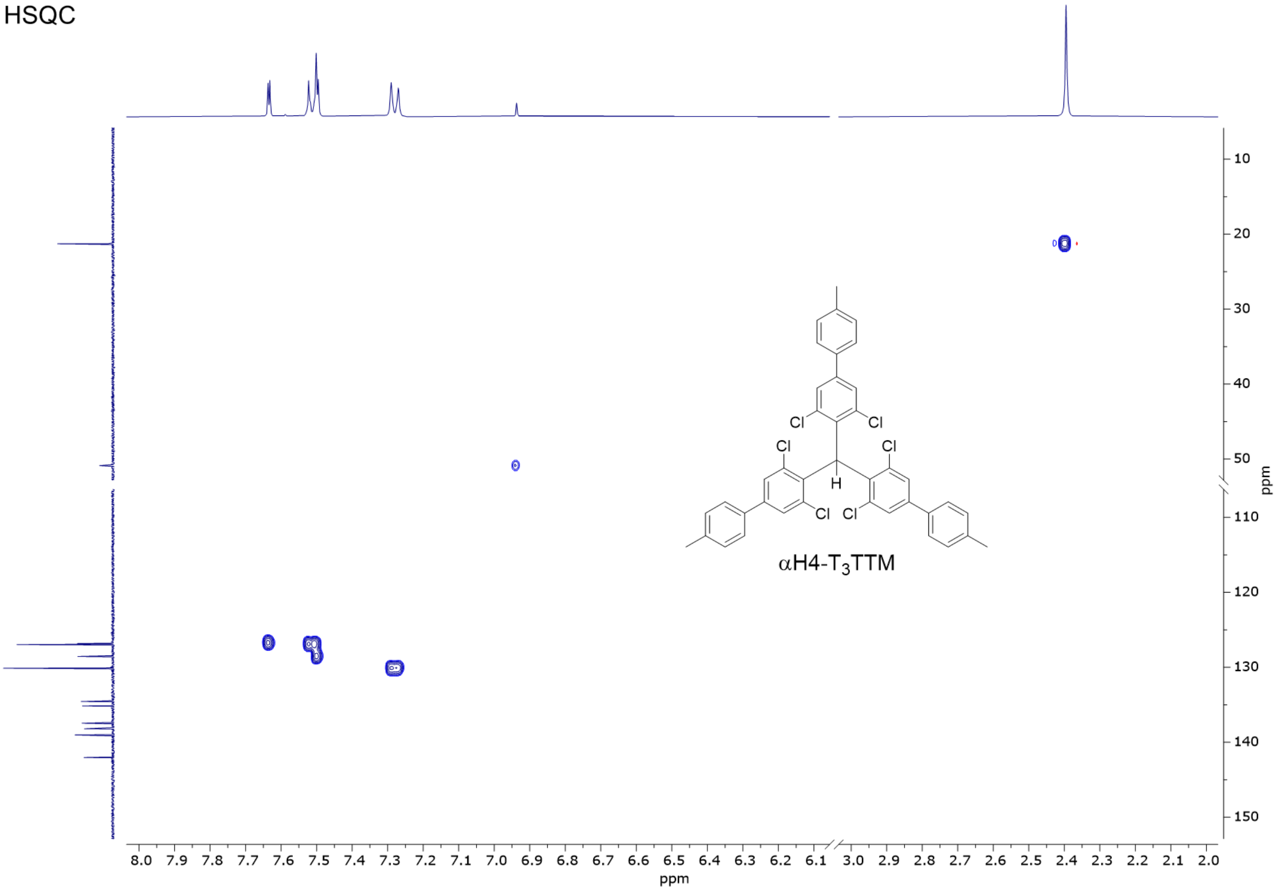

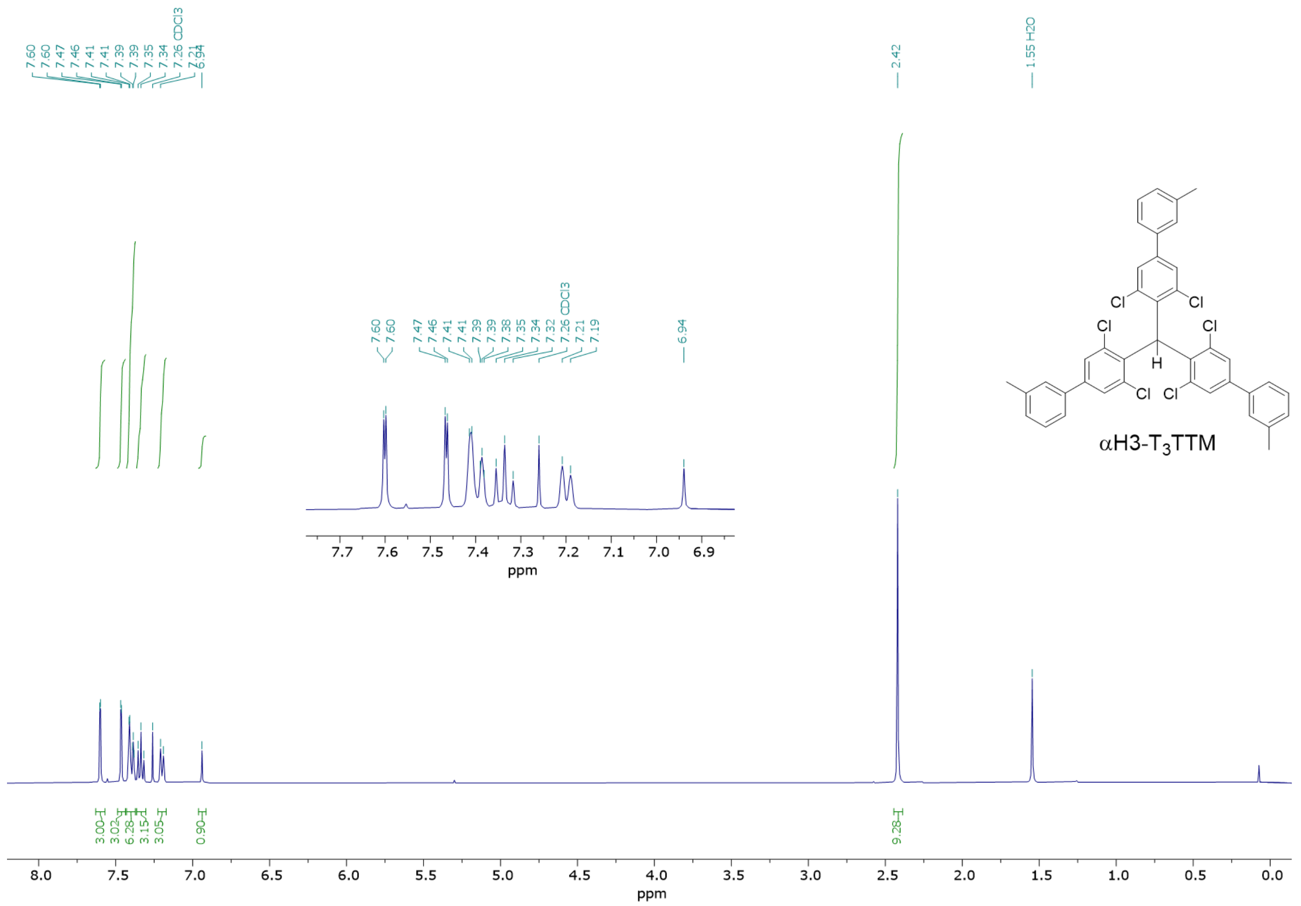

αH3-T3TTM
ppm

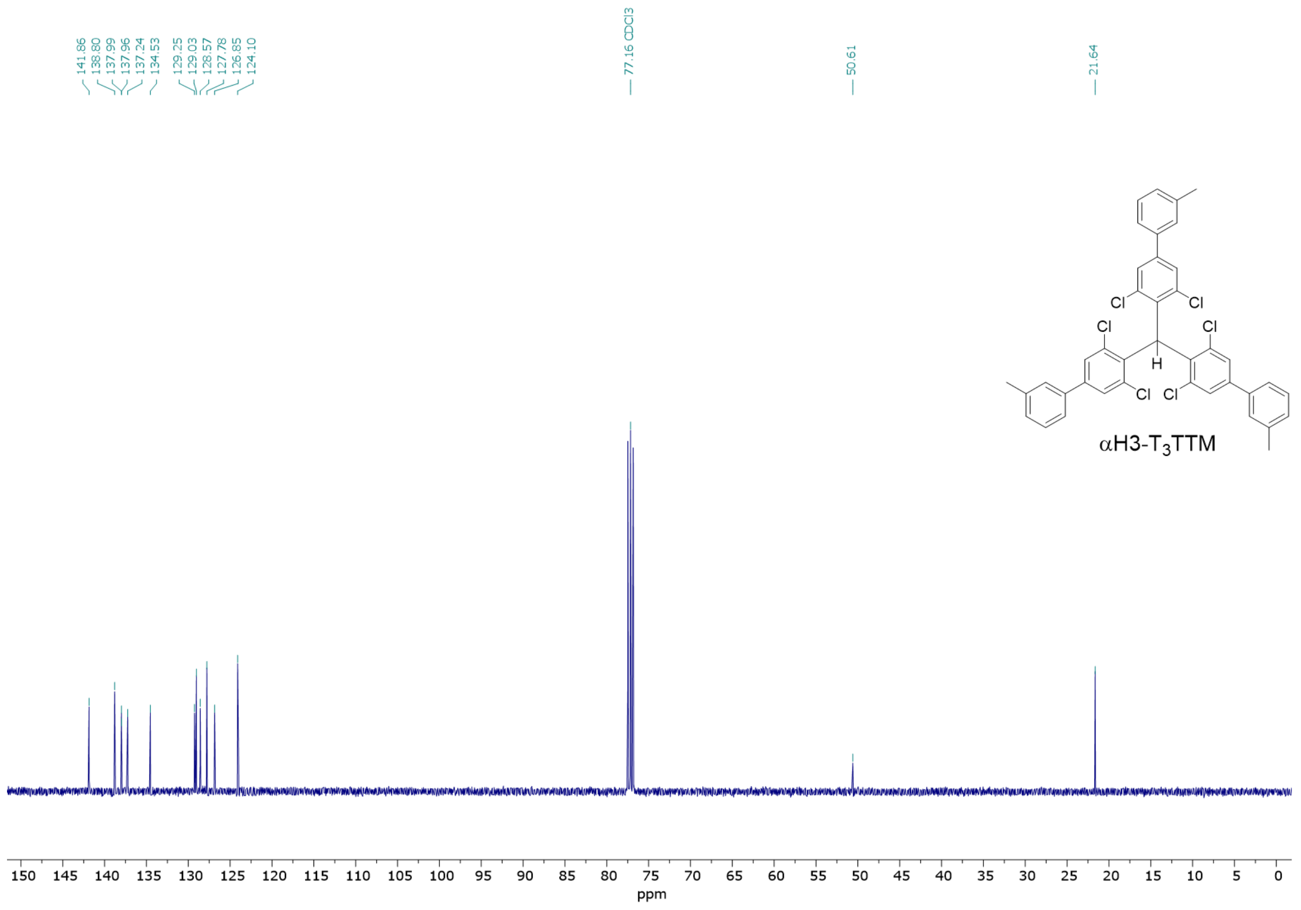

αH3-T3TTM
ppm

COSY

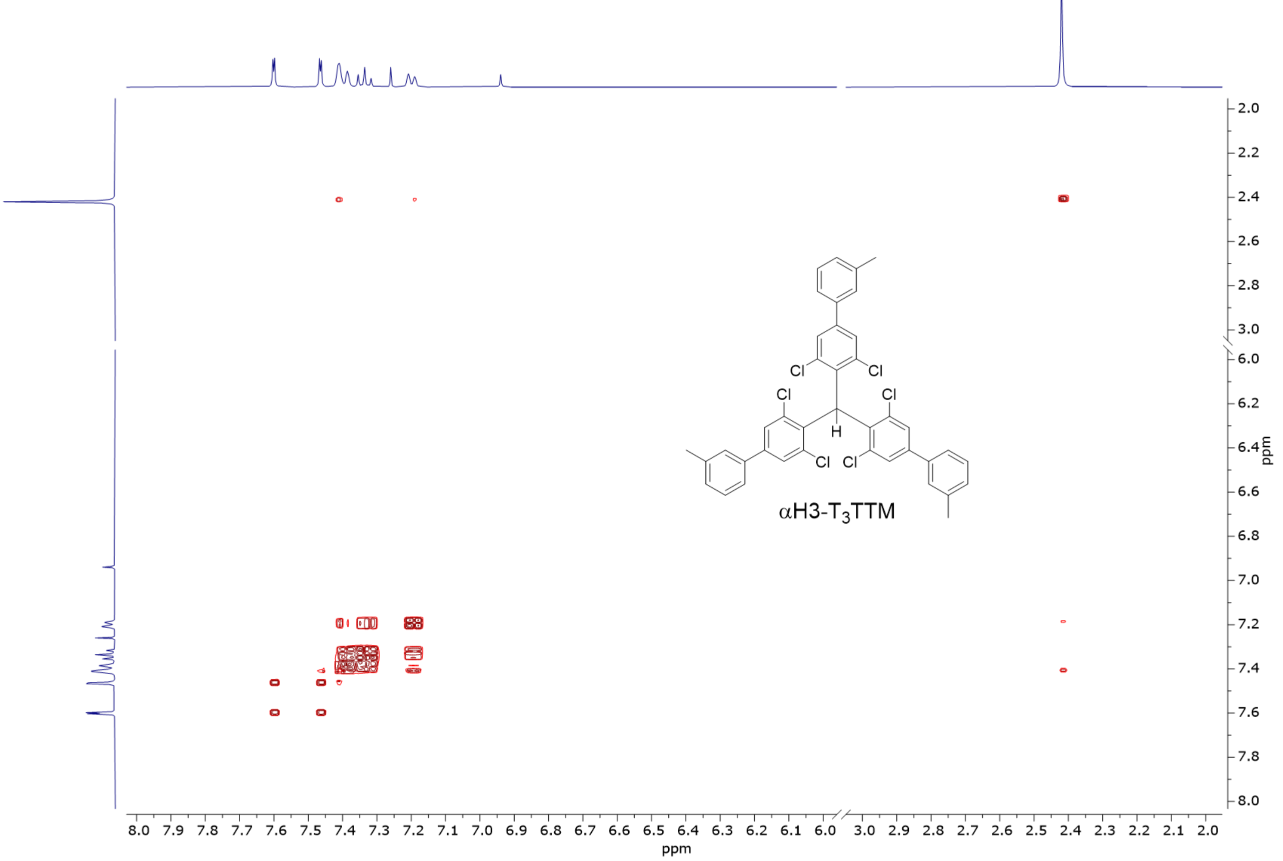

HSQC

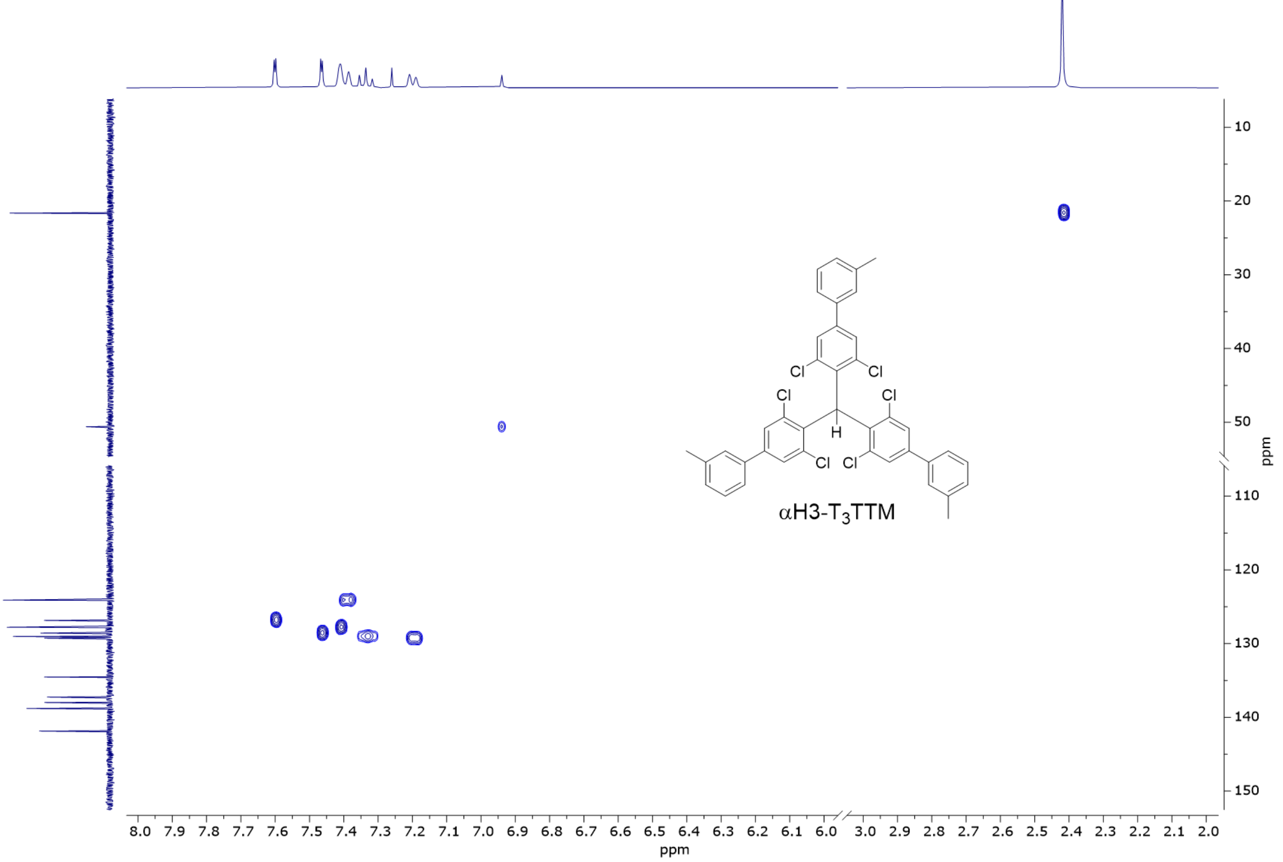

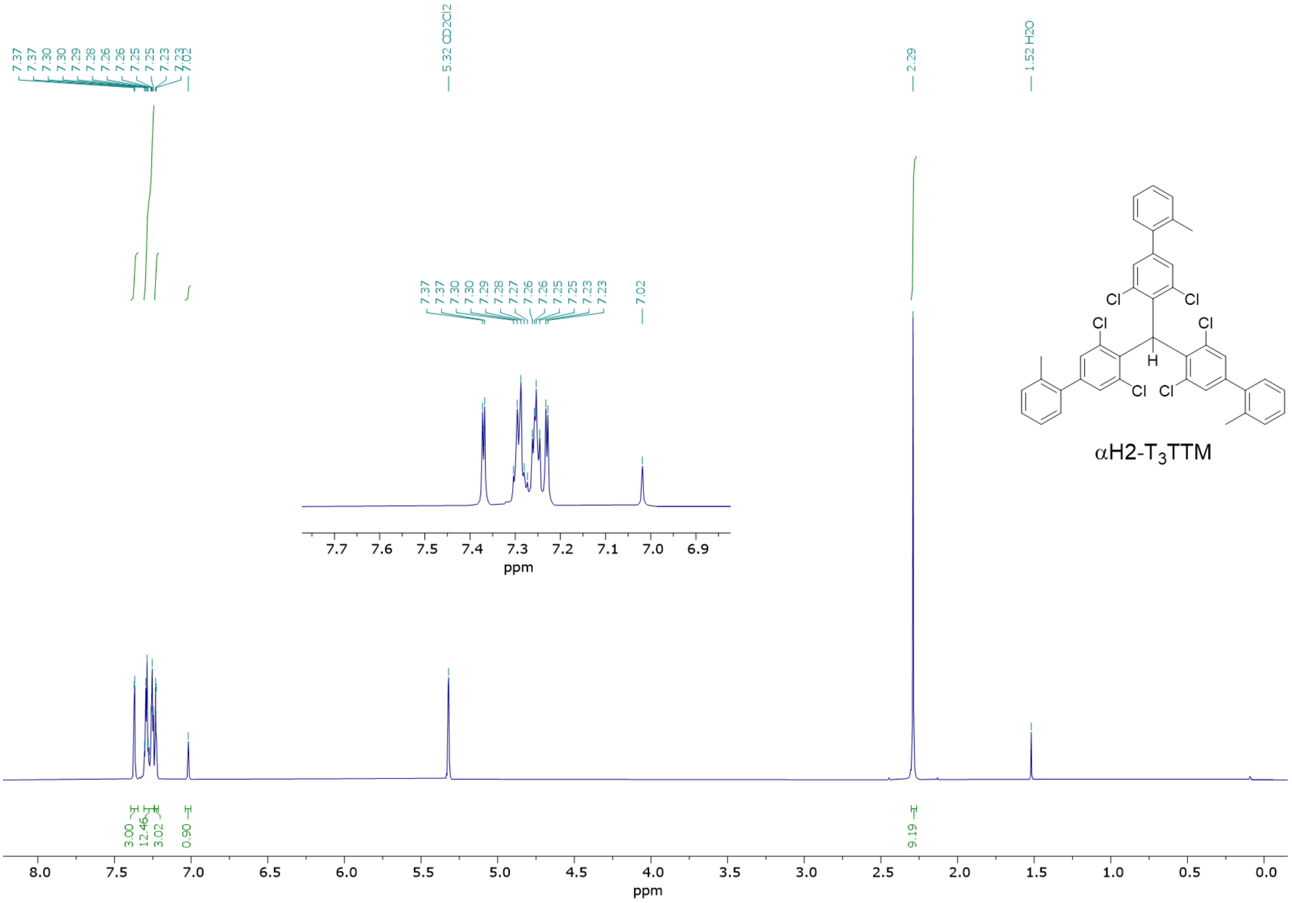
7.37
7.37
7.30
7.30
7.29
7.28
7.26
7.26
7.25
7.25
7.23
7.23
7.02
5.32 CD2Cl2
2.29
1.52 H2O
3.00
12.46
3.02
0.90
9.19
ppm
αH2-T3TTM

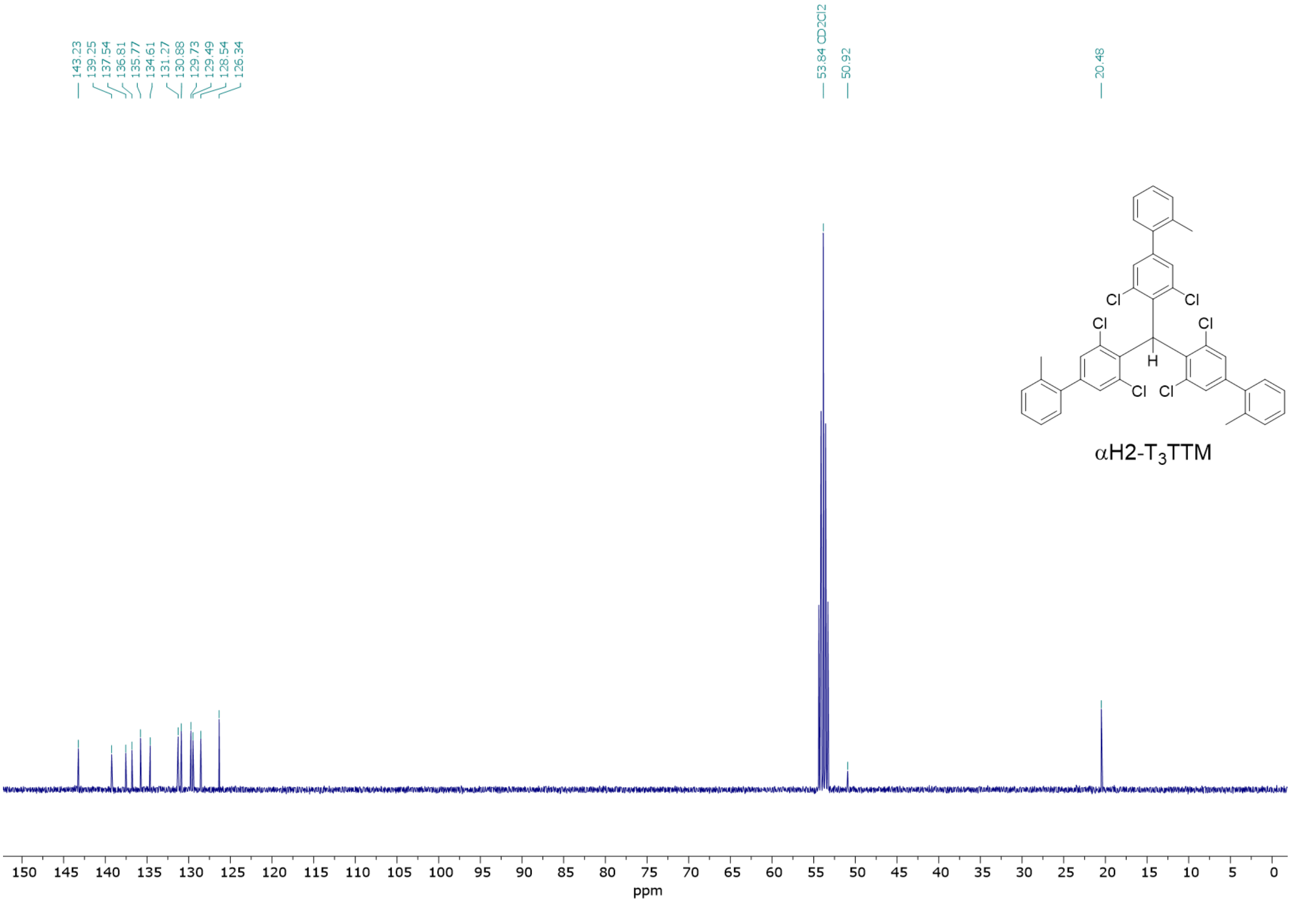
143.23
139.25
137.54
136.81
135.77
134.61
131.27
130.88
129.73
129.49
128.54
126.34
53.84 CD2Cl2
50.92
20.48
ppm
αH2-T3TTM

COSY

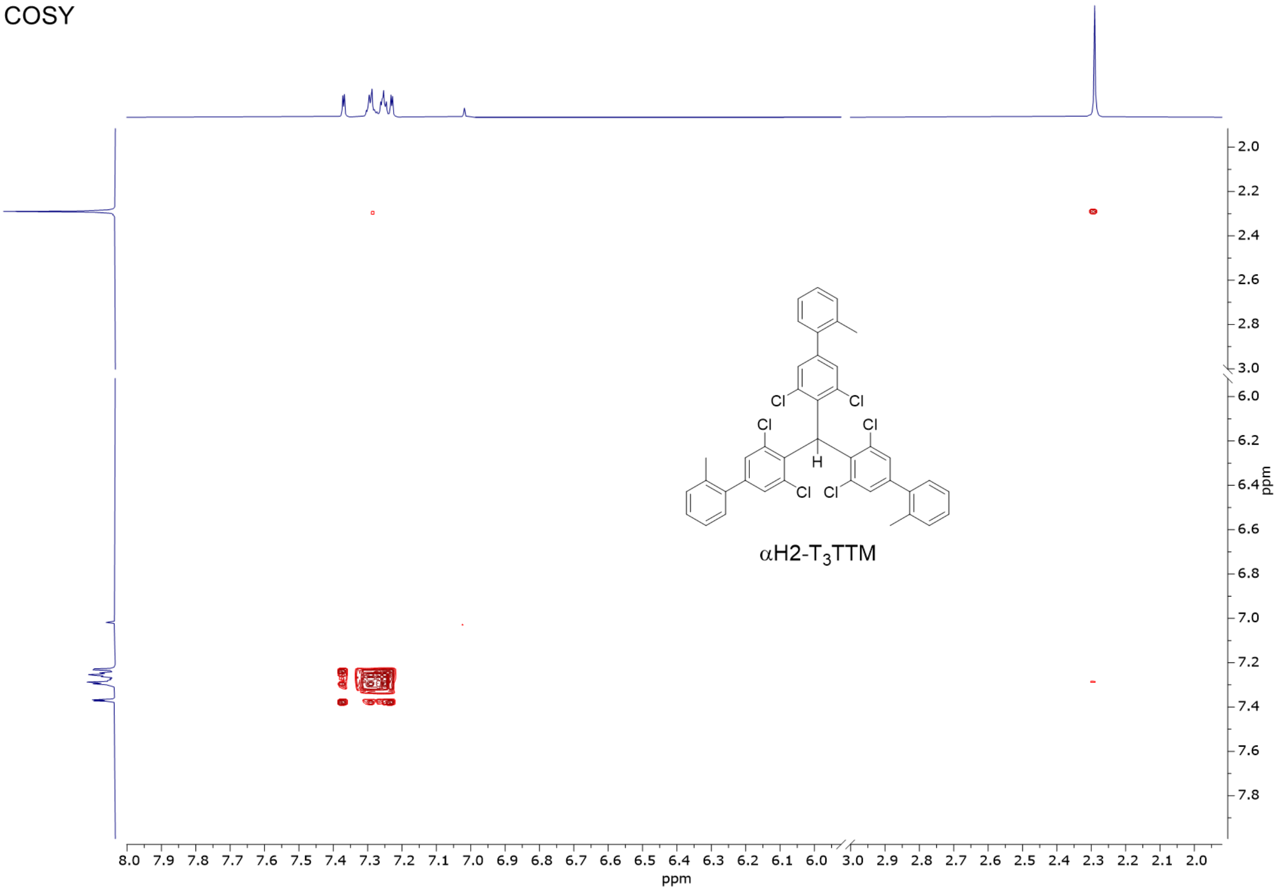
αH2-T3TTM
Cl
H
ppm
8.0 7.9 7.8 7.7 7.6 7.5 7.4 7.3 7.2 7.1 7.0 6.9 6.8 6.7 6.6 6.5 6.4 6.3 6.2 6.1 6.0 3.0 2.9 2.8 2.7 2.6 2.5 2.4 2.3 2.2 2.1 2.0
2.0 2.2 2.4 2.6 2.8 3.0 6.0 6.2 6.4 6.6 6.8 7.0 7.2 7.4 7.6 7.8

HSQC

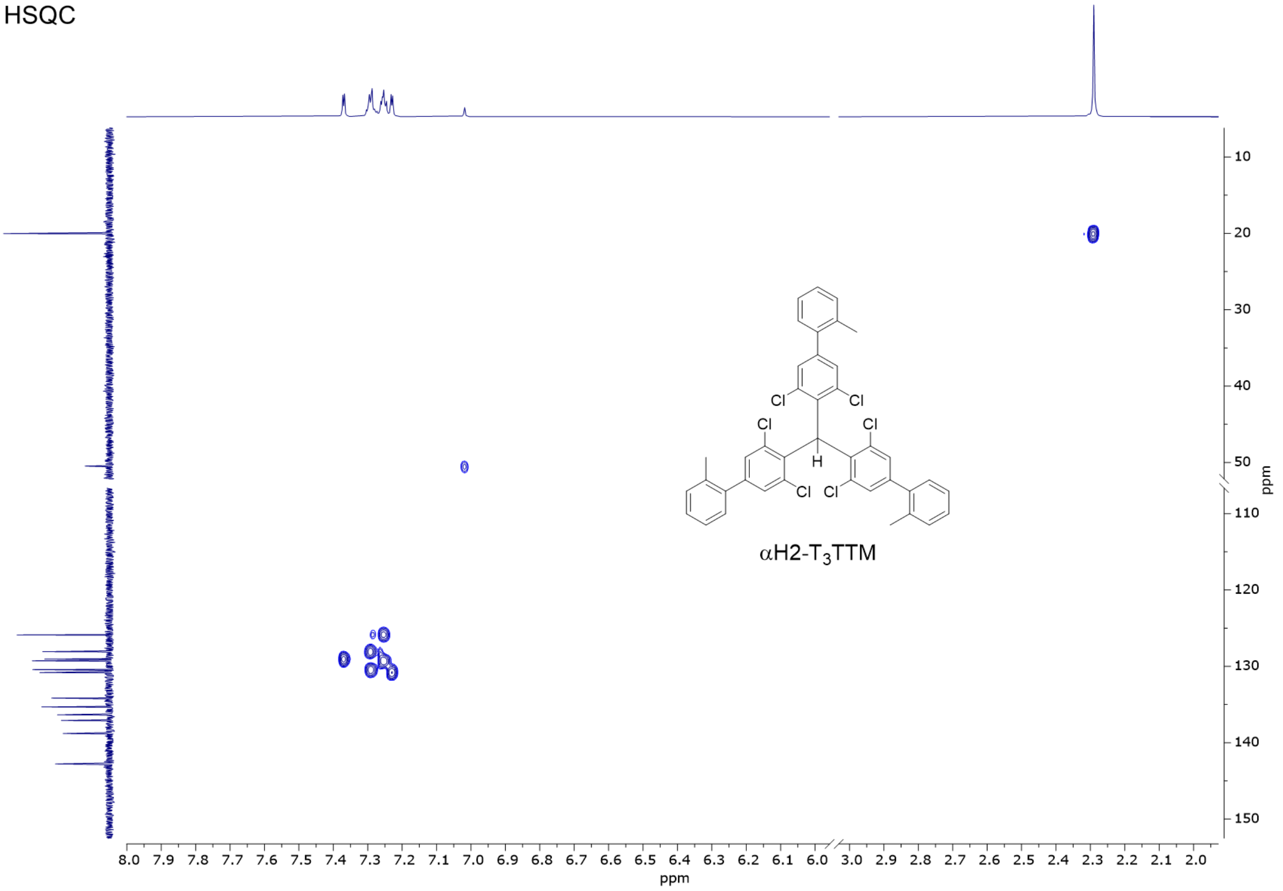
αH2-T3TTM
Cl
H
ppm
8.0 7.9 7.8 7.7 7.6 7.5 7.4 7.3 7.2 7.1 7.0 6.9 6.8 6.7 6.6 6.5 6.4 6.3 6.2 6.1 6.0 3.0 2.9 2.8 2.7 2.6 2.5 2.4 2.3 2.2 2.1 2.0
10 20 30 40 50 110 120 130 140 150

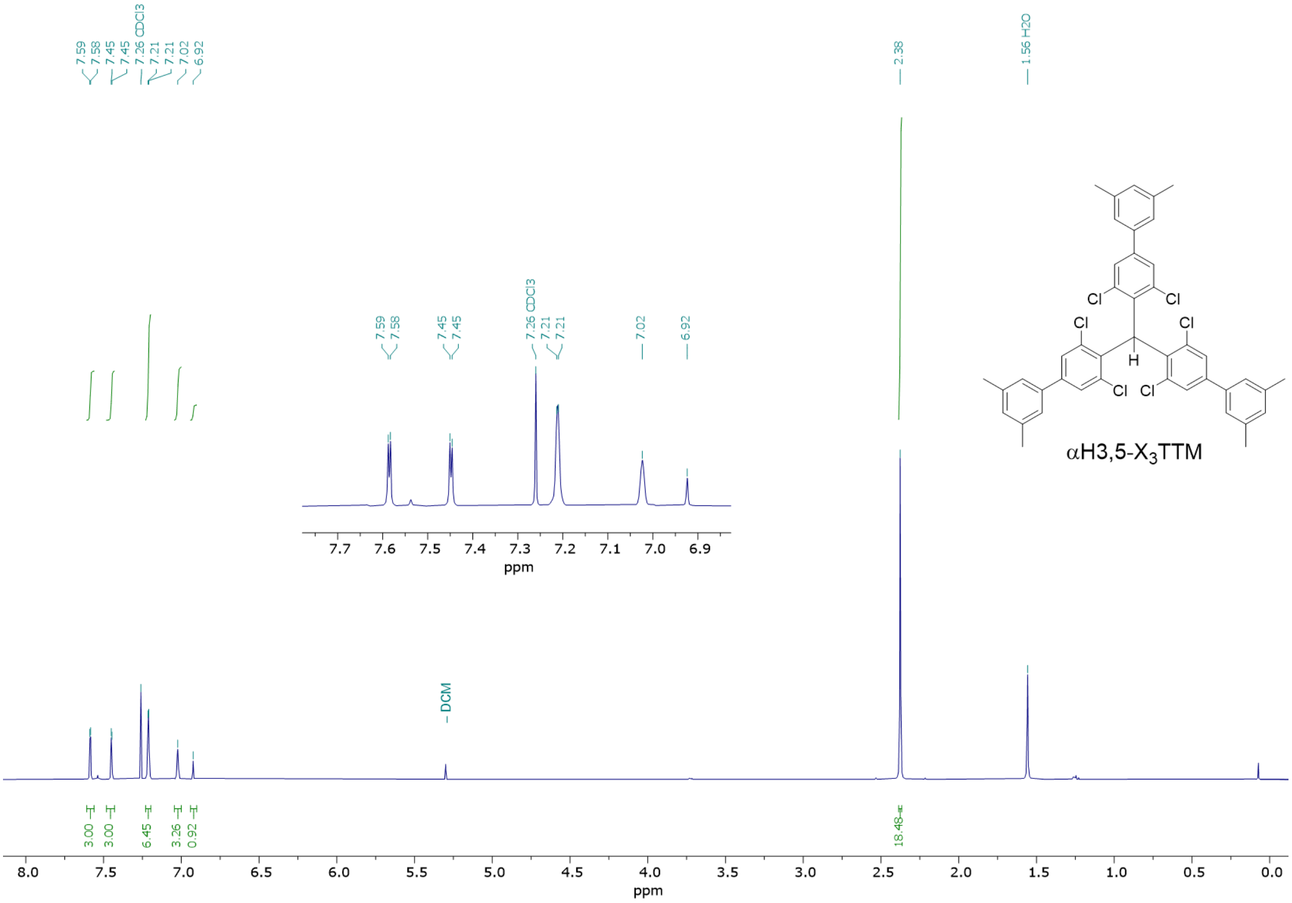
7.59
7.58
7.45
7.45
7.26 CDCl3
7.21
7.21
7.02
6.92
2.38
1.56 H2O
DCM
3.00
3.00
6.45
3.26
0.92
18.48
ppm
Cl
H
αH3,5-X3TTM

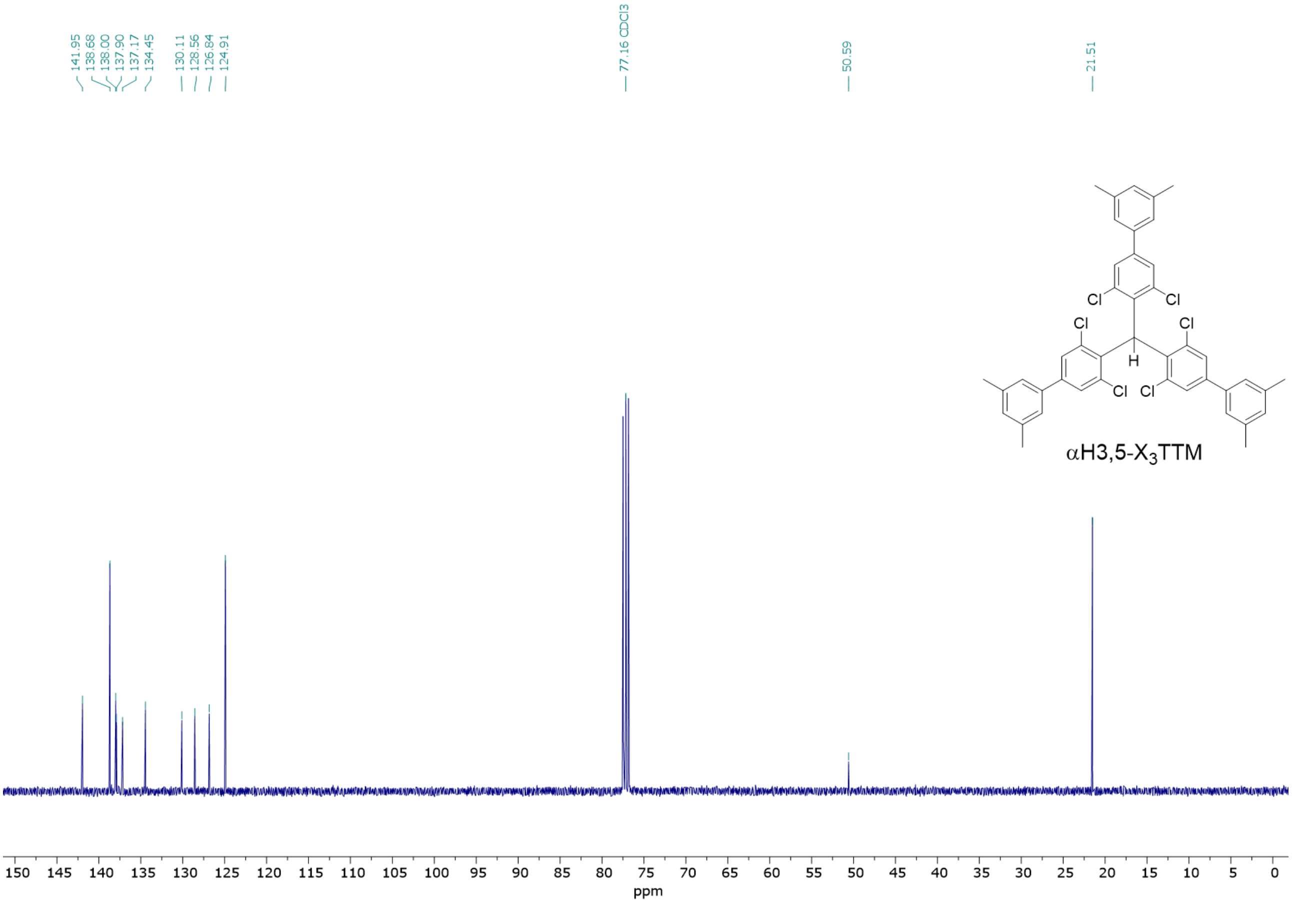
141.95
138.68
138.00
137.90
137.17
134.45
130.11
128.56
126.84
124.91
77.16 CDCl3
50.59
21.51
ppm
Cl
H
αH3,5-X3TTM

COSY

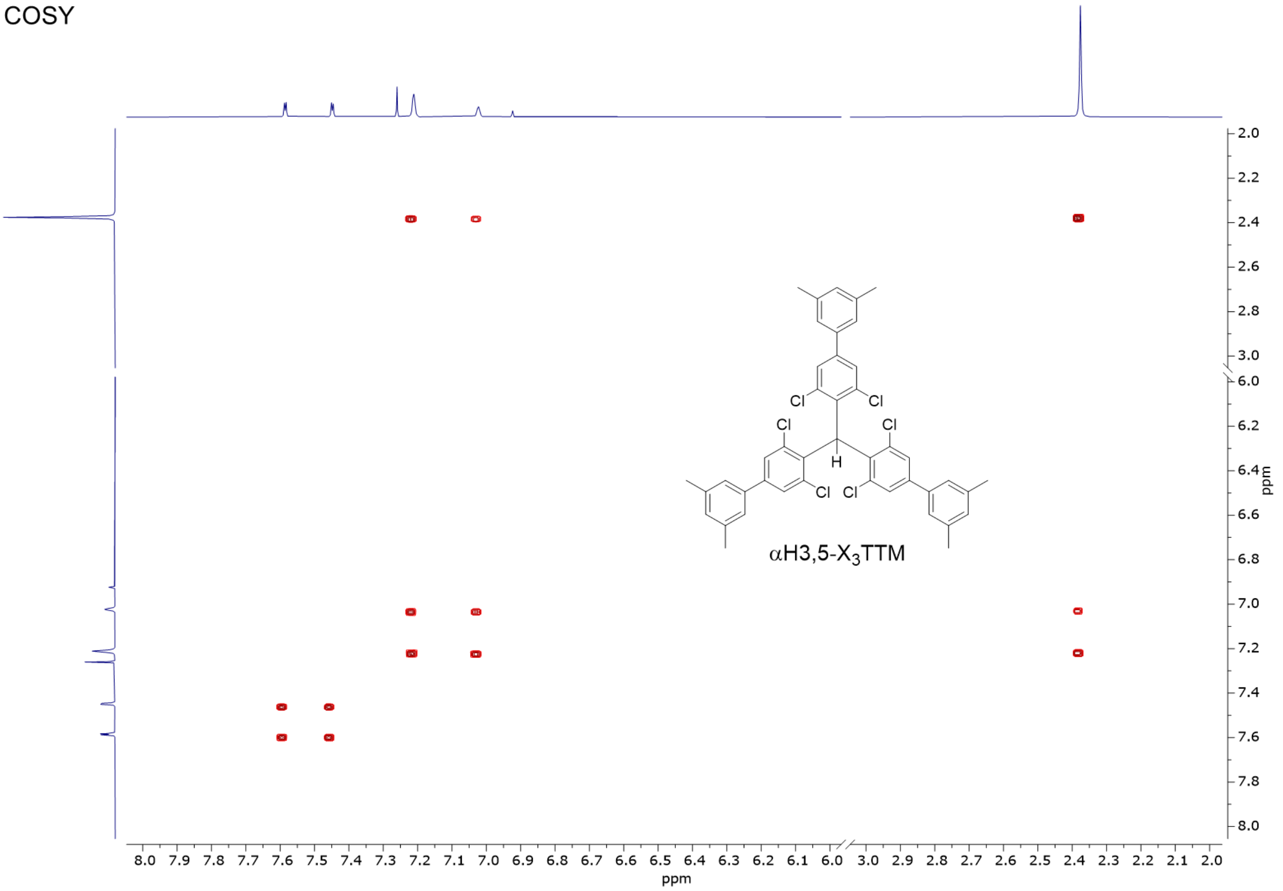

HSQC

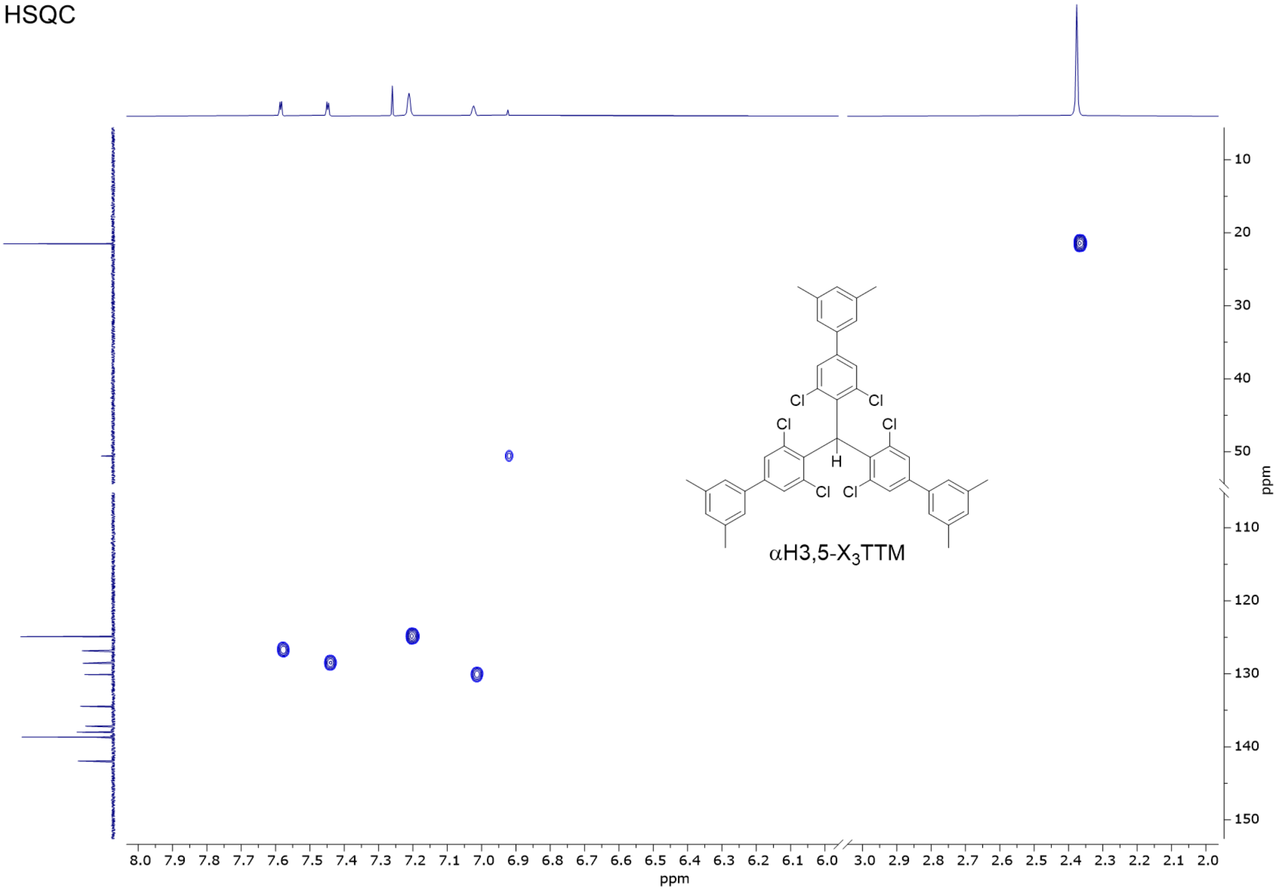

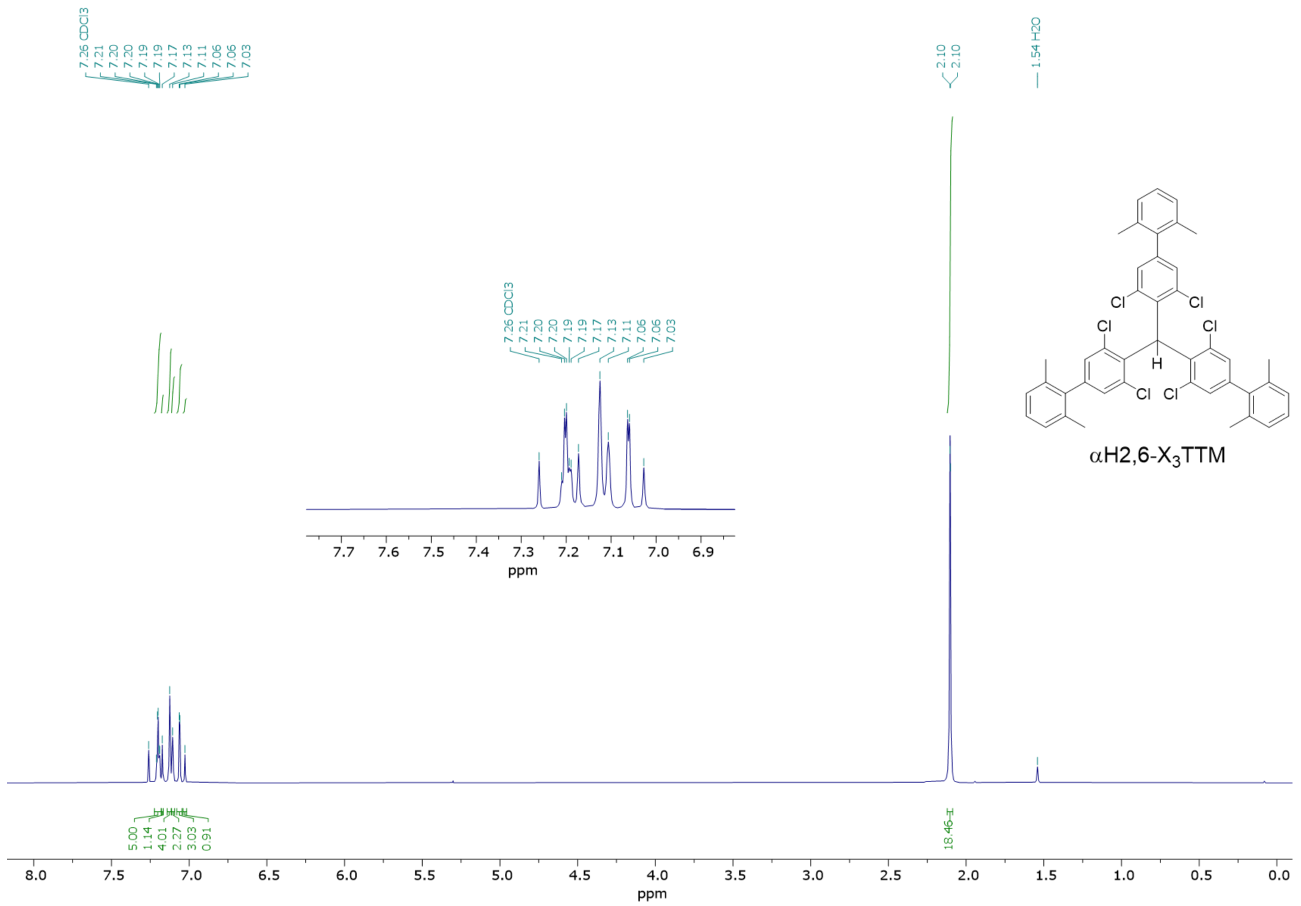
αH2,6-X3TTM
ppm

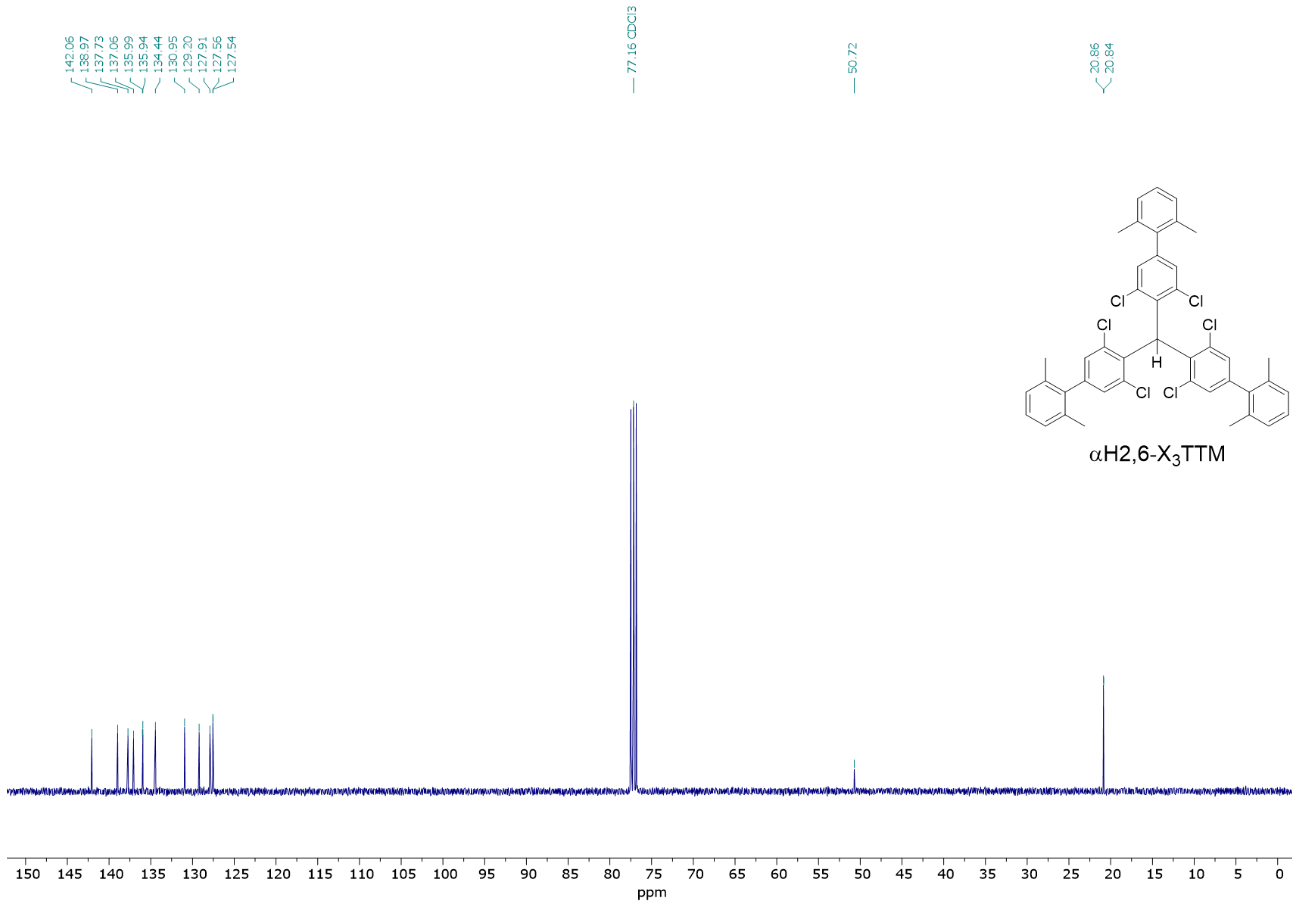
αH2,6-X3TTM
ppm

COSY

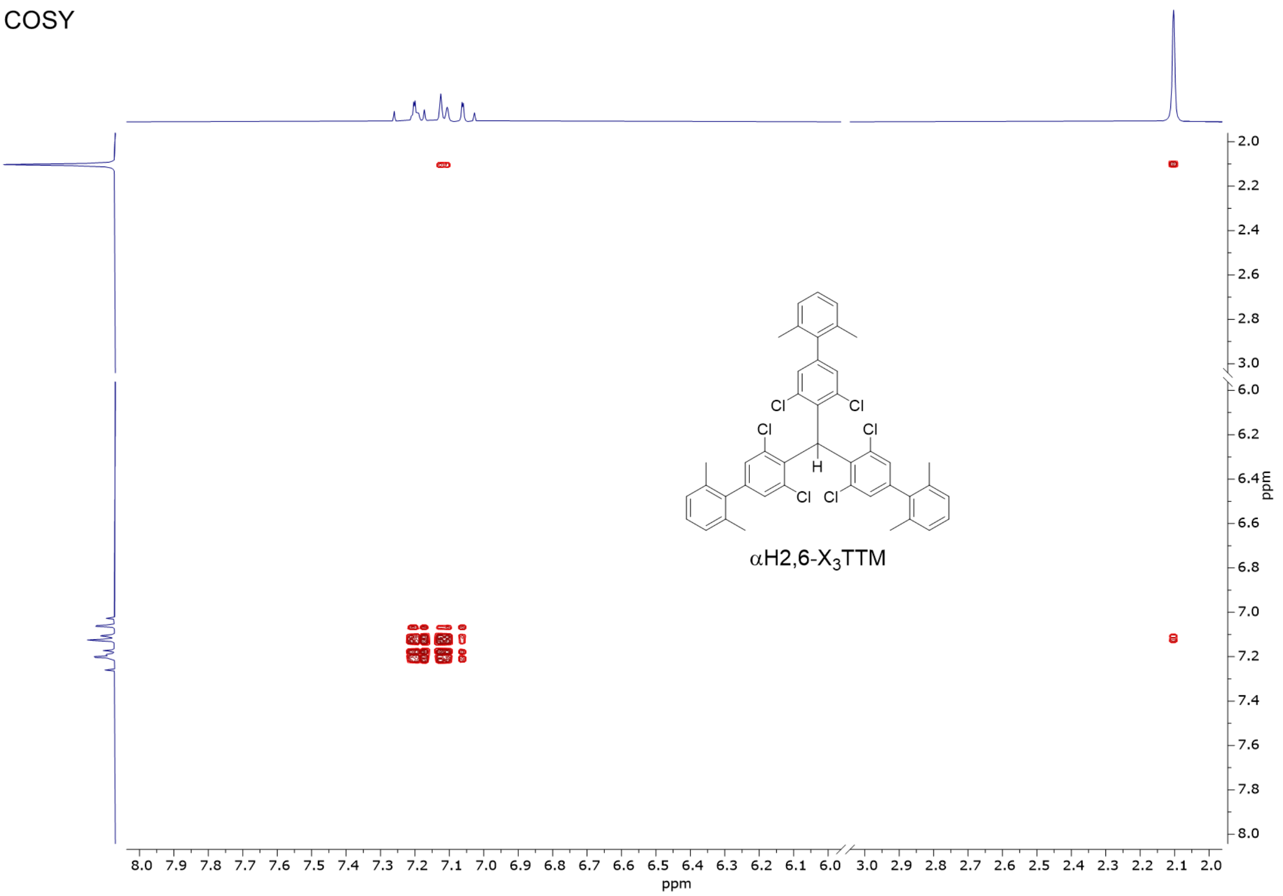

Cl
Cl
Cl
Cl
H
Cl
Cl
αH2,6-X3TTM
ppm
ppm

HSQC

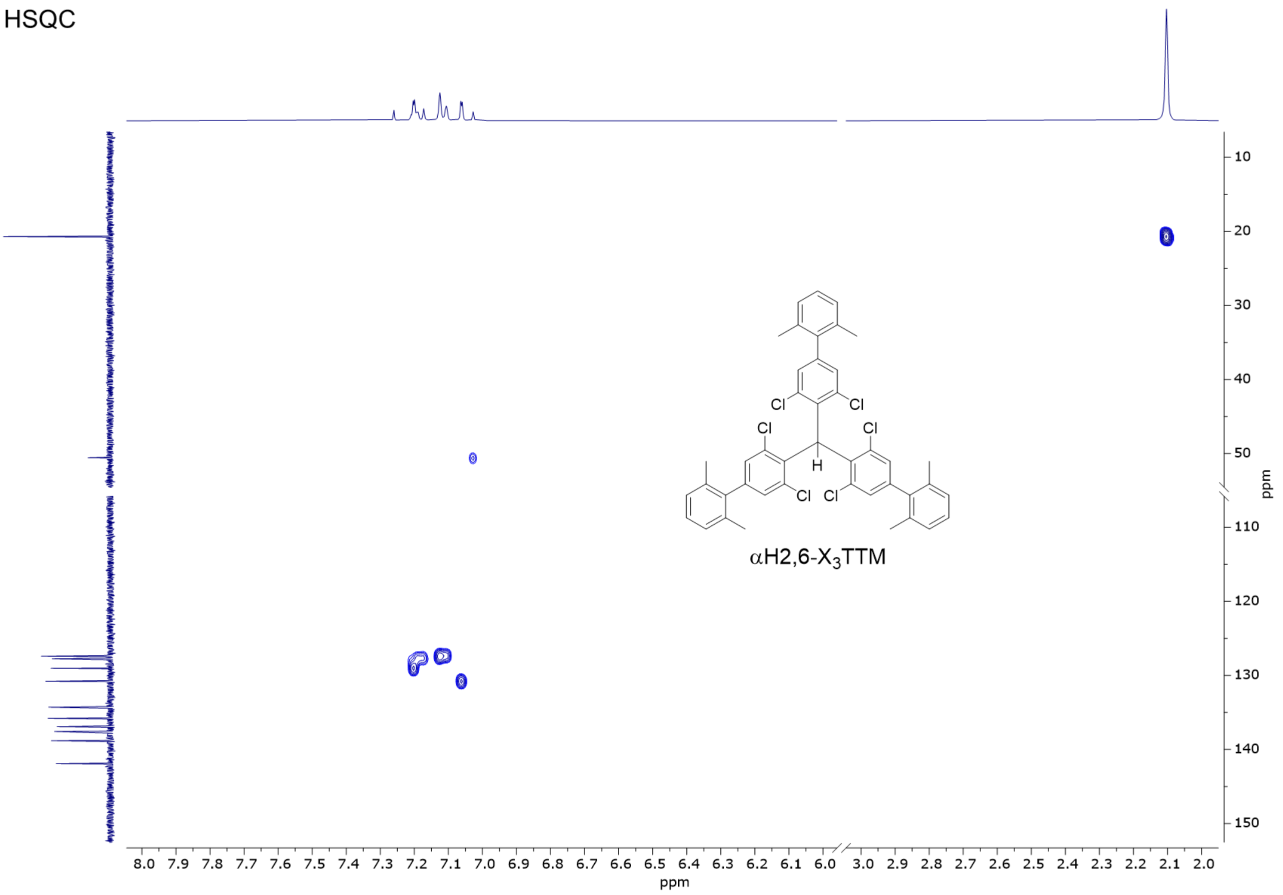

Cl
Cl
Cl
Cl
H
Cl
Cl
αH2,6-X3TTM
ppm
ppm

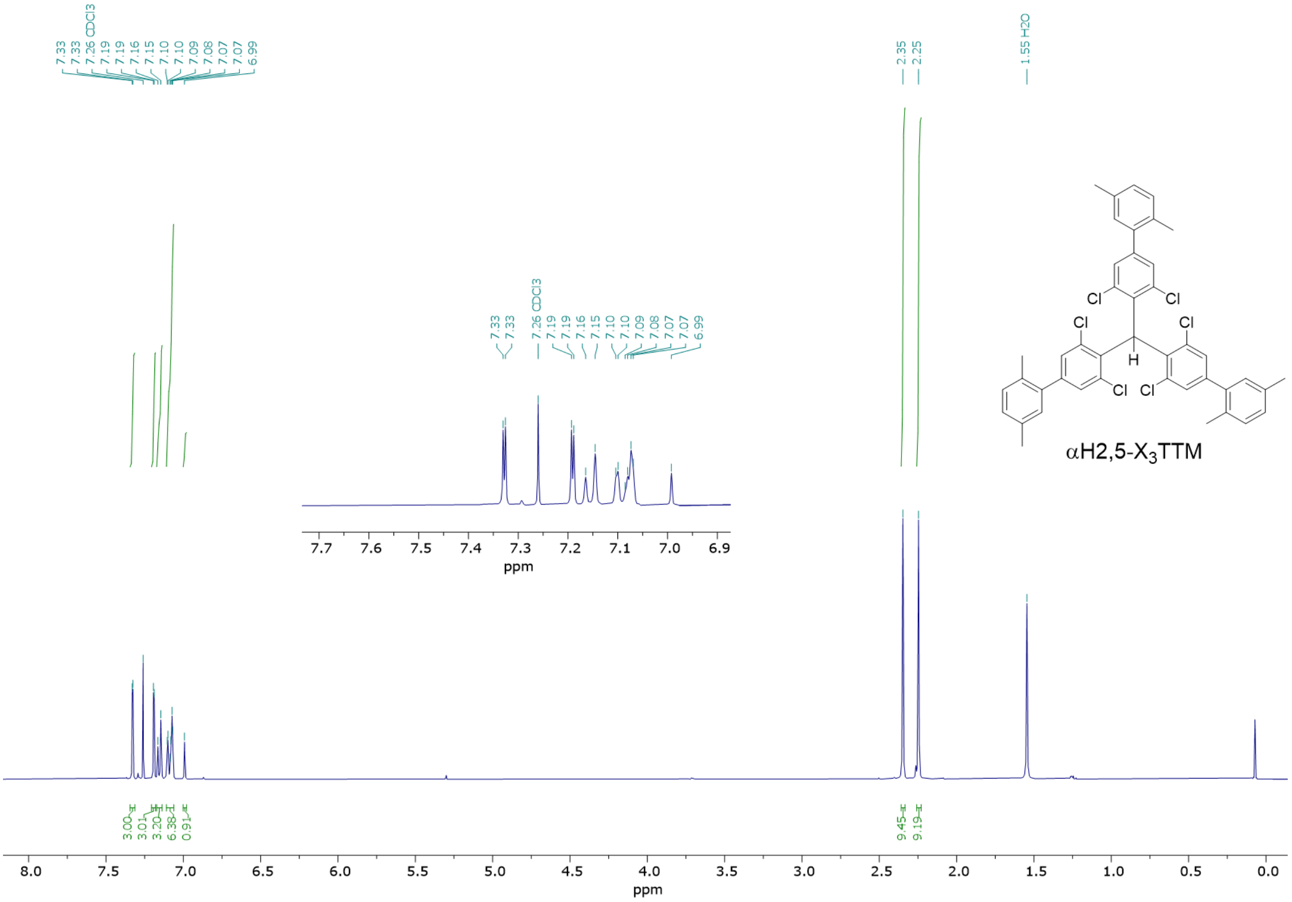
7.33
7.33
7.26 CDCl3
7.19
7.19
7.16
7.15
7.10
7.10
7.09
7.08
7.07
7.07
6.99
2.35
2.25
1.55 H2O
3.00
3.01
3.20
6.38
0.91
9.45
9.19
Cl
H
αH2,5-X3TTM
ppm

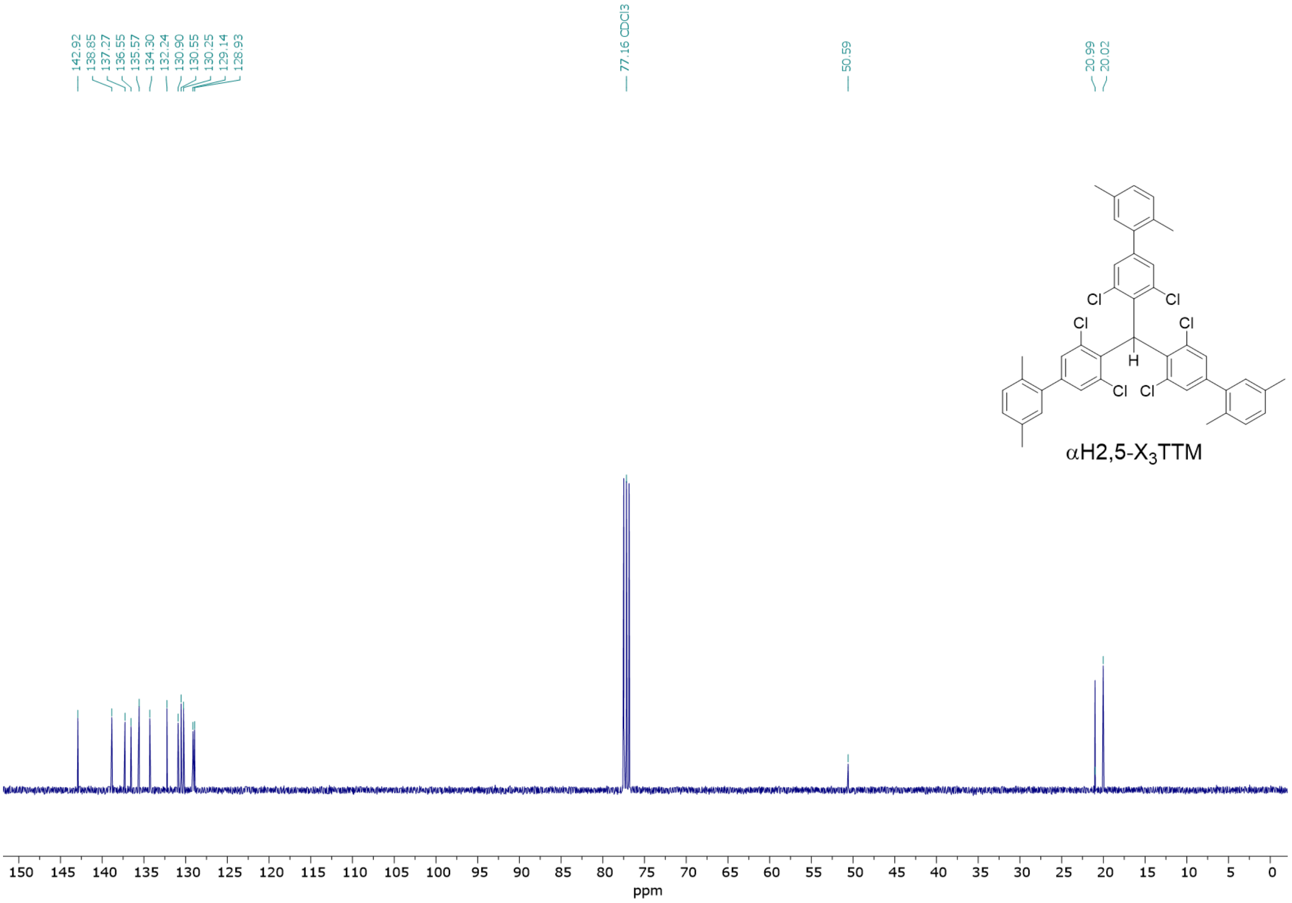
142.92
138.85
137.27
136.55
135.57
134.30
132.24
130.90
130.55
130.25
129.14
128.93
77.16 CDCl3
50.59
20.99
20.02
Cl
H
αH2,5-X3TTM
ppm

COSY

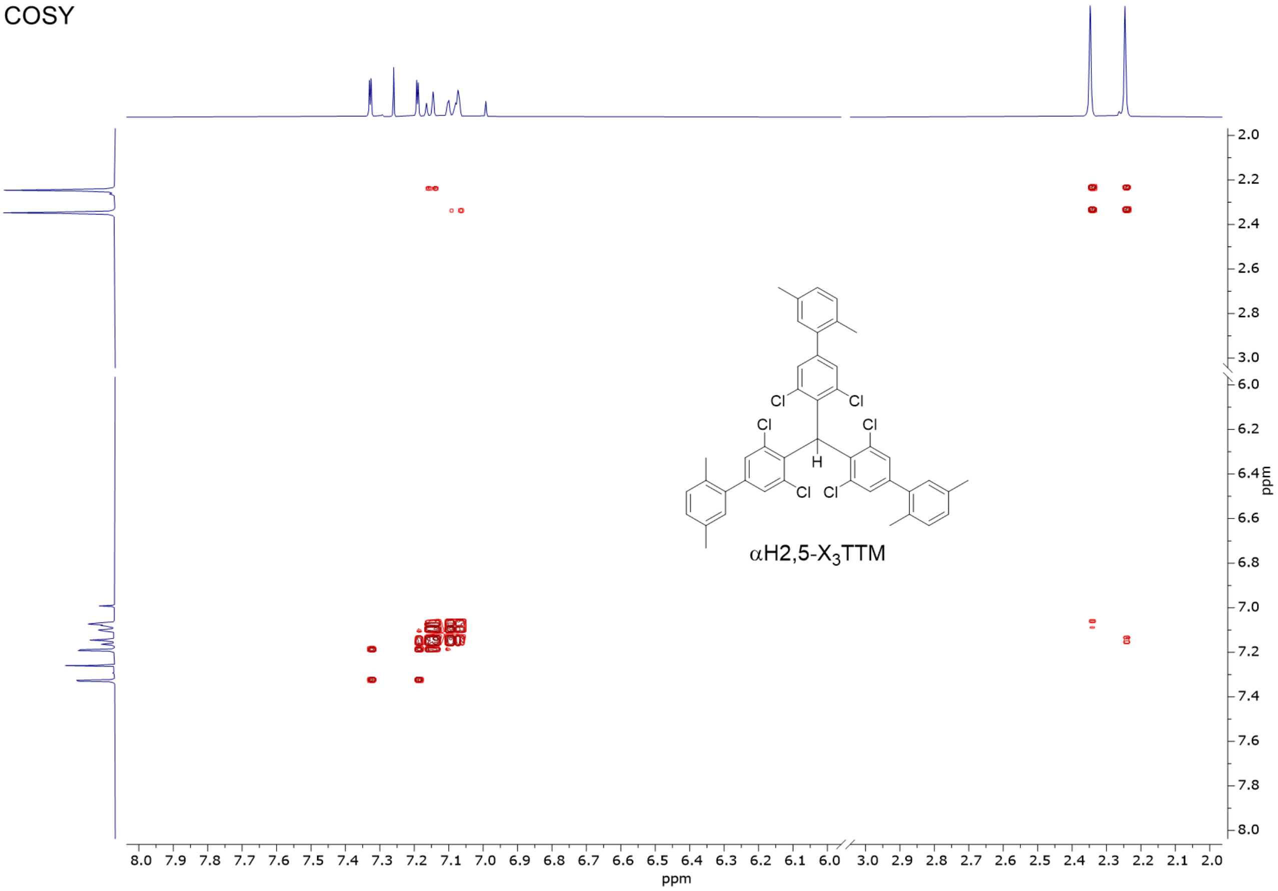

HSQC

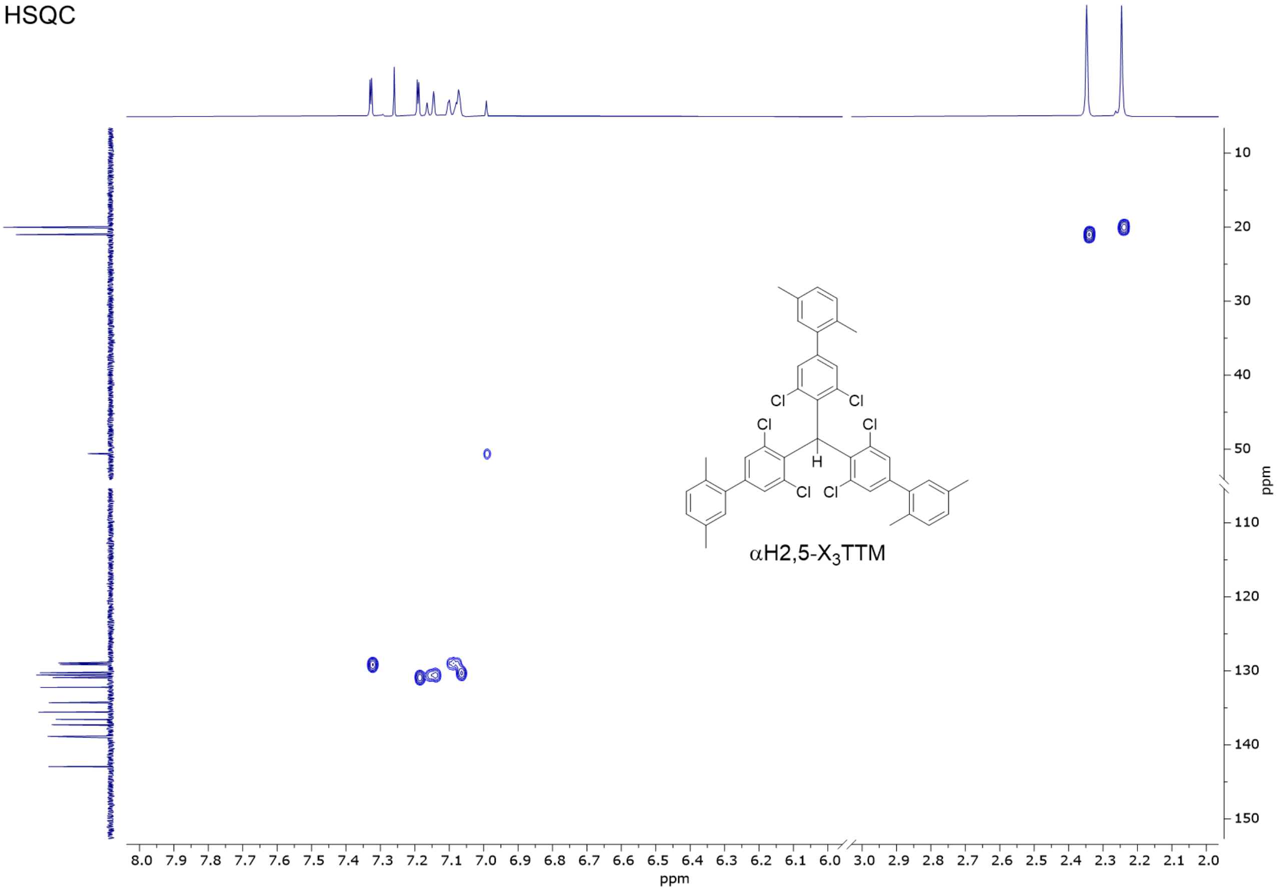

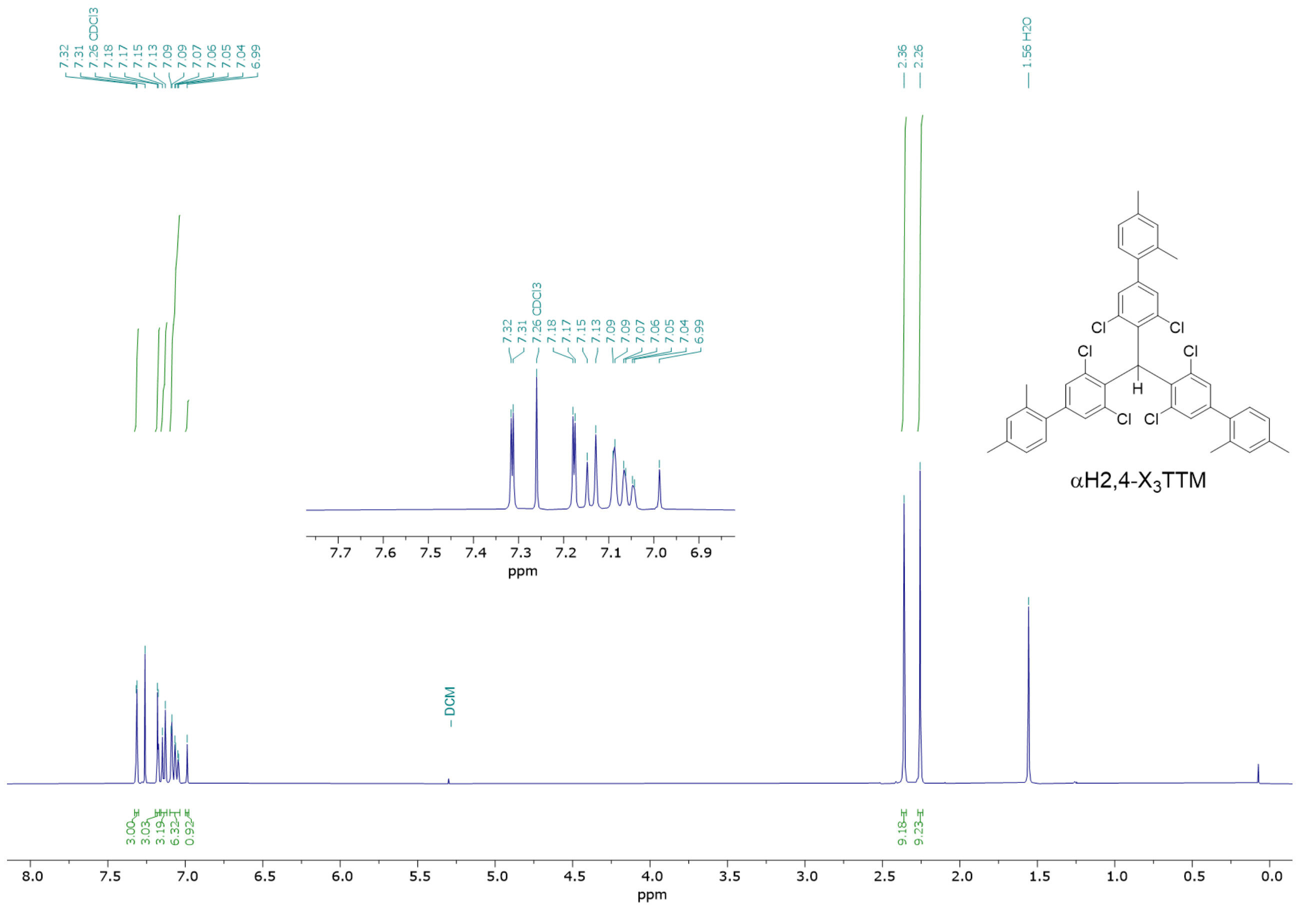

7.32
7.31
7.26 CDCl3
7.18
7.17
7.15
7.13
7.09
7.09
7.07
7.06
7.05
7.04
6.99
2.36
2.26
1.56 H2O
DCM
3.00
3.03
3.19
6.32
0.92
9.18
9.23
ppm
αH2,4-X3TTM

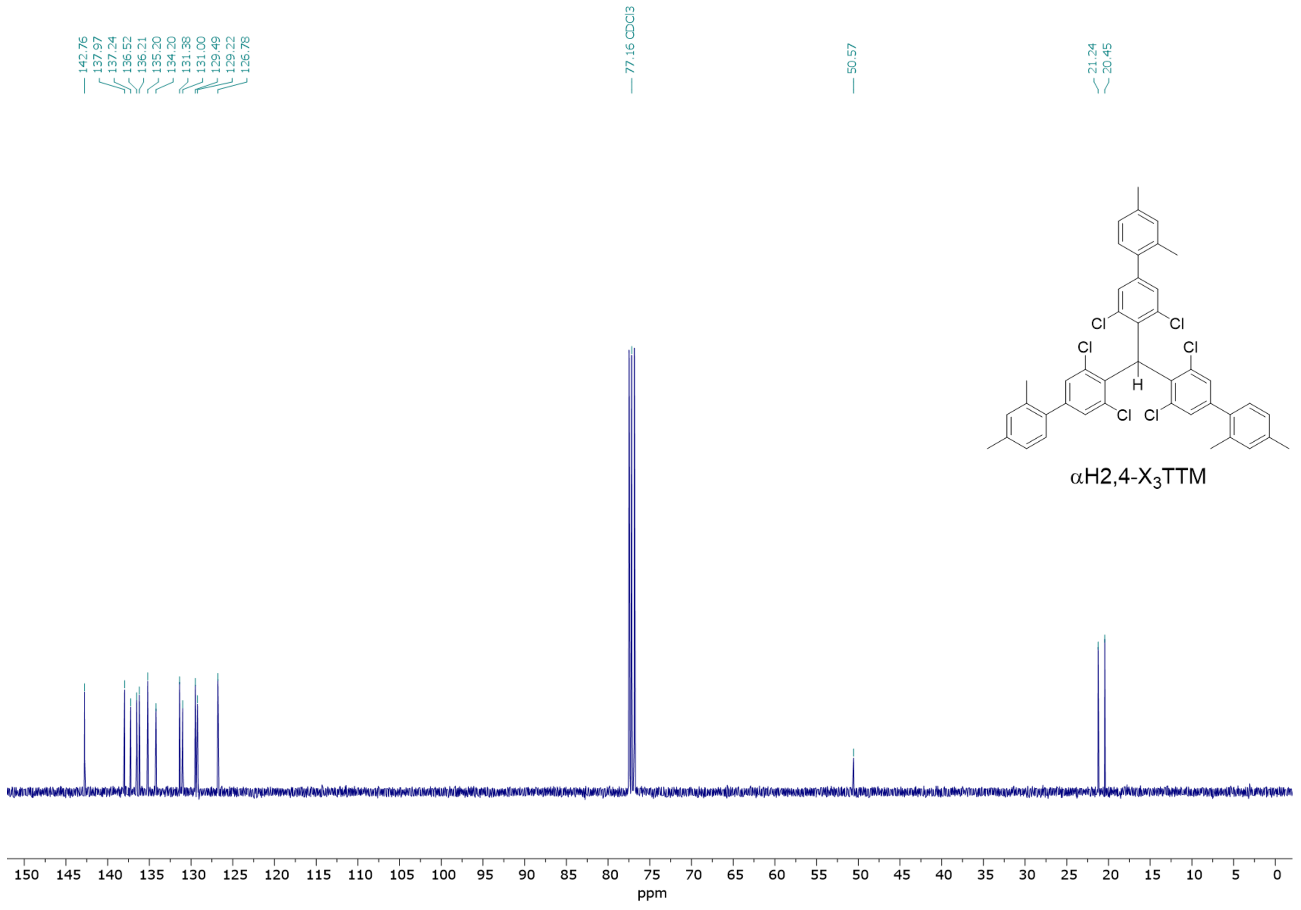

142.76
137.97
137.24
136.52
136.21
135.20
134.20
131.38
131.00
129.49
129.22
126.78
77.16 CDCl3
50.57
21.24
20.45
ppm
αH2,4-X3TTM

COSY

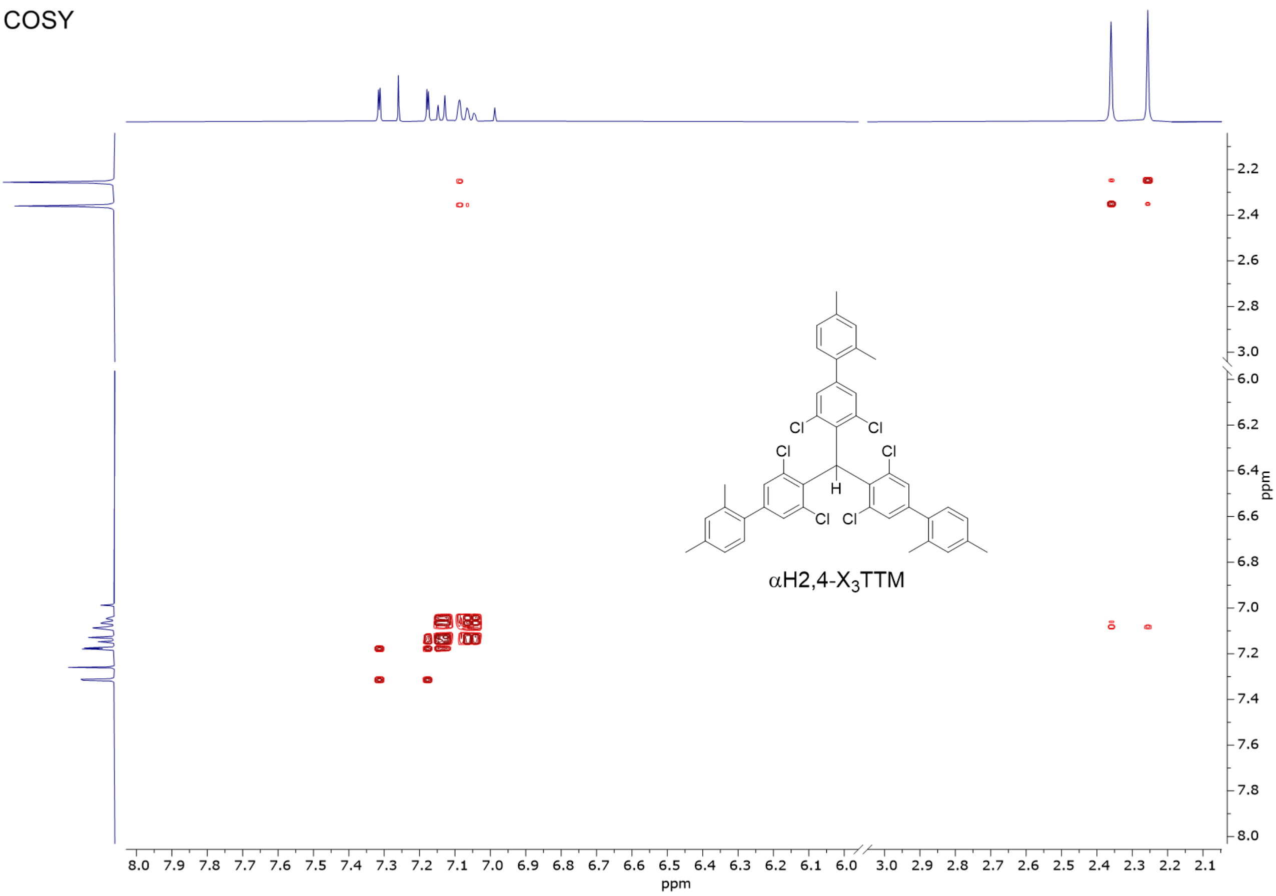

Cl
Cl
Cl
Cl
H
Cl
Cl
αH2,4-X3TTM
ppm

HSQC

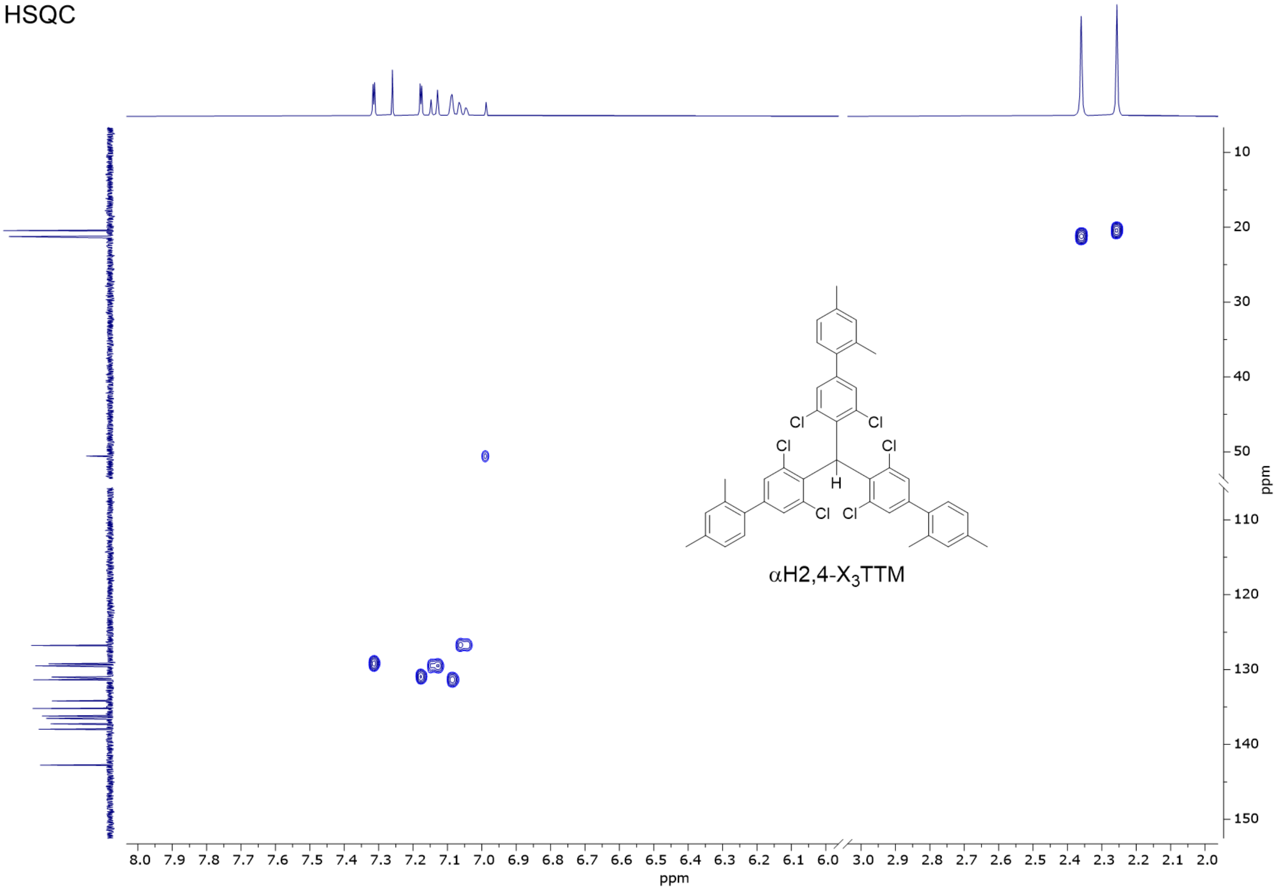

Cl
Cl
Cl
Cl
H
Cl
Cl
αH2,4-X3TTM
ppm

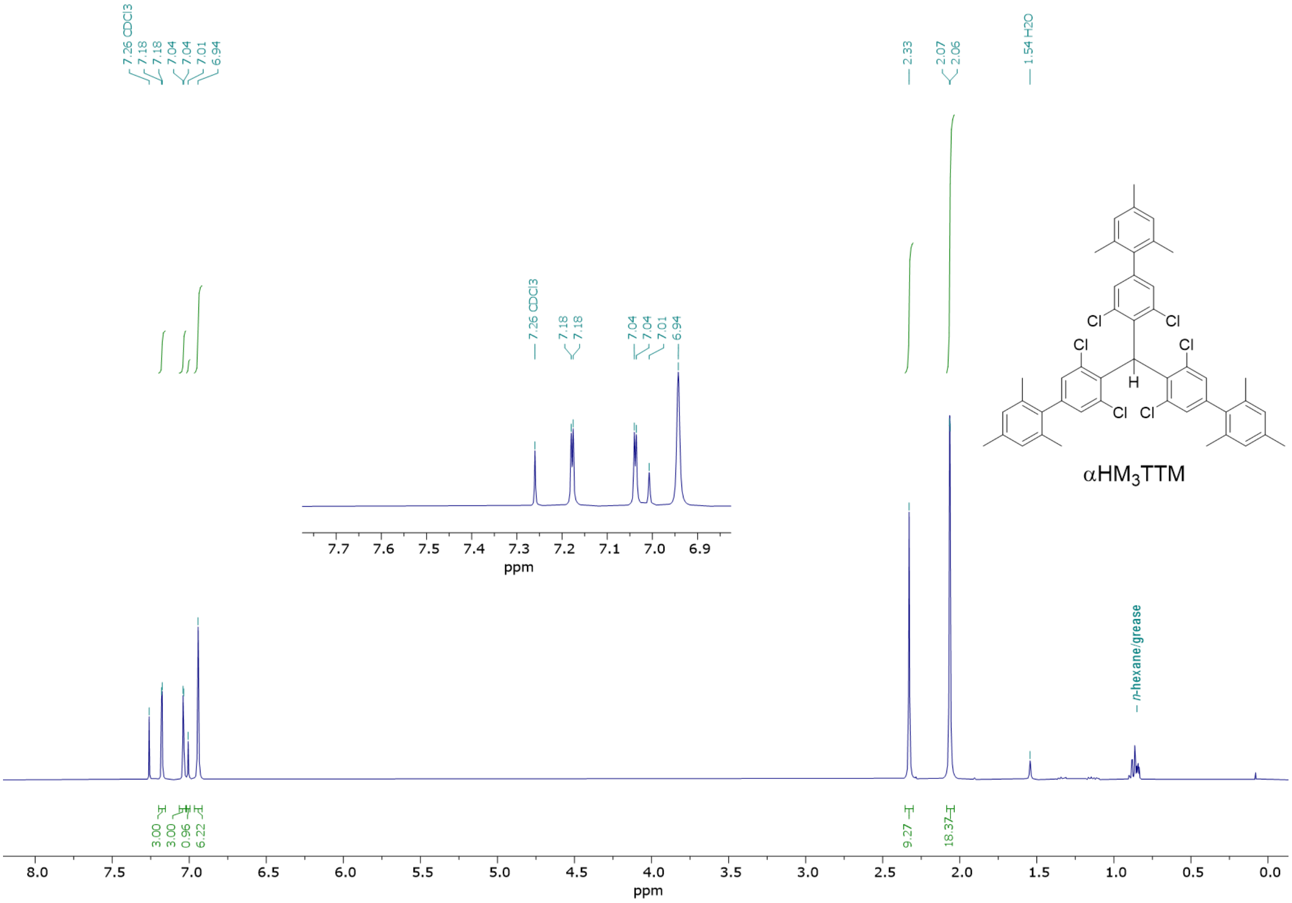
7.26 CDCl3
7.18
7.18
7.04
7.04
7.01
6.94
2.33
2.07
2.06
1.54 H2O
αHM3TTM
n-hexane/grease
3.00
3.00
0.96
6.22
9.27
18.37
ppm

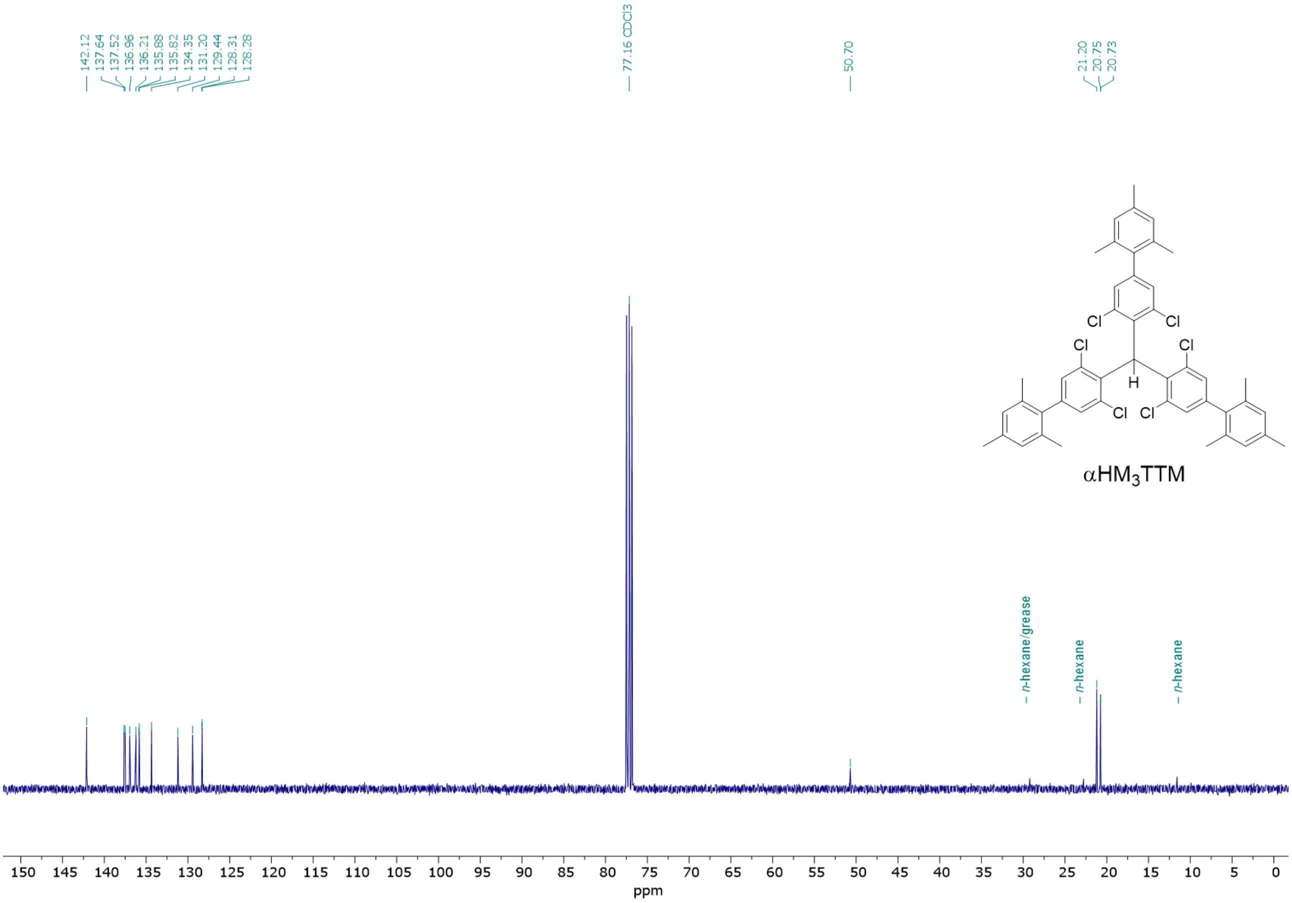
142.12
137.64
137.52
136.96
136.21
135.88
135.82
134.35
131.20
129.44
128.31
128.28
77.16 CDCl3
50.70
21.20
20.75
20.73
αHM3TTM
n-hexane/grease
n-hexane
n-hexane
ppm

COSY

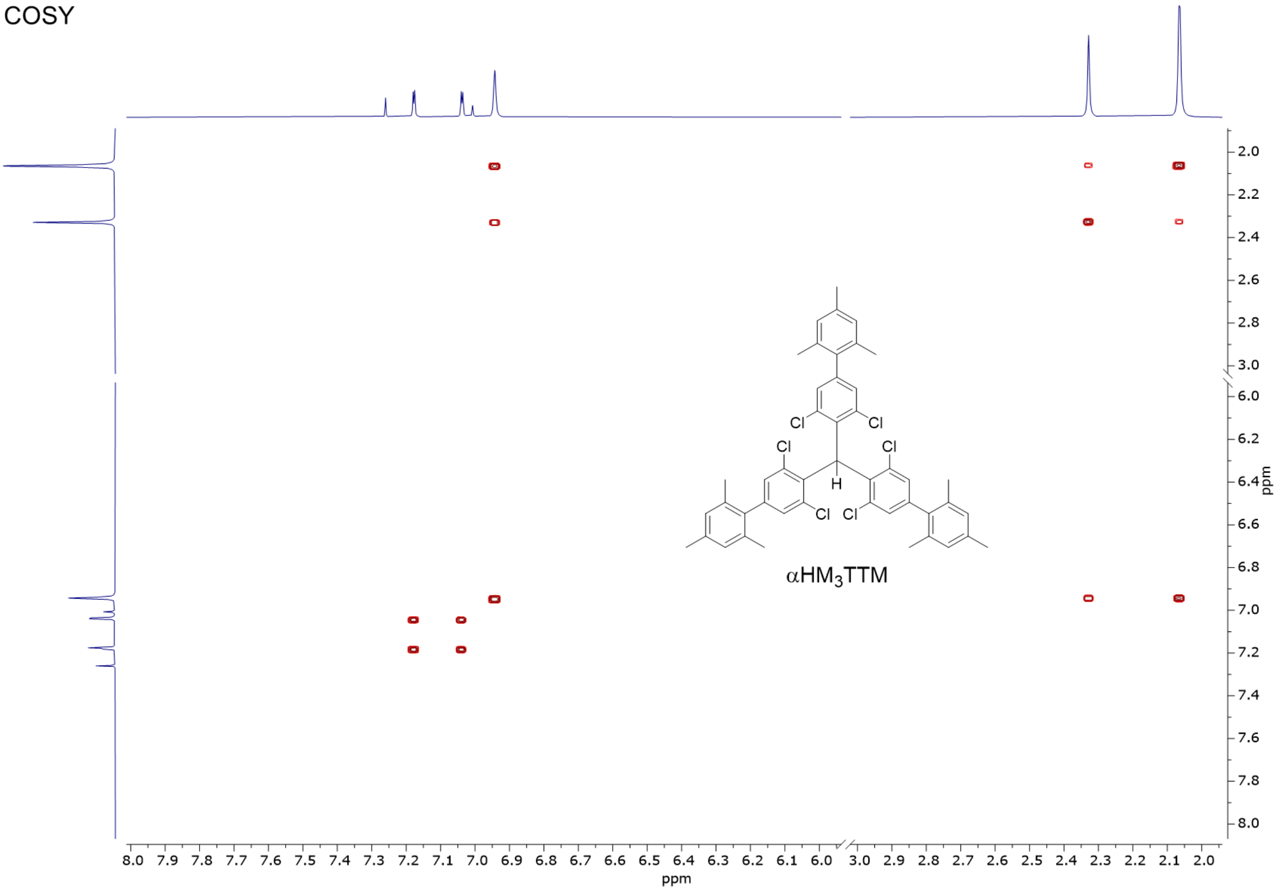
Cl
Cl
Cl
Cl
H
Cl
Cl
Cl
αHM3TTM
ppm
ppm

HSQC

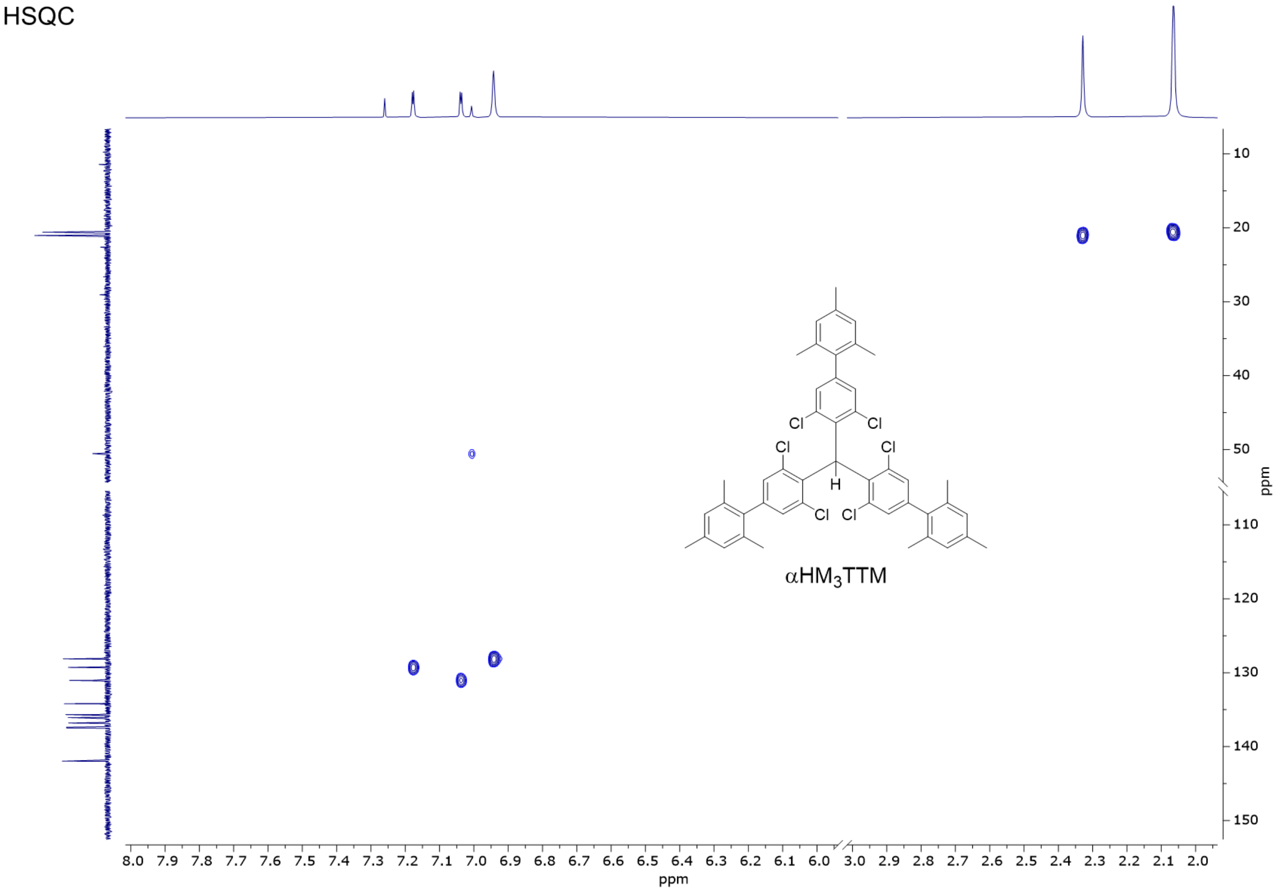
Cl
Cl
Cl
Cl
H
Cl
Cl
αHM3TTM
ppm
ppm

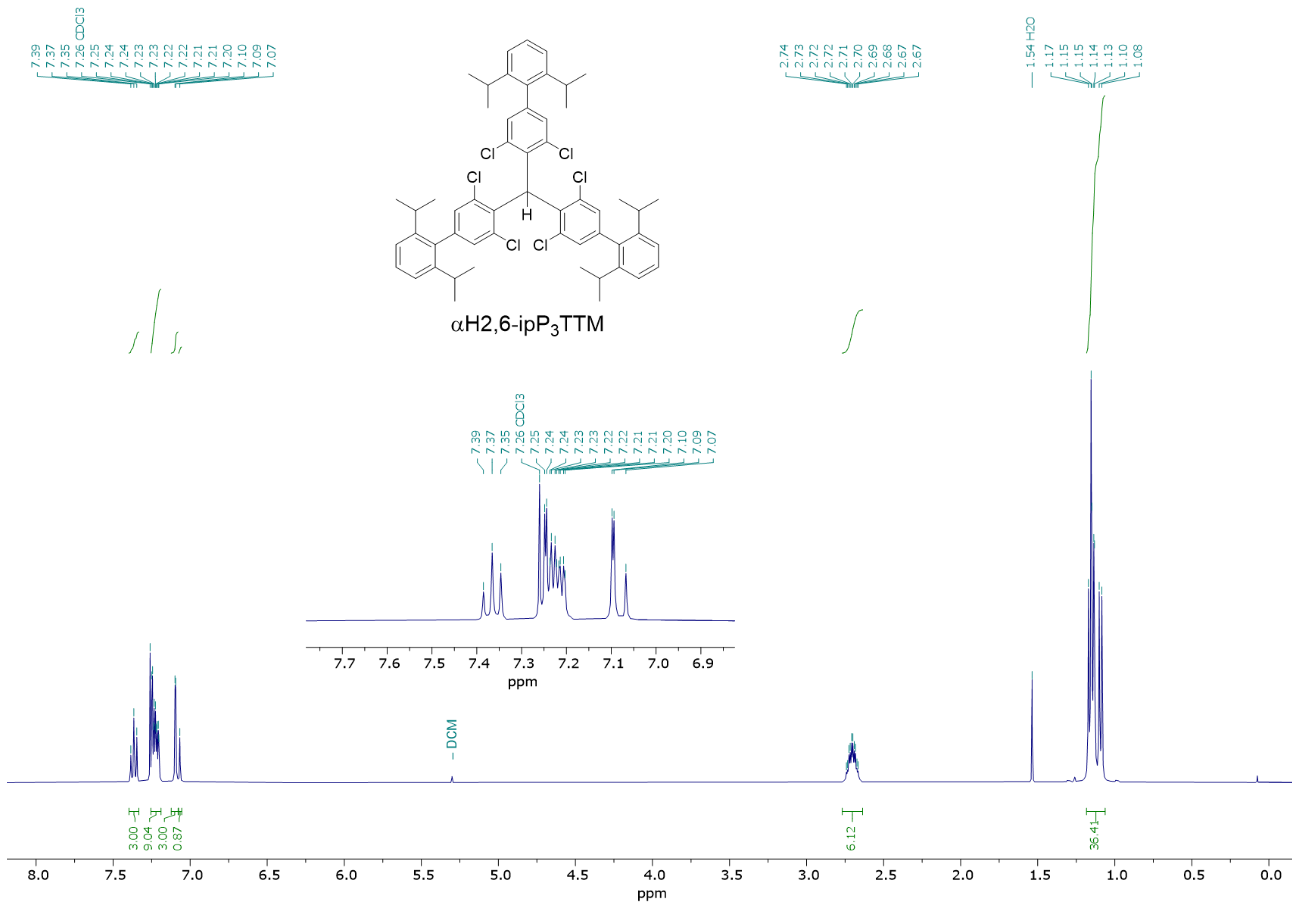
7.39 7.37 7.35 7.26 CDCl3 7.25 7.24 7.24 7.23 7.23 7.22 7.22 7.21 7.21 7.20 7.10 7.09 7.07
2.74 2.73 2.72 2.72 2.71 2.70 2.69 2.68 2.67 2.67
1.54 H2O 1.17 1.15 1.15 1.14 1.13 1.10 1.08
Cl
H
αH2,6-ipP3TTM
DCM
3.00 9.04 3.00 0.87
6.12
36.41
ppm

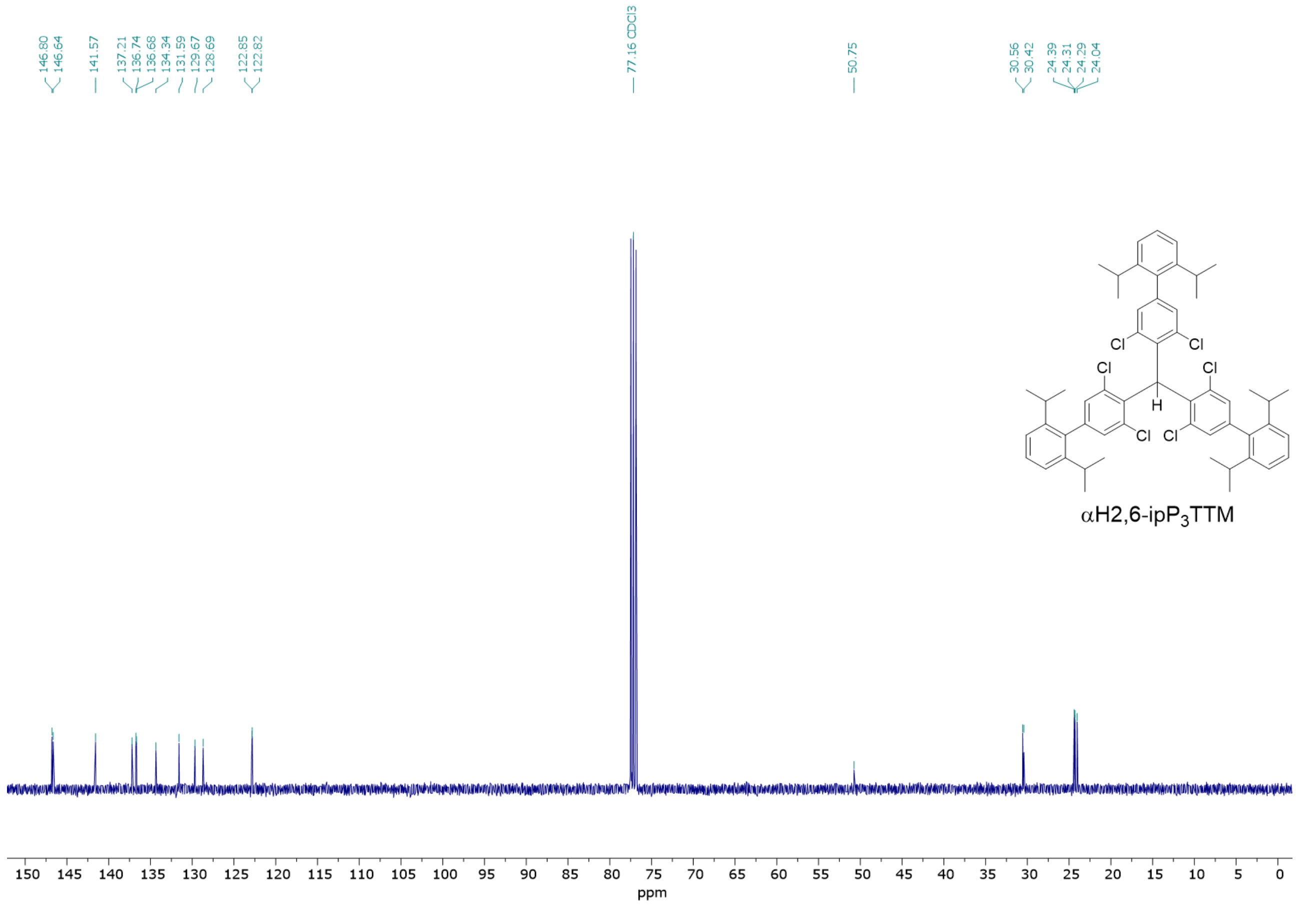
146.80 146.64 141.57 137.21 136.74 136.68 134.34 131.59 129.67 128.69 122.85 122.82
77.16 CDCl3
50.75
30.56 30.42 24.39 24.31 24.29 24.04
Cl
H
αH2,6-ipP3TTM
ppm

COSY

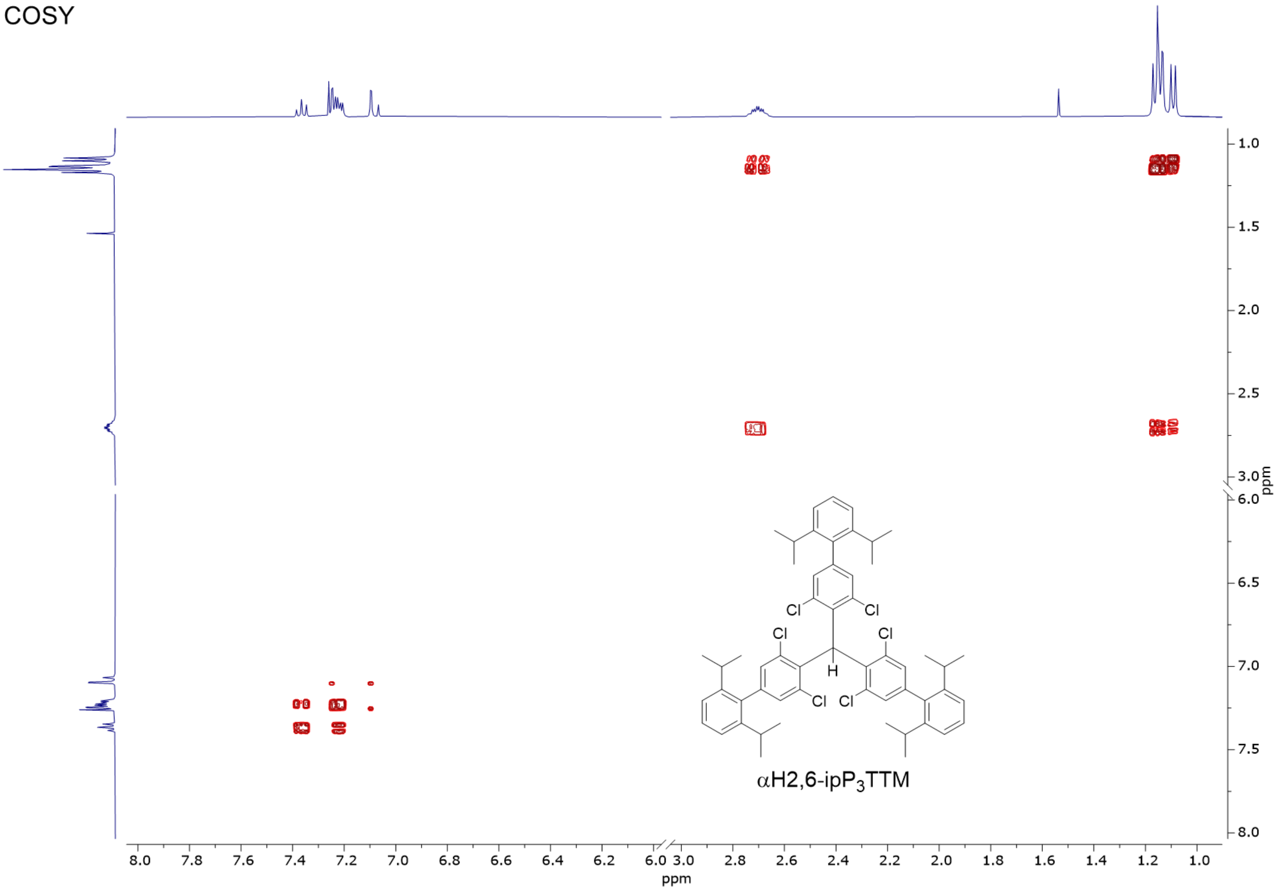

Cl
Cl
Cl
Cl
H
Cl
Cl
αH2,6-ipP$_3$TTM
ppm

HSQC

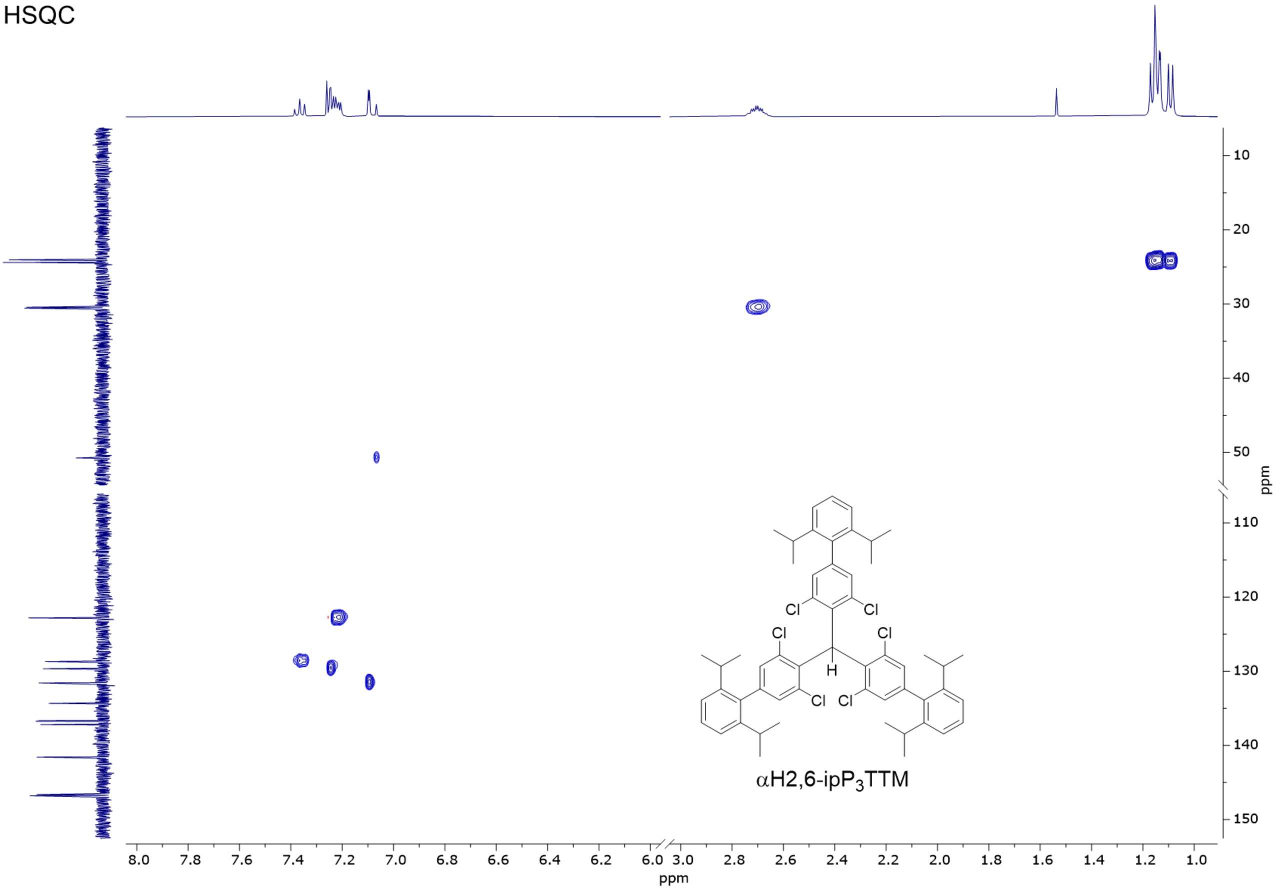

Cl
Cl
Cl
Cl
H
Cl
Cl
αH2,6-ipP$_3$TTM
ppm

^{1}H NMR spectra of the synthesized radicals

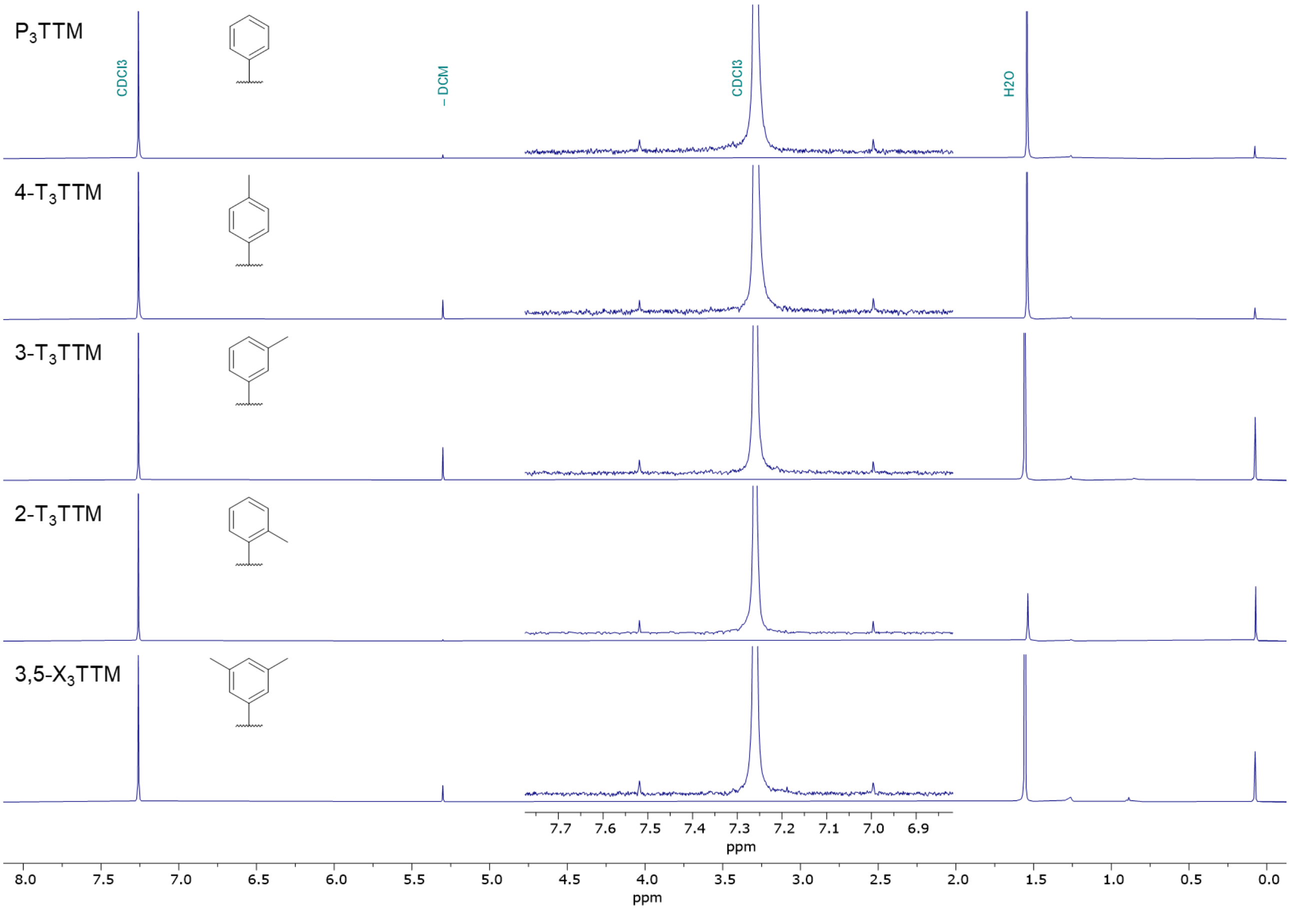

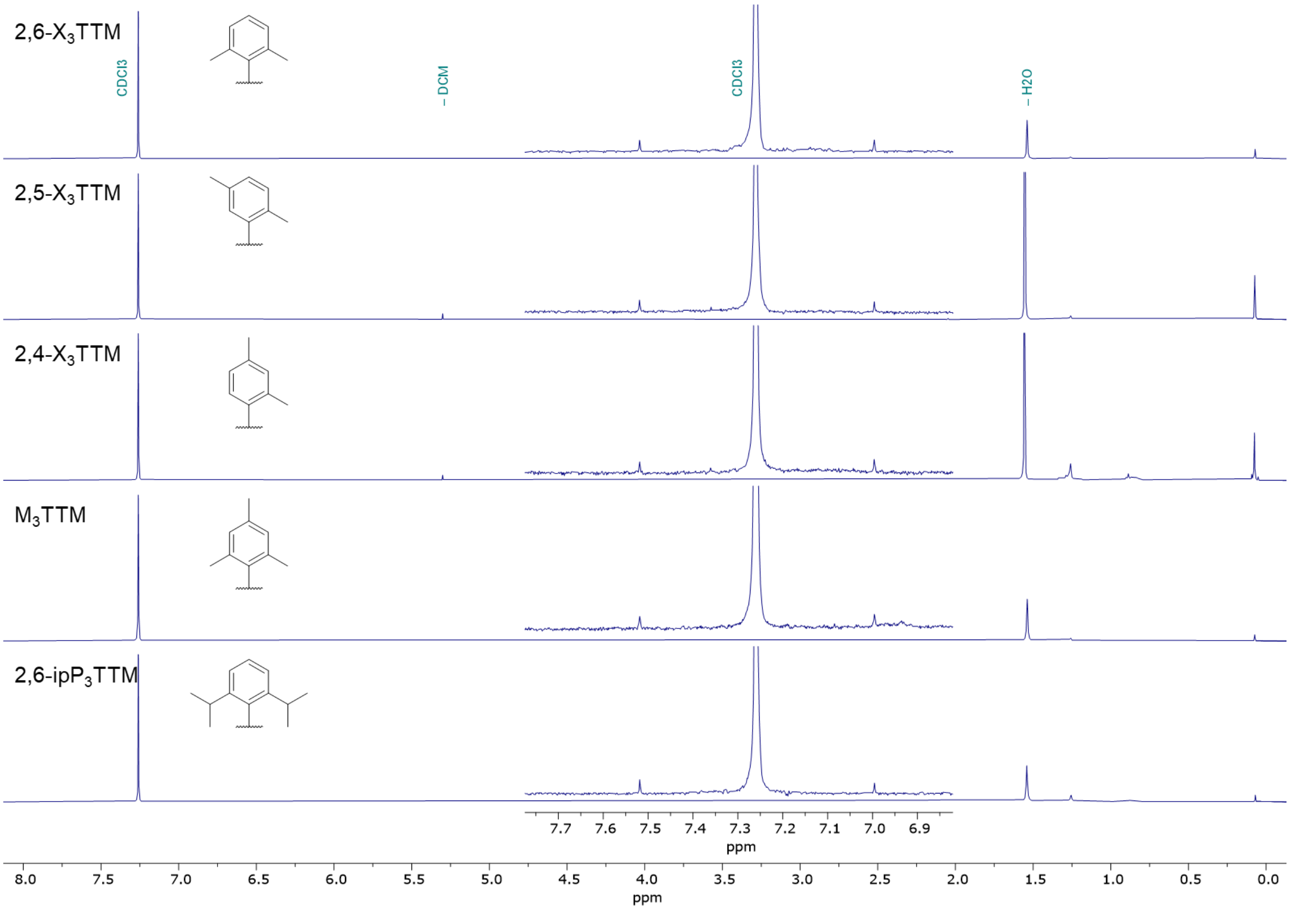

Supporting References

1. Murto, P.; Chowdhury, R.; Gorgon, S.; Guo, E.; Zeng, W.; Li, B.; Sun, Y.; Francis, H.; Friend, R. H.; Bronstein, H., Mesitylated trityl radicals, a platform for doublet emission: symmetry breaking, charge-transfer states and conjugated polymers. *Nat. Commun.* **2023**, *14,* 4147.
2. Roques, N.; Maspoch, D.; Wurst, K.; Ruiz-Molina, D.; Rovira, C.; Veciana, J., Three-Dimensional Six-Connecting Organic Building Blocks Based on Polychlorotriphenylmethyl Units—Synthesis, Self-Assembly, and Magnetic Properties. *Chem. Eur. J.* **2006**, *12*, 9238–9253.
3. Peng, Q.; Obolda, A.; Zhang, M.; Li, F., Organic Light-Emitting Diodes Using a Neutral π Radical as Emitter: The Emission from a Doublet. *Angew. Chem. Int. Ed.* **2015**, *54*, 7091–7095.
4. Spek, A., PLATON SQUEEZE: a tool for the calculation of the disordered solvent contribution to the calculated structure factors. *Acta Cryst.* **2015**, *C71*, 9–18.
